# Continuously tunable coherent pulse generation in a semiconductor laser

**DOI:** 10.1038/s41586-026-10387-w

**Published:** 2026-04-15

**Authors:** Urban Senica, Michael A. Schreiber, Marco Raffa, Paolo Micheletti, Mattias Beck, Christian Jirauschek, Jérôme Faist, Giacomo Scalari

**Affiliations:** 1 Institute for Quantum Electronics, ETH Zürich, Zurich, Switzerland; 2 John A. Paulson School of Engineering and Applied Sciences, Harvard University, Cambridge, MA USA; 3 TUM School of Computation, Information and Technology, Technical University of Munich (TUM), Garching, Germany

**Keywords:** Mode-locked lasers, Semiconductor lasers, Quantum cascade lasers, Frequency combs, Integrated optics

## Abstract

In a laser, the control of its spectral emission depends on the physical dimensions of the optical resonator, restricting it to a set of discrete cavity modes at specific frequencies^1–4^. Without modifying the optical cavity, this results in substantial gaps in the obtainable laser emission spectrum, as well as a fixed repetition rate, limiting the device’s usability in various experiments and applications where a considerable degree of tunability is required in the spectral or temporal domain. Here we overcome this fundamental limit by demonstrating a monolithic semiconductor laser^5–7^ with a continuously tunable repetition rate from 4 GHz up to 16 GHz, by using a microwave driving signal that induces a spatiotemporal gain modulation along the entire laser cavity^8,9^, generating intracavity mode-locked pulses^10–13^ with a continuously tunable group velocity^14^. At the output, frequency combs^15,16^ with continuously tunable mode spacings are generated in the frequency domain, and coherent pulse trains with continuously tunable repetition rates are generated in the time domain^17^. Our results pave the way for fully tunable chip-scale lasers and frequency combs, which will be advantageous for use in a diverse variety of fields, from fundamental studies to applications such as high-resolution and dual-comb spectroscopy^18,19^.

## Abstract (web summary)

A monolithic mode-locked semiconductor laser with a continuously and widely tunable repetition rate is achieved by using a microwave driving signal that induces a spatiotemporal gain modulation along the entire laser cavity.

## Main

Since the first demonstrations of the maser^1^ and optical laser^2^, a resonant optical cavity has been a crucial component of a given laser system, providing a set of cavity resonances that can be excited and amplified in combination with a gain medium. The possibility to excite one or many cavity modes is intrinsically related to the laser gain medium bandwidth, the physical dimensions of the cavity and the reflection coefficients of its output couplers, which have a fundamental role in the equations governing the laser dynamics^3^. In the simplest configuration of a Fabry–Pérot laser cavity with two partially reflective mirrors^4^, the frequency spacing between neighbouring laser modes, or repetition rate (*f*_rep_), is determined by the cavity resonances and is inversely proportional to the cavity length *L* through the relation ${f}_{\mathrm{rep}}=\frac{c}{2{n}_{{\rm{g}}}L}$, where *c* is the speed of light in vacuum and *n*_g_ is the group refractive index of the optical mode^3^.

If the laser modes are locked through a nonlinear optical process, for example, four-wave mixing, a spectrum with equidistant modes is generated. These can be described by the relation *f*_*n*_ = *f*_ceo_ + *n* × *f*_rep_, where *n* is an integer and the carrier-envelope offset frequency *f*_ceo_ is related to the chromatic dispersion of the optical cavity. When both *f*_ceo_ and *f*_rep_ are stabilized and locked to a referenced standard, a frequency comb is generated^15,16^, which is a set of perfectly equidistant and phase-locked lasing modes in the frequency domain, corresponding to a perfectly periodic output waveform envelope in the time domain. Depending on the intermodal phases, the laser output in the time domain will consist of a train of (ultrashort) optical pulses (amplitude-modulated combs)^10–12^ or of a more complex periodic waveform (frequency-modulated combs)^20–23^.

The repetition rate *f*_rep_ can be tuned by modifying the cavity resonances, either by changing the cavity length *L* or the material permittivity *ε*. With harmonic mode-locking, integer harmonics of *f*_rep_ can be excited^24,25^; however, these provide only a discrete change. In a monolithic configuration, where the cavity dimensions are constant, *f*_rep_ can be continuously changed by a few per cent at most^26^.

## Frequency-tunable active mode-locking

**Fig. 1:**
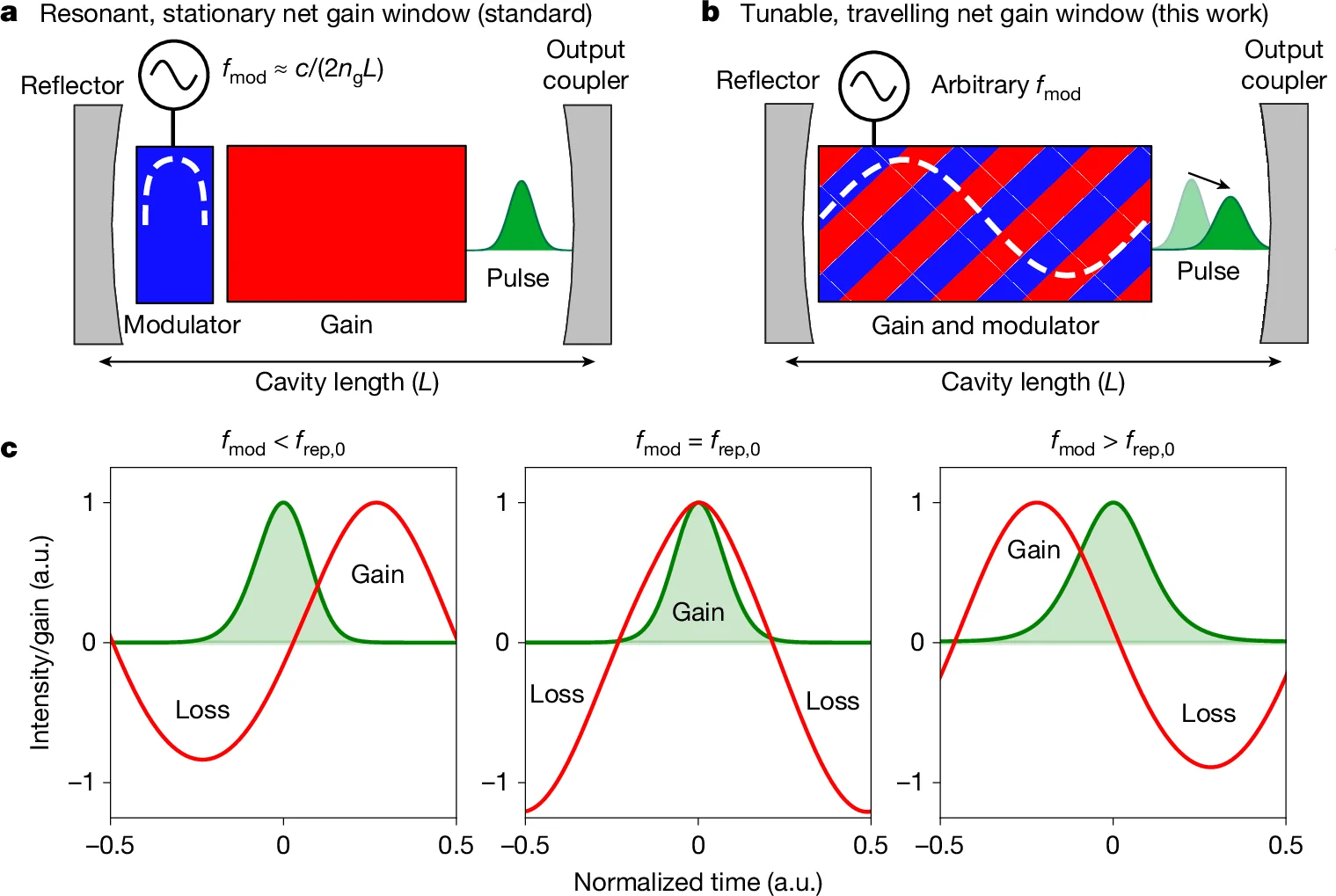
Overview of the general operating principle. **a**, In standard active mode-locking, the spatially confined modulator is synchronized to the cavity repetition rate, opening a stationary net gain window. **b**, In our work, tunable active mode-locking is enabled by a spatiotemporal gain modulation along the entire lasing cavity. The resulting standing modulation wave (white dashed line) has a phase velocity that is proportional to the modulation frequency, generating a tunable travelling net gain window. **c**, Results of our numerical model in three different regimes in a co-propagating frame (averaged over the forward propagation cycle). Any shift between the peak of the optical pulse and the modulation wave results in a gain/loss gradient throughout the optical pulse, effectively speeding it up or slowing it down, such that the new repetition rate is synchronized to the external modulation frequency. Source Data

In standard active mode-locking, a modulator is placed within the laser cavity, as illustrated in Fig. 1a. By modulating the optical gain (or losses), a short net gain time window opens, allowing for the generation of short pulses. The modulator is typically separated from the gain and is spatially confined to a small fraction of the laser cavity. The modulation frequency needs to be resonant with the cavity repetition rate, as a spatially stationary net gain window is generated. Detuning the modulator frequency by a substantial fraction of the repetition rate hinders the formation of short pulses, as the modulator cannot affect the light propagation throughout most of the laser cavity, falling out of sync with the detuned net gain window.

Here we propose a regime where the repetition rate of a mode-locked semiconductor laser can be tuned continuously and substantially both above and below the fundamental frequency defined by the cavity length. As sketched in Fig. 1b, this is enabled by a spatiotemporal gain modulation along the entire laser device via an external microwave signal that modulates the laser bias. The modulator induces a spatially extended gain/loss wave, resulting in a travelling net gain window that affects the optical pulse propagation along the entire device length. The distributed modulator should generate a standing gain/loss modulation wave as this way its phase velocity, that is, the propagation velocity of the travelling net gain window, is proportional to the modulation frequency. In a real device, this can be implemented by injecting microwaves at one end of the low-loss waveguide and imposing reflective boundary conditions, such that the interference between counterpropagating microwaves generates a (quasi-) standing wave.

We developed an analytical model by expanding the Haus-type master equation framework presented in refs. ^11,17^. These models describe the optical pulse by its round-trip-averaged envelope, giving Gaussian pulse solutions for arbitrary values of frequency detuning. We describe the evolution of the forward- and backward-propagating electric-field envelopes *E*^±^ in a co-propagating frame, neglecting chromatic dispersion (Methods):

1 $$\pm {\partial }_{x}{E}^{\pm }={\delta }^{\pm }{\partial }_{\tau }{E}^{\pm }+\frac{1}{2}({g}^{\pm }+{g}_{\omega }{\partial }_{\tau }^{2}-a){E}^{\pm },$$

with the detuning parameter ${\delta }^{\pm }={({v}_{{\rm{m}}}^{\pm })}^{-1}-{v}_{\mathrm{g,0}}^{-1}$ (the difference between inverse optical group velocities in the modulated and unmodulated cavity), saturated time-modulated gain *g*^±^, spectral gain bandwidth in parabolic approximation *g*_*ω*_ and loss coefficient *a*. By assuming a Gaussian pulse shape, a stationary analytical solution can be obtained, which relates the detuning *δ*^±^ with the gain slope ${g}_{1}^{\pm }$ and the full-width at half-maximum pulse duration *T*^±^:

2 $${\delta }^{\pm }={g}_{1}^{\pm }{({T}^{\pm })}^{2}/(8\,{\rm{ln}}\,2).$$

We can develop an intuitive understanding of the tunable mode-locking mechanism. In Fig. 1c, we plot the numerical model solutions, averaged over the forward propagation cycle, for three different typical regimes in the co-propagating frame. These have been obtained with a semiclassical Maxwell-density matrix formalism^9^ (Methods). As the modulation period is determined by the external modulation frequency *f*_mod_, the travelling net gain window completes a full round trip in time 1/*f*_mod_. When the modulation frequency matches the natural repetition rate (*f*_mod_ = *f*_rep,0_), the group velocity of the optical pulse and the phase velocity of the modulation wave are also matched naturally. Their peaks are aligned (synchronized), converging to standard active mode-locking. However, when the modulation frequency is positively detuned (*f*_mod_ > *f*_rep,0_), an optical pulse initially propagating with the natural group velocity will be slower than the travelling gain/loss window, thus experiencing a gain/loss gradient, that is, more gain at its leading than its trailing edge, which will experience loss. For sufficiently large modulation amplitudes, this will continuously deform the propagating pulse, effectively increasing its group velocity and matching it to the increased velocity of the travelling net gain window. The opposite happens with negative detuning (*f*_mod_ < *f*_rep,0_), that is, the pulse will be initially faster than the gain/loss wave and effectively slowed down. These different regimes are clearly visible in Fig. 1c, where the optical pulse is either aligned with, ahead of or lagging behind the peak of the modulation wave. In all cases, the average group velocity of the intracavity optical waveform envelope will be adjusted such that the round-trip time is perfectly synchronized to the modulation frequency. By comparing the results of the numerical and analytical models, it can be shown that the pulse pulling owing to the gain slope explains the continuous tunability (Methods). Even for large values of frequency detuning, the pulse remains stably confined within the net gain window, as predicted in ref. ^17^. A coherent pulse train with an arbitrary repetition rate can be synthesized by tuning the microwave driving signal over a wide range of frequencies. In the frequency domain, this generates frequency combs with a continuously tunable mode spacing. The tunable mode-locked laser is then no longer limited to lasing on the usual longitudinal cavity modes imposed by the device dimensions. Instead, virtually any discrete lasing frequency within the gain bandwidth can be excited, as the frequencies of the lasing lines are simply a Fourier transform of the continuously tunable periodic pulse train.

## Experimental platform

Experimentally, we use a broadband planarized terahertz quantum cascade laser (THz QCL)^7,27^. QCLs are electrically pumped semiconductor lasers based on intersubband transitions^5^, operating in the mid-infrared^28^ and terahertz^6^ regions. Their ultrafast dynamics and large optical nonlinearities enable locking broadband emission spectra into coherent frequency combs^20,29,30^, which is useful in spectroscopy and sensing^18,19^.

**Fig. 2:**
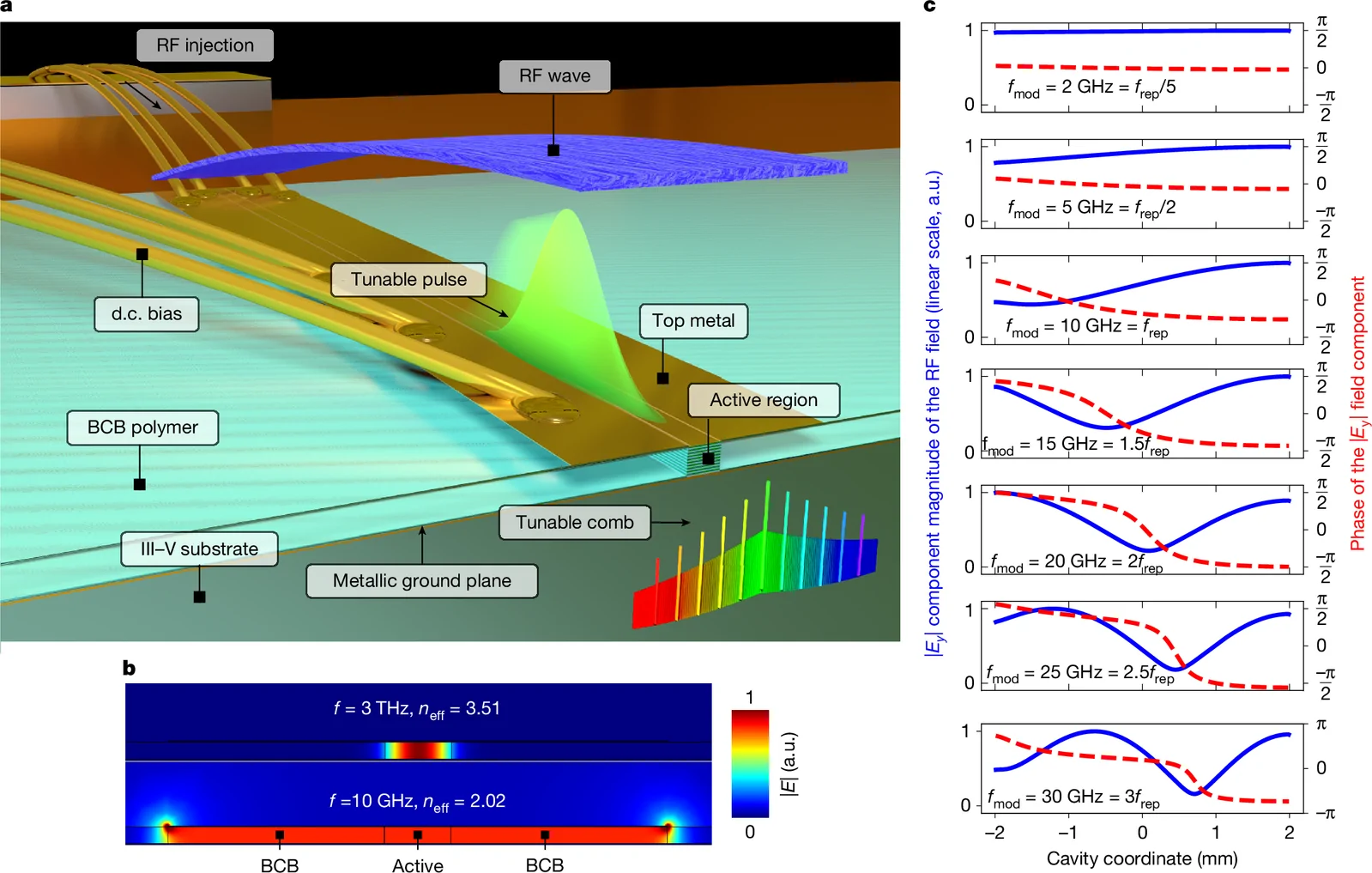
Device geometry and basic properties. **a**, Illustration of the experimental platform, where microwaves are injected into a planarized waveguide with a semiconductor laser core (THz QCL). An RF wave (blue) is formed along the entire lasing cavity, generating intracavity mode-locked pulses (green) with a tunable group velocity. **b**, Simulated two-dimensional waveguide cross-section electric-field intensity profiles for the propagating electromagnetic mode at the lasing frequency (3 THz, confined to the active core) and at microwave frequencies (10 GHz, spread across the planarized waveguide). *n*_eff_ is the effective mode index, which quantifies the phase delay of the propagating waveguide mode per unit length. **c**, Three-dimensional simulation results of the RF field distribution along the centre of a 4-mm-long active waveguide, when injected from the back facet (left side), forming a frequency-dependent wave pattern. The amplitude and phase are shown in blue and red, respectively. Source Data

As illustrated in Fig. 2a, the device consists of an active material waveguide embedded in the low-loss polymer benzocyclobutene (BCB) and sandwiched between two metallic layers, vertically confining the optical and microwave modes and acting as electrical contacts. This is advantageous in terms of low waveguide losses, efficient heat dissipation and excellent microwave properties. As shown in Fig. 2b, while the optical mode is confined within the central active waveguide, the microwave mode spreads across the entire extended top and bottom metallization. With a reduced spatial overlap with the lossy central active waveguide (arising from its finite vertical conductivity), the planarized waveguide acts as a low-loss, low-impedance microstrip transmission line for microwaves^7,8^. Previously, freely tunable sideband generation was achieved by strongly injecting microwaves in single-mode, unplanarized double metal THz QCLs^31^. As microwaves are injected via a set of short bonding wires at the back waveguide facet, they propagate and reflect at the front facet, forming a frequency-dependent wave pattern along the entire cavity. As shown in Fig. 2c, the radio-frequency (RF) field distribution shows a maximum at the front waveguide facet. For low frequencies (*f*_mod_ ≪ *f*_rep,0_), the modulation is in a quasi-static regime with a nearly uniform field. With increasing frequency, a number of minima and maxima form along the cavity, and the wave distributions resemble an oscillating cosine function. These properties allow for an efficient spatiotemporal gain modulation for an arbitrary microwave modulation frequency, regardless of any mismatch between the waveguiding properties at microwave and terahertz frequencies, such as their effective mode indices and propagation velocities. This differs from many other integrated microwave photonics devices, where more elaborate phase-matching schemes are required for an efficient co-propagation of optical and microwave modes at specific frequencies^32^.

## Coherent measurements and simulations

We investigated a 6-mm-long planarized ridge waveguide, where the central active waveguide is 40-μm wide and the extended top metallization has a width of 300 μm (Methods). The device is measured at a heat-sink temperature of 40 K, and the natural repetition rate is *f*_rep,0_ = 6.61 GHz. We performed a modulation frequency sweep study, where the direct-current (d.c.) laser bias is kept close to the laser threshold, and a strong microwave signal (typically +30 dBm at the source) is injected at the back facet. To assess the coherence and reconstruct the time-domain output, we performed shifted wave interference Fourier transform (SWIFT) spectroscopy^29,33^. Under resonant driving conditions, the microwave injection frequency is close (within a few megahertz) to the natural repetition rate, generating modulation sidebands, injection-locking the longitudinal cavity modes, stabilizing the periodic laser output, and broadening the emission spectrum^34,35^ (Extended Data Fig. 2).

**Fig. 3:**
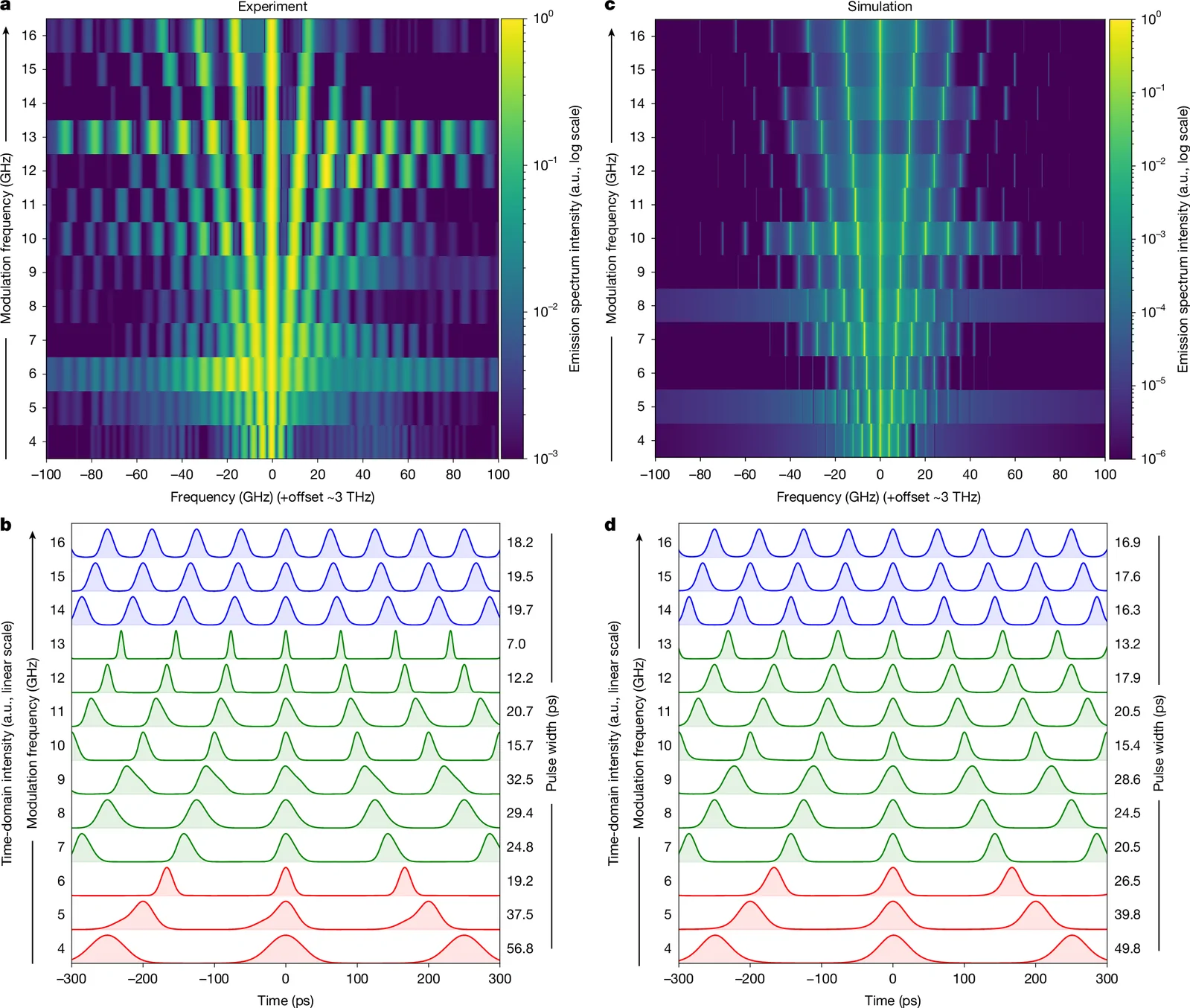
Spectral and time-domain measurement and numerical simulation results. **a**, Measured emission spectra for modulation frequencies between 4 GHz and 16 GHz, where the spectra are aligned and zoomed around 200 GHz of the strongest central mode. **b**, The reconstructed time-domain intensity profiles reveal that a set of coherent pulse trains is generated, where the tunable repetition rate matches the microwave modulation frequency. **c**,**d**, The simulated emission spectra (**c**) and time-domain intensity profiles (**d**) show excellent agreement with experimental results, displaying continuously tunable mode spacings and repetition rates, synchronized to the microwave modulation frequency between 4 GHz and 16 GHz. The time-domain results in panels **b** and **d** are colour-coded to distinguish between different regimes of the modulation frequency: *f*_mod_ < *f*_rep,0_ (red), *f*_rep,0_ < *f*_mod_ < 2*f*_rep,0_ (green), and *f*_mod_ > 2*f*_rep,0_ (blue).

We can generate tunable emission spectra where the mode spacing matches the microwave modulation frequency. These were obtained also for extreme values of frequency detuning, ranging from nearly *f*_rep,0_/2 to well above 2*f*_rep,0_. The measurements were performed over a wide modulation frequency range between 4 GHz and 16 GHz with a step of 1 GHz, corresponding to a relative tuning range of 400%. In Fig. 3a, we show a two-dimensional colour map of the measured spectra, aligned and zoomed within 200 GHz of the strongest central mode (complete SWIFT spectroscopy results are in Extended Data Fig. 3). The tunable mode spacing is clearly visible as opening lines on the spectral map. As shown in Fig. 3b, a set of coherent pulse trains with a tunable repetition time is generated in the time domain. The individual pulse durations are shorter for smaller frequency detunings from the natural repetition rate (or its integer multiples) owing to a resonant effect, and for higher modulation frequencies owing to shorter modulation periods. The slight asymmetry and its orientation in some of the pulse shapes (*f*_mod_ = 5, 9, 11 GHz) is a signature of the pulse formation mechanism envisioned in ref. ^17^.

Such detailed features of the detuned pulse solutions can be recovered in numerical simulations of the modulated laser cavity (Fig. 3c,d), showing excellent agreement with experimental data. The simulated comb linewidth is far below the numerical resolution limit (<1 MHz, Fourier transform of 7,000 round trips), and coherence was verified by evaluating the power and phase noise quantifiers^36^. Despite biasing the device close to the lasing threshold, often associated with noisier operation regimes, QCLs and QCL-based combs feature low spontaneous emission rates, small linewidth enhancement factors and narrow intrinsic linewidths (down to (sub)kilohertz levels^37,38^).

## Intracavity waveform dynamics

**Fig. 4:**
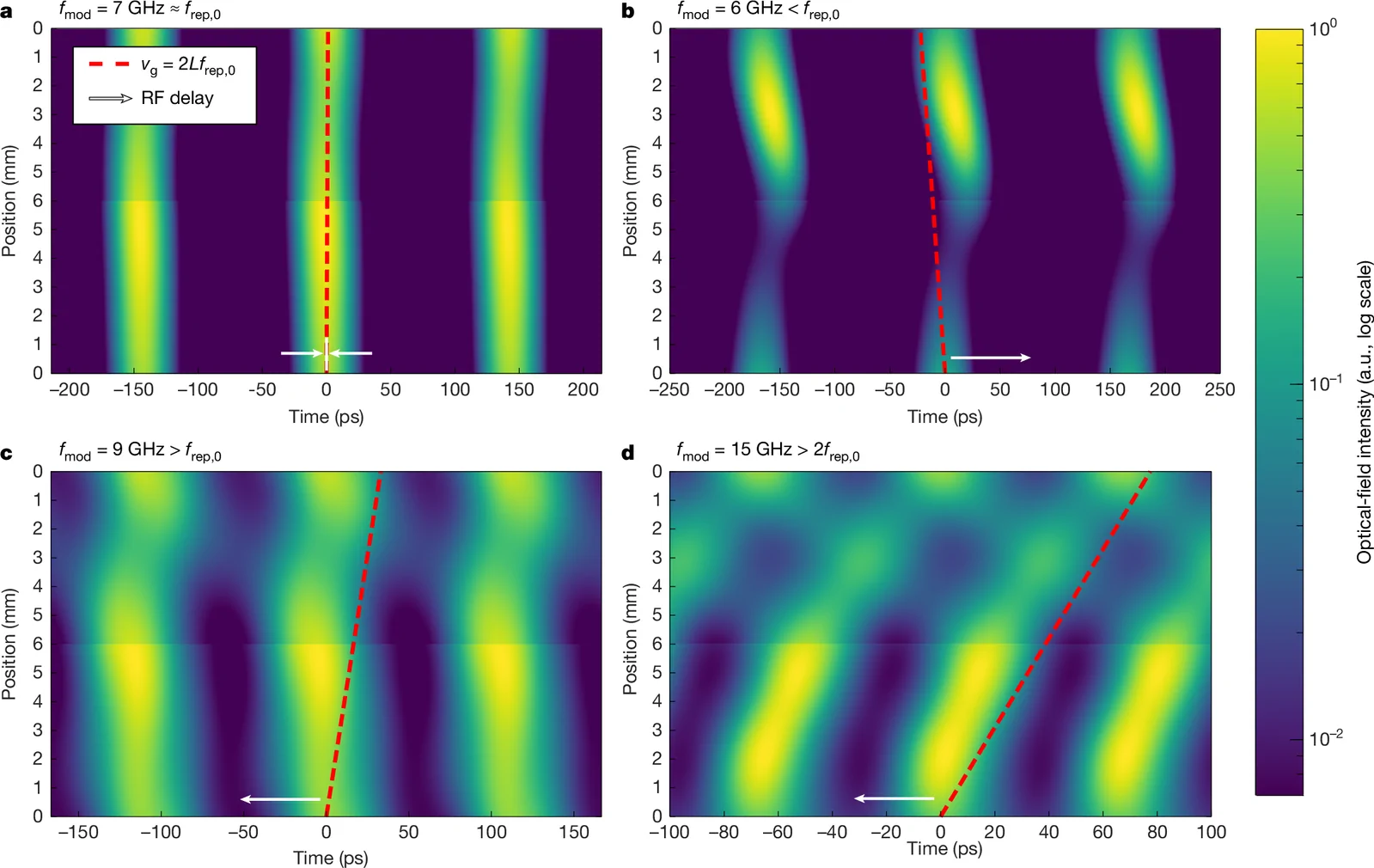
Simulated intracavity waveform dynamics. **a**–**d**, Comparison of the intracavity optical-field intensities for different modulation frequencies of 7 GHz ≈ *f*_rep,0_ (**a**), 6 GHz < *f*_rep,0_ (**b**), 9 GHz > *f*_rep,0_ (**c**) and 15 GHz > 2*f*_rep,0_ (**d**), showing three round-trip times of each periodic waveform in the unfolded cavity representation in the co-propagating reference frame. Microwaves are injected at the cavity coordinate of 0 mm. The dashed red lines show the reference optical group velocity under free-running or resonant modulation conditions (*f*_mod_ = *f*_rep,0_), and the white arrows indicate the delay (phase shift) between the maxima of the modulation RF wave and the optical pulse, showing the direction of the resulting pulse-pulling effect (see also Fig. 1c). Any deviation from the straight vertical direction in the individual panels indicates a local variation of the optical pulse group velocity. At the central horizontal line (cavity coordinate of 6 mm), the optical-field intensity is reduced owing to the finite reflectivity at the waveguide facet. The time-domain intensity profiles from Fig. 3d are taken at this point.

As the analytical model only gives round-trip averaged solutions, we use numerical simulations to study the intracavity waveform dynamics. In Fig. 4, we plot the intracavity optical-field intensities on a logarithmic-scale two-dimensional colour map using a co-propagating reference frame determined by the modulation frequency *f*_mod_. Three round trips are displayed in the unfolded cavity representation. A vertical line between the plot edges represents propagation at the new round-trip-averaged group velocity of *v*_g_ = 2*Lf*_mod_, and a pulse completes one round trip by traversing the entire plot range in the vertical direction. The red lines mark the reference group velocity in the resonant case (*f*_mod_ = *f*_rep,0_). The white arrows indicate the round-trip-averaged delay between the peak of the RF and optical wave, shown in Fig. 1c, marking the direction of the pulse-pulling effect. In Fig. 4a, we show the standard active mode-locking case, where the optical pulse propagates at the natural group velocity without any notable perturbations, and there is no delay between the RF and optical waves.

We study three distinct regimes within our experimental range. In Fig. 4b, the modulation frequency of 6 GHz is below *f*_rep,0_, and the intracavity waveform features a substantial change in its shape, amplitude and local group velocity. The white arrows indicate that the optical pulse is ahead of the modulation wave, experiencing a negative gain/loss slope, slowing down the pulse propagation, as is also apparent from the red line. There is considerable energy breathing, and reducing the modulation frequency to around *f*_rep,0_/2 leads to a gain-switched regime with broadband incoherent pulses. In Fig. 4c, where *f*_mod_ = 9 GHz > *f*_rep,0_, the average group velocity is faster than the natural one, with visible changes (oscillations) of the local group velocity throughout the round trip. The optical pulse lags behind the RF wave peak, experiencing a positive gain/loss slope, speeding up the pulse propagation.

For modulation frequencies close to and above 2*f*_rep,0_, as shown in Fig. 4d for *f*_mod_ = 15 GHz, pulses propagate along a diagonal instead of a vertical direction in the co-propagating frame. This indicates the formation of a double counterpropagating pulse structure, where a single pulse only needs to traverse half the cavity for the waveform to complete a full signal period. The propagation of each optical pulse itself is not much faster than the natural one (red line). With several intracavity pulses forming for modulation frequencies close to or above integer multiples *N* of the repetition rate *N* × *f*_rep,0_, akin to resonant harmonic mode-locking^24^, very high modulation frequencies should be possible without extreme (superluminal) group velocities, as the waveform is instead split into several pulses.

Detailed comb properties are in Extended Data Figs. 4–8 and Supplementary Information.

## Absorption spectroscopy

**Fig. 5:**
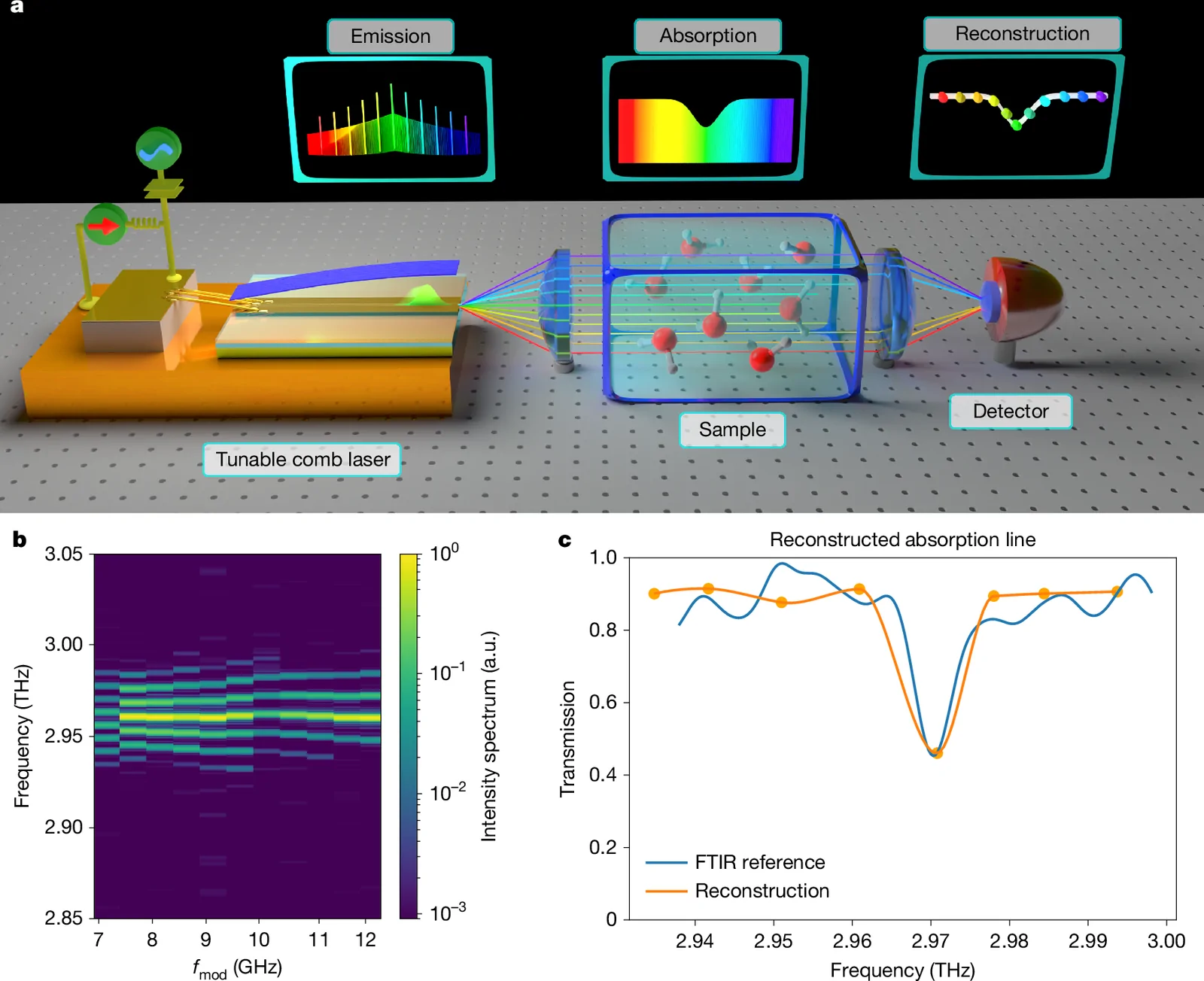
Absorption spectroscopy. **a**, Illustration of the experimental set-up, requiring only an intensity detector in combination with our tunable comb laser to investigate a sample under test. **b**, Emission spectrum of the tunable comb as a function of modulation frequency. **c**, The reconstructed absorption line (orange) shows good agreement with the reference FTIR spectrometer measurement (blue). Source Data

To demonstrate the potential use in practical applications, we developed an absorption spectroscopy experiment^39^. As illustrated in Fig. 5a, the experimental set-up consists of only a few elements. The tunable comb laser shines on a sample with absorption features within the emission spectrum. By tuning the RF modulation frequency, the emission spectrum is swept, and the detector intensity is recorded. The absorption spectrum is then reconstructed using an algorithm to solve the matrix system of equations **b** = **Mx**, where **b** contains the measured detector intensities, **M** is the comb laser emission spectra and **x** is the frequency-dependent absorption of the sample, each describing its dependence on the modulation and optical frequency. The absorption profile can be retrieved from a known laser source matrix **M** and the measured detector intensity as a function of the modulation frequency.

We used a tapered waveguide sample^23^, which allows sweeping only the RF modulation frequency, while all the other crucial parameters (d.c. laser bias, RF power, temperature) are stabilized at constant values. This produces tunable comb spectra with a relatively stable central emission frequency, as shown in Fig. 5b. The investigated sample is water vapour with absorption peaks in the THz spectral range. In Fig. 5c, we plot the reconstructed absorption line at 2.97 THz, and compare it with a standard reference measurement performed with a Fourier transform infrared (FTIR) spectrometer. The two methods show good agreement on all crucial aspects: the absorption resonance central frequency, width and depth. This highlights the applicability of the repetition-rate-tunable comb in spectroscopy, featuring a simple, compact experimental set-up.

## Conclusion

We have demonstrated, both in experiments and simulations, how a strong spatiotemporal gain modulation along the entire semiconductor laser cavity can sculpt the intracavity waveforms into coherent pulse trains with continuously tunable repetition rates in the time domain, and frequency combs with continuously tunable mode spacings in the frequency domain. Within the cavity, both slow and fast light^14^ with a continuously tunable group velocity and broad spectral coverage can be generated via a pulse-pulling effect. Besides discovering this regime, this also paves the way for many interesting applications. In the frequency domain, the tunable mode spacing could be used for high-resolution and dual-comb spectroscopy^18,19^ without spectral gaps. In the time domain, pulses can be synchronized to an arbitrary external microwave frequency reference. These tunable states are robust, stable, reproducible and fully determined by the microwave driving field and the laser bias.

We demonstrated a functionality similar to tunable external cavity lasers^40^, where the repetition rate is tuned with a movable mirror. However, those systems include complex set-ups with external components, slower tuning times, stringent alignment and additional noise. Our compact chip-scale device has no movable parts and features a fully electronic control, enabling a much faster repetition frequency tuning on the order of 1 μs. Although photonic circuits based on Mach–Zehnder interferometers can be integrated monolithically^41^, those enable only discrete repetition rate multiplication. Compared with non-resonant electro-optic combs^42^ that also display wide repetition-rate tunability, our device features a compact, fully monolithic configuration and relatively wide spectral coverage without cascaded modulators and/or nonlinear optical elements (fibres, waveguides). In summary, compared with other commonly used frequency comb systems (actively and passively mode-locked lasers^13,43^, external cavity laser combs^44^, electro-optic combs^42^, fibre laser combs^45^, Kerr microcombs^46^, optical parametric oscillators^47^ and so on), our device simultaneously combines the continuously and widely tunable repetition rate without any spectral gaps on a simple monolithic semiconductor laser cavity, without any additional components or external parts, constituting a robust and reliable system with a compact on-chip footprint and straightforward operation by tuning only one parameter—the RF modulation frequency.

The crucial feature is an efficient spatiotemporal gain modulation across the entire laser cavity, which itself supports broadband operation with many longitudinal modes. Both conditions can be fulfilled by Fabry–Pérot semiconductor lasers: although we demonstrated this with THz QCLs (exceptionally suitable for low-loss microwave propagation), expanding this concept to other types of semiconductor lasers and wavelength regions should be relatively straightforward. Even with large microwave losses, a segmented metallic structure could be defined along the side of the semiconductor laser waveguide, inducing a custom phase-shifted modulation function without the requirement of microwave propagation. Another feature of THz QCLs is their relatively short gain recovery time, affecting the laser dynamics. Nevertheless, this is also not a limiting factor, as lasers with longer gain recovery times have already been investigated in the theoretical study in ref. ^17^, where in the linear stability analysis, no limitations for the detuning frequency were found.

Considering these aspects, the approach presented in this work will be applicable to various semiconductor laser devices across the entire infrared and visible spectral regions^48–50^ with a vast range of applications in sensing and telecommunications.

## Methods

### Sample fabrication, mounting and basic properties

The laser chips were fabricated from wafers grown by molecular beam epitaxy (MBE) of the GaAs/AlGaAs active material from ref. ^27^, following the standard planarized waveguide fabrication process. The main steps include cleaving a smaller sample (typical dimension of (9 × 10) mm^2^) and an n-doped GaAs piece with larger dimensions of (14 × 16) mm^2^. Both pieces are metallized using an electron beam evaporation machine, with a material stack of Ta/Cu/Ti/Au (5 nm/250 nm/50 nm/500 nm). Subsequently, these pieces were attached together with thermocompression bonding by applying a pressure of 5 MPa for 15 minutes at a temperature of 320 °C in a vacuum. After mechanical and wet etch (3:1 ratio solution of citric acid and hydrogen peroxide at 35° C) thinning steps, the active waveguide top metallization was defined using standard photolithography, tilted evaporation and lift-off techniques, with a stack of Ta/Cu/Ta/Au (5 nm/150 nm/50 nm/500 nm), with a typical top width of 40 μm. The active region waveguides were subsequently defined using SiCl_4_/N_2_-based inductively coupled plasma etching, where the top gold serves as an etch hardmask. An anisotropic dry etching produces vertical and smooth sidewalls. After coating the sample with an adhesion promoter (AP3000), a thick layer of BCB polymer was deposited via several spin-coating and oven-curing steps. The thick BCB layer (about 27 μm) was then thinned down precisely to match the height of the active region waveguide (10 μm) with reactive ion etching, using an O_2_/CF_4_ chemistry. After a successful planarization, an extended top metallization with a typical width of 300 μm was defined using the stack Ti/Au (15 nm/500 nm), centred over the active ridge waveguides. The backside of the bonding substrate was mechanically thinned to a typical thickness of 200 μm for improved thermal dissipation to the bottom heat sink during laser operation. Finally, the entire backside surface was metallized with Ti/Au (10 nm/100 nm).

**Extended Data Fig. 1:**
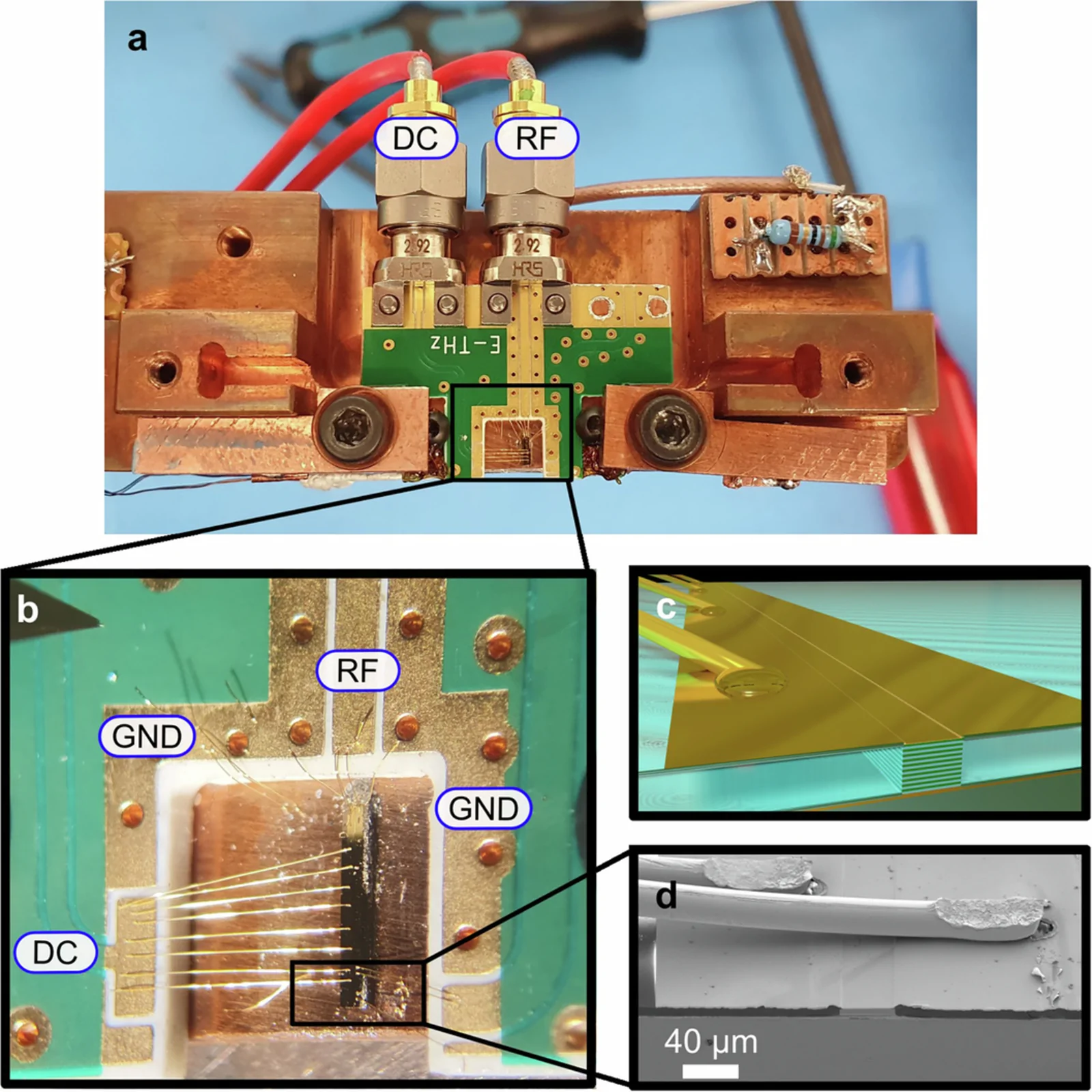
Images of the laser sample. In **a**, we show a photograph of the cryostat cold finger, where the laser sample is mounted using a custom RF PCB, optimized for RF readout and injection experiments. **b** Optical microscope image of the laser sample, which is soldered to a copper submount. The DC laser bias wires are spread along the entire waveguide from the side. The RF modulation is facilitated via a set of short wire bonds, connecting the feeding low-loss RF coplanar waveguide with the back side of the planarized THz QCL device. Ground wires are added to connect the PCB, laser ground plane, and the copper submount. **c**, **d** The inset images show a 3D illustration and an SEM image of the device, displaying the front side of a planarized waveguide with the active region, BCB polymer, and extended metallization. DC wire bonds are visible on the top metal layer.

**Extended Data Fig. 2:**
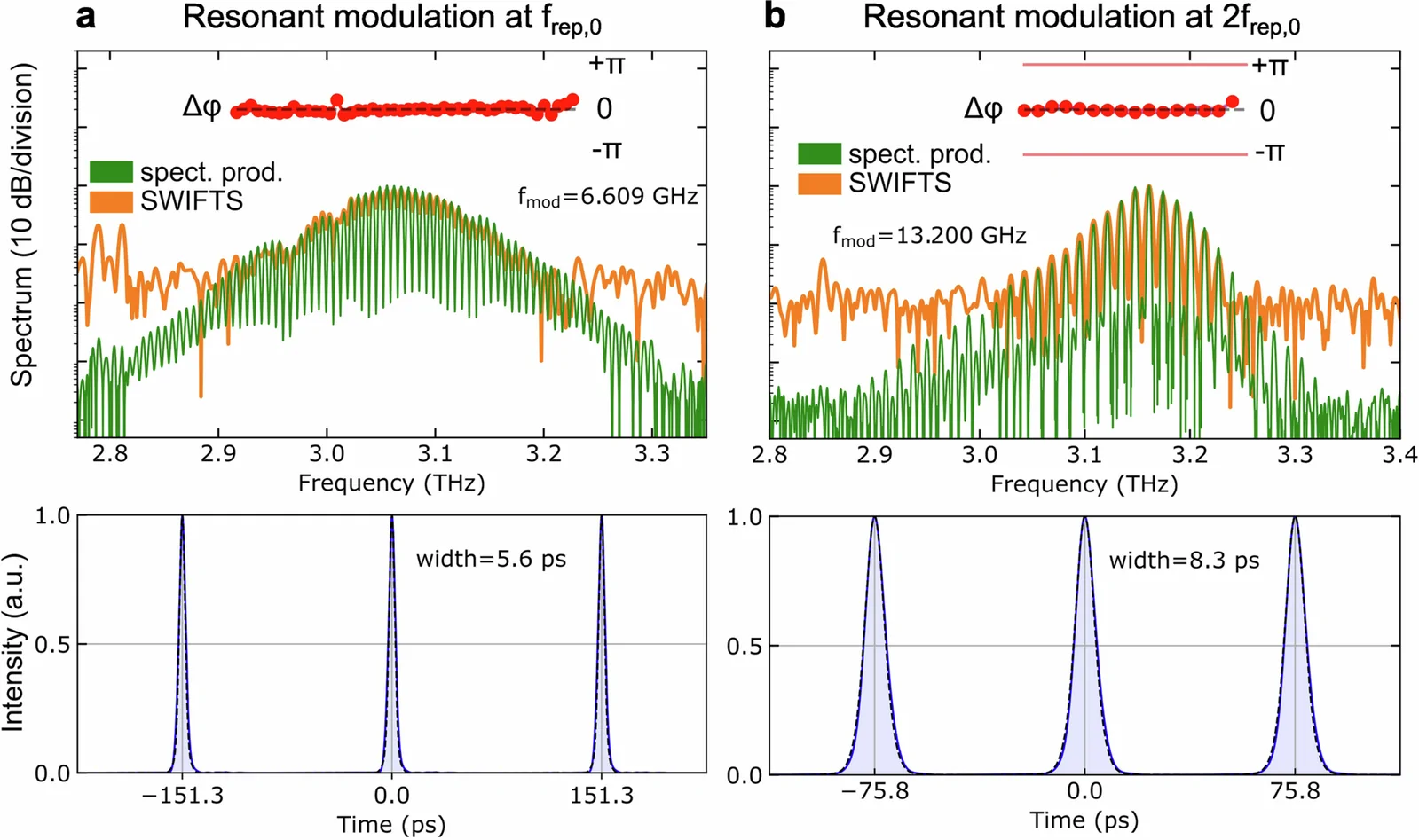
Experimental SWIFT spectroscopy measurement results under resonant conditions. Microwave modulation at a frequency of ${f}_{{\rm{mod}}}=\,{f}_{{\rm{rep}},0}$ in **a**, and 2*f*_rep,0_ in **b**, results in the broadest spectral bandwidths and the shortest pulses, as the system operates in the standard active mode-locking regime, i.e., without any substantial detuning between the modulation and natural repetition frequency (or its integer harmonics). These results are from the same planarized ridge sample as investigated in the Main Text Figs. 3 and 4. More details can be found in the Supplementary Information, section S1.

**Extended Data Fig. 3:**
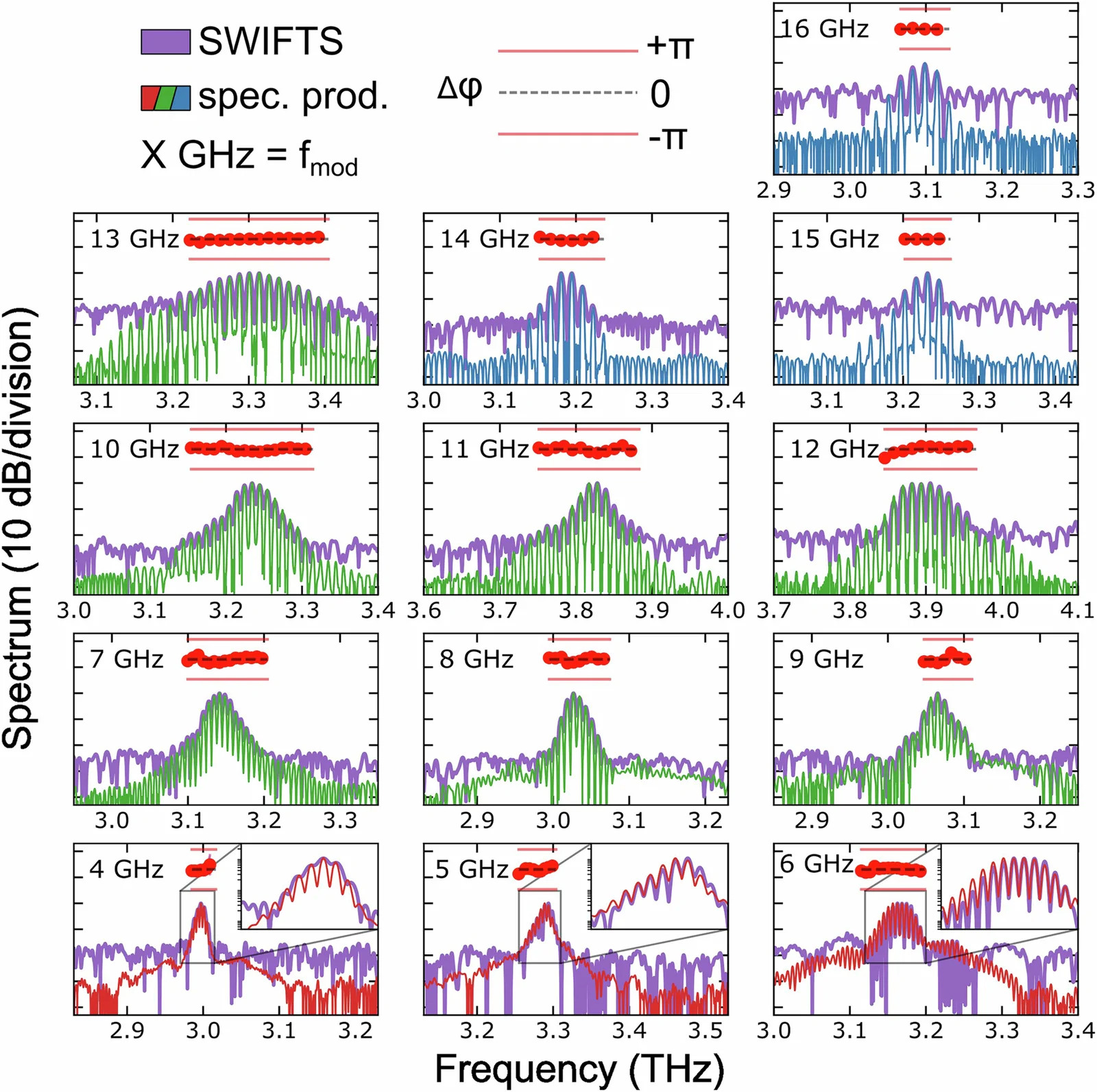
Detailed SWIFT spectroscopy results for each frequency point between 4-16 GHz. In each panel, the modulation frequency, the SWIFT spectrum, the spectrum product, and the extracted intermodal phase differences are displayed. This is the same set of measurements as shown in the Main Text Fig. 3, for the planarized ridge sample. The color codes are related to the frequency detuning: red stands for ${f}_{{\rm{mod}}} < \,{f}_{{\rm{rep}},0}$, green for ${f}_{{\rm{rep}},0} < \,{f}_{{\rm{mod}}} < \,2{f}_{{\rm{rep}},0}$, and blue for ${f}_{{\rm{m}}{\rm{o}}{\rm{d}}} > \,2{f}_{{\rm{r}}{\rm{e}}{\rm{p}},0}$. In this case, the central emission frequency is changing for different modulation frequencies, as the DC laser bias and RF power are adjusted for each point (see also Extended Data Table 2). However, with the tapered sample that was used in the absorption spectroscopy experiment in Main Text Fig. 5, all these parameters can be stabilized, resulting in a very stable central emission frequency and straightforward use in applications. More details about the SWIFT spectroscopy results can be found in the Supplementary Information, section S2.

**Extended Data Fig. 4:**
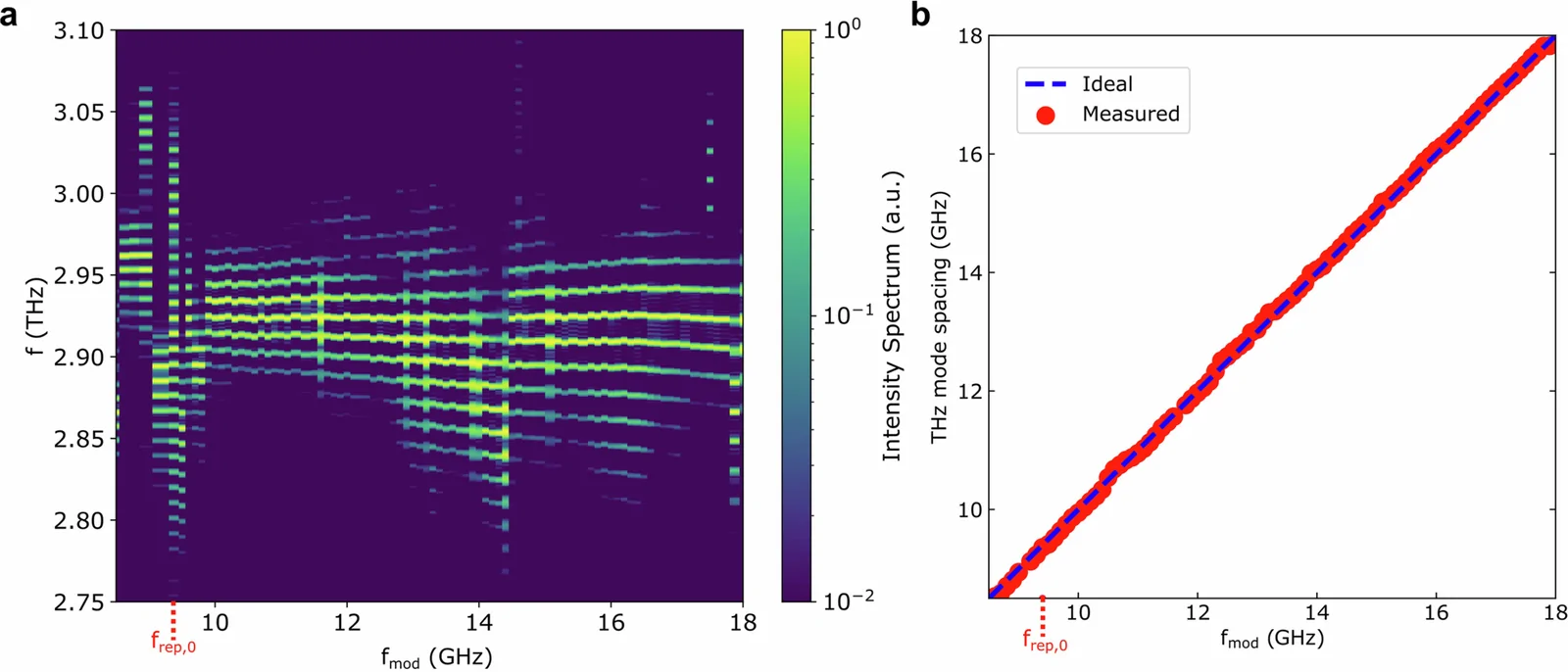
Experimental modulation frequency sweep study for another tapered waveguide device. **a** Measured THz emission spectra with a relatively stable central emission fruency, **b** extracted mode spacing as a function of the injection frequency for a 4.2 mm long planarized tapered waveguide device, displaying a continuous tuning of the mode spacing that matches the modulation frequency. Here, a modulation frequency step of 100 MHz was used, while all the other parameters (DC laser bias, heatsink temperature, RF power) were stablized at constant values.

**Extended Data Fig. 5:**
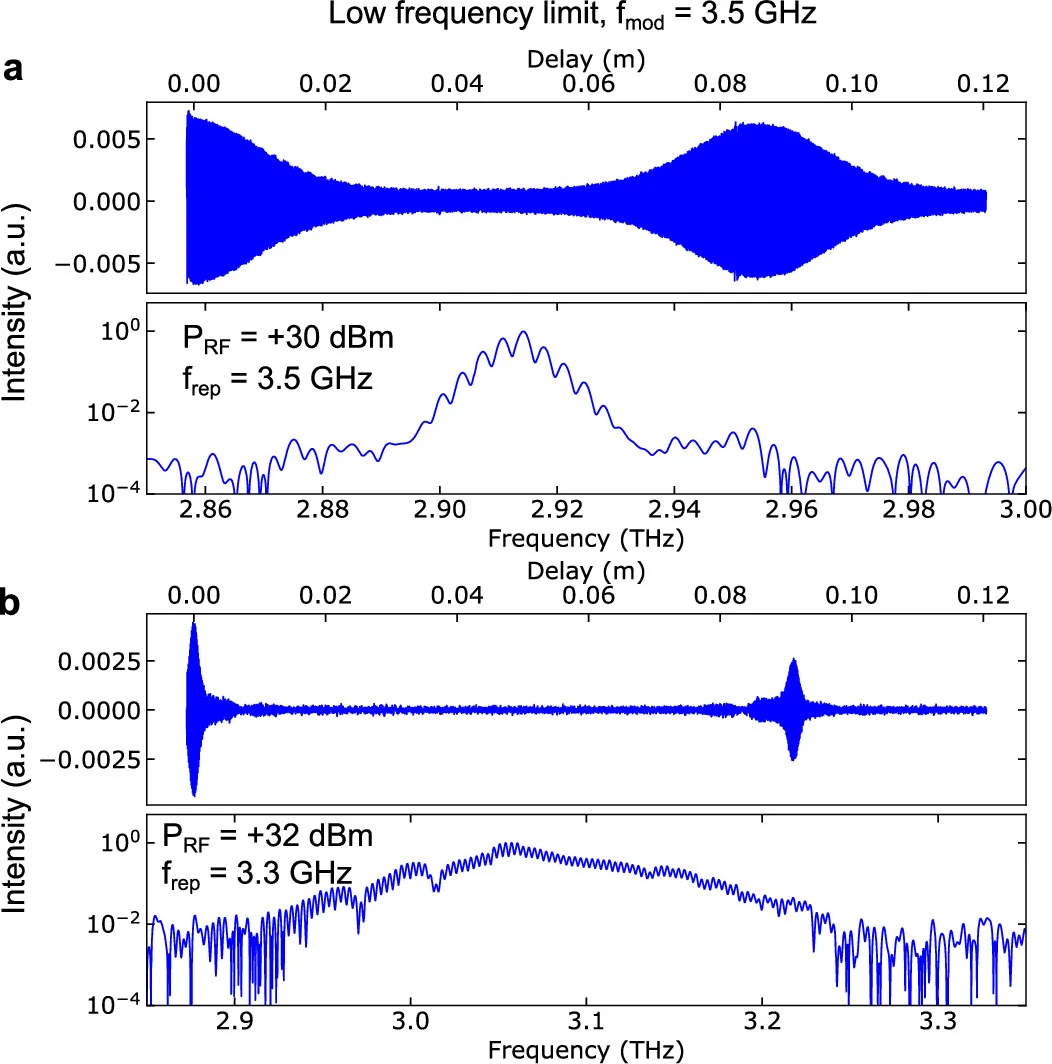
Measurement results at the experimentally-observed lower modulation frequency limit of 3.5 GHz. In the top and bottom panels, we show the measured FTIR interferograms and emission spectra, respectively. At the low-frequency limit, there are two competing operation regimes for the same modulation frequency, which depend on the RF modulation power. In **a**, we display the tunable coherent pulse state and in **b** the broadband, low-coherence gain-switched state with a mode spacing of *f*_rep,0_/2, obtained for increased modulation powers (P_RF_≥+32 dBm). In the latter case, the amplitude and duration of the negative RF half-cycle are so large that the pulse gets fully attenuated (absorbed), i.e., it is energetically more favorable for the laser to enter a gain-switched regime, where incoherent pulses are propagating at the normal group velocity, but are fully absorbed between each modulation period. A more detailed discussion on this is in the Supplementary Information, section S6.

**Extended Data Fig. 6:**
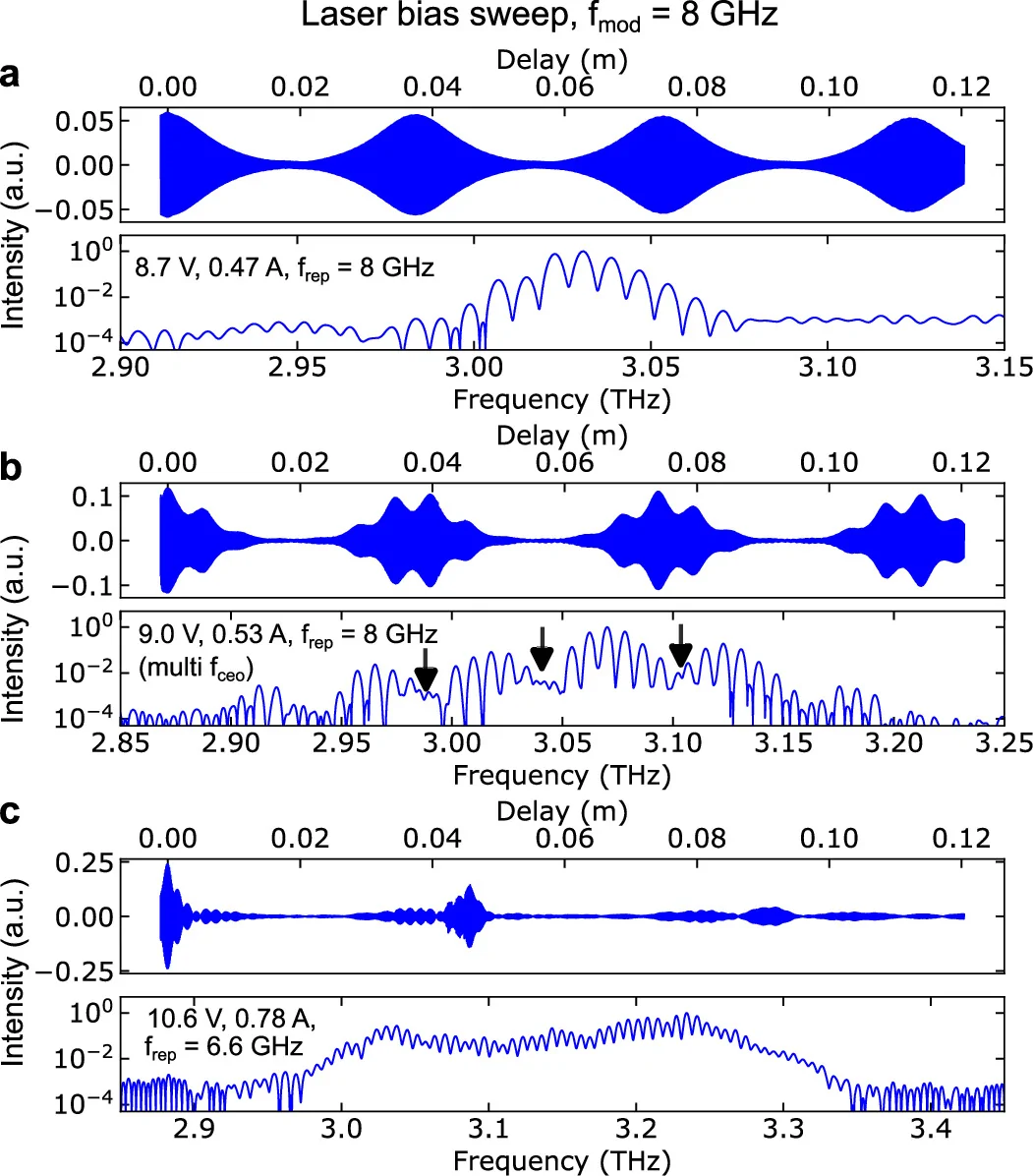
Experimental laser bias sweep for a modulation frequency of 8 GHz. Here, we highlight three different operating regimes that depend on the DC laser bias, showing the FTIR interferograms and emission spectra in the top and bottom panels, respectively. **a** For a relatively low DC bias close to the lasing threshold, the coherent tunable pulse state is generated. **b** Increasing the bias further, multi *f*_ceo_ sub-combs are formed with visible spectral stitching points, as indicated with the arrows. **c** For a very high bias (close to the maximum), broadband incoherent spectra are generated with the natural mode spacing, imposed by the cavity length. More details can be found in the Supplementary Information, section S7.

**Extended Data Fig. 7:**
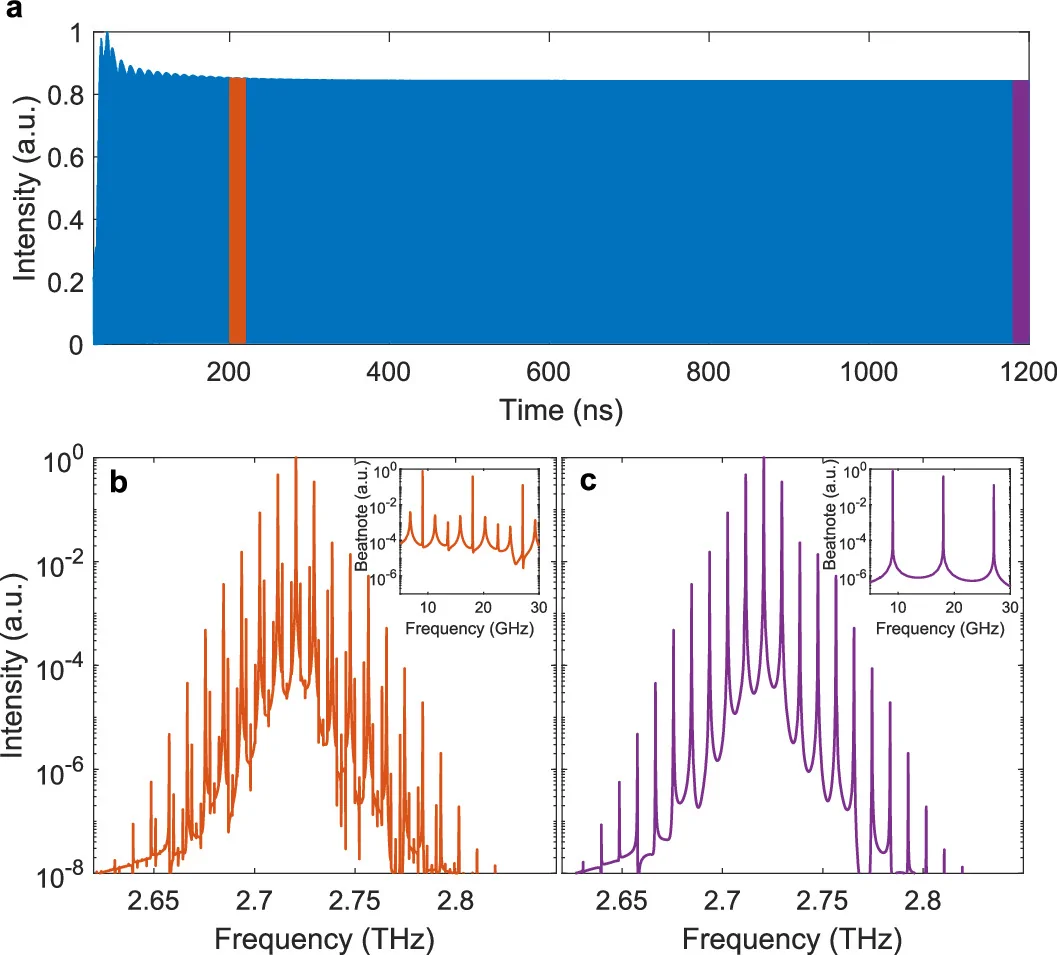
Numerical simulation results of the temporal build-up dynamics of the tunable frequency comb. **a** The out-coupled intensity over time, displaying a fully stabilized coherent state in less than 1000 ns. **b** Intensity spectrum recorded after 200 ns still shows some noisy sidemodes. **c** Intensity spectrum recorded after 1180 ns displays a fully stabilized tunable comb state with no additional sidebands in the spectrum. The insets show the RF beatnote signals of the respective emission spectra. More details, including a study on the dependence of the detuning frequency on the build-up dynamics, can be found in the Supplementary Information, section S10.1.

**Extended Data Fig. 8:**
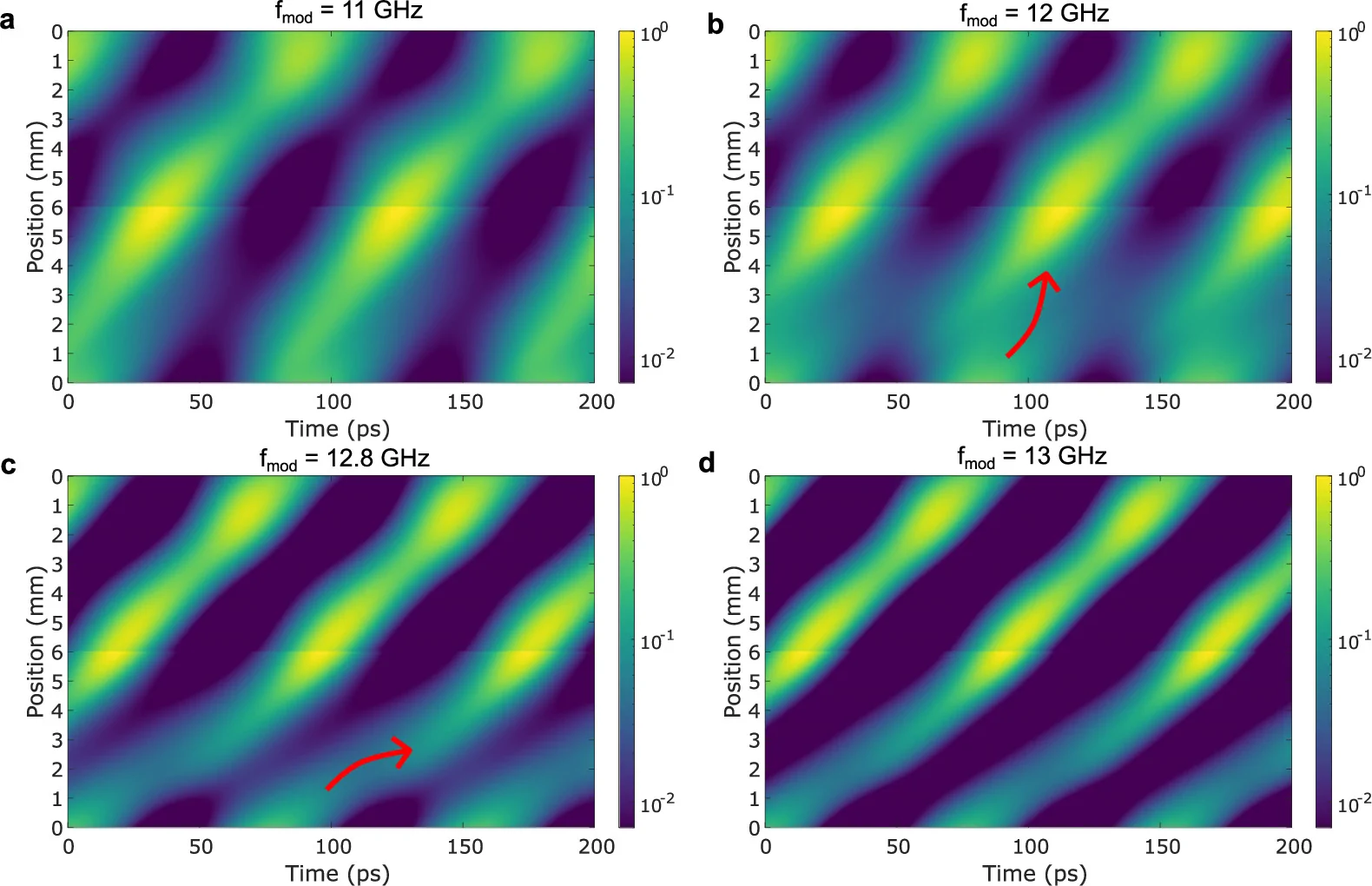
Numerical simulations of the transition between the single and double pulse regimes. We display the intracavity field dynamics for modulation frequencies close to the second harmonic of the natural repetition rate, using the laboratory reference frame. The red arrows indicate that the switch between a single to a double intracavity pulse happens already below the second harmonic. At the output, there is always a coherent comb state with a repetition rate that exactly matches the modulation frequency, i.e., there is no discontinuity at the output due to an internal transition between a different number of pulses within the cavity. More details are in the Supplementary Information, section S10.2.

The fabricated samples were then cleaved into smaller chips and soldered to a copper submount using indium paste and die bonding. The electrical connection was established with bonding wires. A crucial component for efficient microwave modulation is a custom RF-optimized printed circuit board (PCB), shown in Extended Data Fig. 1, which separates the RF and d.c. laser bias. The two-layer PCB was fabricated on a Rogers 4350 substrate with a thickness of 0.8 mm and features a low-loss coplanar waveguide for RF readout and injection experiments. The PCB was screwed to the copper submount and mounted on a cryostat cold finger. Using a standard RF connector (HK-LR-SR2) and low-loss semi-rigid RF cables (SUCOFORM 86 FEP), the electrical connections were accessible from the outside of the cryostat. A possible further improvement would be using a touch down RF probe (G-S-G) and optimizing the design of the waveguide extended top metallization to be matched to 50 Ω (we discussed the possibility of microwave and impedance design of planarized waveguides in more detail in ref. ^7^).

Typical planarized THz QCLs with an active waveguide width of 40 μm, height of 10 μm and extended top metallization width of 300 μm feature cleaved end facet reflectivities of around 70% at 3 THz, waveguide losses of around 15–20 cm^−1^, a group velocity dispersion of around 5 × 10^4^ fs^2^ mm^−1^ at 3 THz, a third-order (Kerr) nonlinearity with a nonlinear refractive index of around *n*_2_ = 1.8 × 10^−14^ m^2^ W^−1^ at THz frequencies, and a low impedance of around 5 Ω at 20 GHz. Further details on the fabrication process, the optical, RF and thermal properties, and the simulation and measurement results of planarized THz QCLs of the same active material can be found in ref. ^7^, which is entirely dedicated to the planarized waveguide platform.

### Experimental methods

For all the measurements, the sample was mounted on a cold finger cryostat and placed inside a sealed cryostat shield under low-vacuum conditions (down to 10^−6^ mbar) and cooled using a liquid helium transfer line, with typical operating temperatures between 20 K and 80 K. The heat-sink temperature was stabilized using a Lakeshore temperature controller with a tunable proportional–integral–derivative (PID) loop. The low vacuum and low temperatures are to ensure the best possible sample performance (minimize atmospheric absorption and maximize output power), and are not an inherent limitation. We have previously demonstrated frequency comb operation up to 115 K in continuous-wave mode with a tapered device in ref. ^23^, and THz QCLs are in general getting close to room-temperature operation (see also ref. ^51^; narrowband, pulsed mode operation at *T*_max_ = 261 K = −12 °C). Broadband THz QCLs are also expected to increase the operating temperatures, and this concept is applicable also to other semiconductor lasers that work at room temperature. Therefore, we expect such tunable mode-locked devices to operate also at ambient conditions, which would make them widely applicable also outside the lab.

The d.c. laser bias was stabilized with a Keithley 2420 source operated in voltage-controlled mode. The additional microwave modulation was achieved using a Rohde&Schwarz SMB 100A microwave signal generator in combination with one of the following microwave amplifiers: Pasternack PE15A5017, Mini-Circuits ZVE-3W-183+ or Fairview Microwave FMAM5123, with each amplifier covering a different frequency and/or power range.

The laser emission spectra were obtained using an FTIR spectrometer (Bruker Vertex 80v). Based on a Michelson interferometer, the input beam is split using a 50:50 mylar beam splitter into two arms (one with a movable mirror), with the two beams recombined on a room-temperature deuterated triglycine sulfate detector. The FTIR spectrometer was evacuated to avoid any frequency-dependent THz absorption in the optical beam path.

With SWIFT spectroscopy measurements^29,33^, the optical beatnote generated between adjacent lasing modes was measured with a fast detector (bandwidth >30 GHz) after passing through an FTIR spectrometer^52^. The latter acts as a tunable frequency-selective filter, while a room-temperature Schottky diode^53^ was used as a fast detector. Placed at the output of the FTIR spectrometer and connected to an RF spectrum analyser (Rohde&Schwarz FSW67) with an in-phase and quadrature (IQ) demodulator function, the optical beatnote signal was measured. This method allows to verify the coherence of the measured emission spectra, obtain the intermodal phases and reconstruct the time-domain output intensity. A detailed description and illustration of the experimental set-up can be found in ref. ^7^.

For the absorption spectroscopy experiments, a room-temperature pyroelectric detector (Gentec-EO: THZ2I-BL-BNC) was used in combination with a mechanical chopper at a frequency of 30 Hz.

### Numerical simulation model

The QCL is modelled based on the widely used Maxwell-density matrix equations capturing the coupled optical and quantum dynamics^54^, which are here supplemented by transmission line equations for describing the propagation of the microwave driving signal^9^. This approach has been found to be well suited for the quantitative modelling of actively mode-locked QCLs^9^. As usual, the slowly varying envelope approximation and rotating wave approximation are invoked to reduce the numerical load^54^. In detail, starting from Maxwell’s equations and using the slowly varying envelope approximation, the cavity-field dynamics are described by optical propagation equations of the form^54^

3 $${v}_{\mathrm{g,0}}^{-1}\,{\partial }_{t}{E}^{\pm }=\mp {\partial }_{x}{E}^{\pm }+{p}^{\pm }-\frac{a}{2}{E}^{\pm }-{\rm{i}}\frac{{\beta }_{2}}{2}{\partial }_{t}^{2}{E}^{\pm }.$$

*E*^±^(*x*, *t*) represents the complex amplitude of the forward- and the backward-travelling electric-field components, respectively. Furthermore, *x* and *t* denote the propagation coordinate along the waveguide cavity and the time. The optical waveguide properties are described by the group velocity *v*_g,0_, power loss coefficient *a* and background group velocity dispersion coefficient *β*_2_. The field boundary conditions at the left (*x* = 0) and right (*x* = *L*) waveguide facet are and ${E}^{-}(L,t)={r}_{2}{E}^{+}(L,t)$, where *r*_1,2_ denote the corresponding field reflection coefficients. Equation (3) is numerically solved using the well-established Risken–Nummedal method^54,55^. The polarization term *p*^±^(*x*, *t*) is directly computed from the density matrix *ρ*(*x*, *t*), capturing the quantum active region dynamics. The density matrix evolution is described by a Lindblad-type equation for a representative QCL period

4 $${\partial }_{t}\rho =-\frac{{\rm{i}}}{\hbar }[H,\rho ]+{\mathcal{D}}(\rho ),$$

with the reduced Planck constant *ħ*, supplemented by suitable periodic boundary conditions for the levels^9^. We go beyond the usual two-level approximation by considering all five relevant levels of the modelled active region^27^, that is, *ρ*(*x*, *t*) and the system Hamiltonian *H*(*x*, *t*) are here square matrices of order five. In addition to the relevant optical transitions, *H* also includes tunnelling across the injection barrier. Scattering-induced transport and dephasing are accounted for by the Lindblad-type dissipation term ${\mathcal{D}}(\rho )$. The rotating wave approximation is invoked for simulating equation (4) as discussed above, and the resulting equation system is numerically solved by an explicit third-order Adams–Bashforth scheme, combining excellent efficiency with good stability. For obtaining a self-consistent model, carrier transport simulations are used to extract the Hamiltonian matrix elements as well as scattering and dephasing rates, where their dependence on the microwave modulation voltage *u*_*Δ*_(*x*, *t*) is captured by a linear expansion around the operating point^9^. Neglecting the influence of the photon-induced current on *u*_*Δ*_, which is justified for strong modulation close to lasing threshold, the transmission line equations can be analytically solved, yielding^9^

5 $${u}_{\varDelta }(x,t)={u}_{{\rm{m}}}\Re \left\{\frac{\cos [{\beta }_{{\rm{m}}}(x-L)]}{\cos ({\beta }_{{\rm{m}}}L)}\exp (-{\rm{i}}{\omega }_{{\rm{m}}}t)\right\}.$$

Here *u*_m_ is the applied modulation amplitude at *x* = 0, *ω*_m_ denotes the (angular) modulation frequency, and the associated propagation constant is obtained from the effective microwave index *n*_m_ and damping coefficient *a*_m_ as *β*_m_ = *n*_m_*ω*_m_/*c* + i*a*_m_, with the vacuum speed of light *c*.

### Analytical model of detuning mechanism

The stability of active mode-locking for frequency-detuned gain modulation has been analytically investigated with a Haus-type master equation approach^11,17^. Within this model, a stable Gaussian pulse solution exists for arbitrary detuning^17^. As discussed in the main text, the underlying reason is that for increased modulation frequency, the travelling gain/loss window moves at a higher velocity, thus an intracavity pulse initially propagating with the natural group velocity will experience more gain at its leading edge than its trailing edge and be pulled forward^17^. For decreased modulation frequency, the opposite effect takes place. In the following, we demonstrate that the resulting modification of the optical group velocity is also the enabling mechanism for the widely tunable repetition rate observed in this paper.

As the Haus theory describes the optical pulse by its round-trip-averaged envelope, this approach is not valid if the pulse duration changes excessively during a round trip. Thus, the theoretical framework may not be valid for usual semiconductor laser geometries where only a small waveguide section is modulated. However, for our case where the optical pulse co-propagates with the modulation signal, this assumption is more reasonable. Specifically, as can be seen from Fig. 4, the propagation of the forward- and backward-propagating pulses can in good approximation be described by constant, albeit different group velocities ${v}_{{\rm{m}}}^{\pm }$. The resulting optical round-trip time $L/{v}_{{\rm{m}}}^{+}+L/{v}_{{\rm{m}}}^{-}$ then equals the inverse of the microwave modulation frequency ${f}_{\mathrm{mod}}={\omega }_{{\rm{m}}}/(2{\rm{\pi }})$. As can be seen from Fig. 4d, for modulation frequencies in the vicinity of twice the natural pulse repetition rate, the operation switches to a double pulse (*N* = 2). Thus, we arrive at the more general condition

6 $$L/{v}_{{\rm{m}}}^{+}+L/{v}_{{\rm{m}}}^{-}\,=\,N/{f}_{\mathrm{mod}}.$$

We introduce a reference frame with the retarded time coordinate $\tau =t\mp x/{v}_{{\rm{m}}}^{\pm }$, co-moving with the altered optical group velocity. From equation (3), we obtain equation (1) with the detuning parameter ${\delta }^{\pm }={({v}_{{\rm{m}}}^{\pm })}^{-1}-{v}_{\mathrm{g,0}}^{-1}$. As in ref. ^17^, we neglect chromatic dispersion in equation (1), and describe the round-trip-averaged polarization term by a classical gain as ${p}^{\pm }=({g}^{\pm }+{g}_{\omega }{\partial }_{\tau }^{2}){E}^{\pm }/2$. Here *g*^±^(*τ*) denotes the saturated time-modulated gain and *g*_*ω*_ characterizes the spectral gain bandwidth in parabolic approximation. Likewise, the loss coefficient *a* in equation (1) is now round-trip-averaged, thus also containing the contribution of mirror outcoupling $-{\rm{ln}}({r}_{1}{r}_{2})/L$. A stationary analytical solution can be obtained from equation (1) by inserting a Gaussian ansatz ${E}^{\pm }\propto \exp [-2{\tau }^{2}{({T}^{\pm })}^{-2}\,{\rm{ln}}\,2]$ with the full-width at half-maximum intensity pulse duration *T*^±^, and Taylor-expanding the gain around the pulse maximum as ${g}^{\pm }\approx {g}_{0}^{\pm }+{g}_{1}^{\pm }\tau +{g}_{2}^{\pm }{\tau }^{2}/2$. Equating the coefficients of terms $\propto \tau \exp [-2{\tau }^{2}{({T}^{\pm })}^{-2}\,{\rm{ln}}\,2]$, we see that a gain slope ${g}_{1}^{\pm }$ results in a detuning given by equation (2). We evaluate equation (2) for different scenarios to show that the pulse pulling due to the gain slope completely explains the observed synchronization of the pulse repetition rate to the microwave modulation. In contrast to ref. ^17^ where the gain is analytically determined under the assumption of slow gain recovery, we extract *g*(*τ*) directly from our simulations, thus capturing the full gain dynamics. In Fig. 1c, the simulated intensity pulse profile and net gain, obtained by averaging over the forward propagation cycle, are shown for different modulation frequencies. In Extended Data Table 1, we list the gain slope, pulse duration and group velocity extracted from the simulation results partially shown in Fig. 4. All in all, good agreement is found between the detuning parameter obtained from the simulated velocities ${v}_{{\rm{m}}}^{\pm }$ in Extended Data Table 1 and *δ*^±^ calculated from equation (2) with the listed values of *T*^±^ and ${g}_{1}^{\pm }$. The deviation is at most 21% for *δ*^±^ at 12 GHz and only a few per cent in most other cases, and can largely be attributed to the assumption of a stationary Gaussian pulse shape and constant ${v}_{{\rm{m}}}^{\pm }$ underlying equation (2). Especially for the 12-GHz case, which approaches the transition to the two-pulse regime, the assumption of constant ${v}_{{\rm{m}}}^{\pm }$ in our model is not as good as for the other cases. To summarize, the comparison between the simulation results and the analytical model clearly indicates that the pulse-pulling effect described above is the enabling mechanism for the widely tunable pulse repetition rate demonstrated in this paper.

### Absorption spectroscopy

The idea behind absorption spectroscopy is to obtain the absorption profile of an unknown sample by shining a known light source on it and then analysing the transmitted light to determine the origin of the specimen.

- FTIR. Interferograms of the transmitted light are measured using a Michelson interferometer, and transmitted spectra are retrieved via Fourier transform. Drawbacks: instrument size increases with resolution and the measurement is relatively slow.
- External cavity laser. A tunable single-mode laser source is directed at the sample. For each emission frequency, the transmission is evaluated and the signature absorption profile is automatically retrieved. Drawbacks: external mechanical components are involved and the emission frequency tunability range is not always wide enough.
- Tunable comb laser. Tunable, fast and compact. Drawbacks: no direct derivation of the absorption profile; reconstruction is required.

For the latter two sources, the intensity emission spectra of the laser can be described using a matrix

7 $${\bf{M}}=\left[\begin{array}{cccc}{I}_{0}({p}_{1},{\nu }_{1}) & {I}_{0}({p}_{1},{\nu }_{2}) & \cdots  & {I}_{0}({p}_{1},{\nu }_{n})\\ {I}_{0}({p}_{2},{\nu }_{1}) & {I}_{0}({p}_{2},{\nu }_{2}) & \cdots  & {I}_{0}({p}_{2},{\nu }_{n})\\ \vdots  & \vdots  & \ddots  & \vdots \\ {I}_{0}({p}_{m},{\nu }_{1}) & {I}_{0}({p}_{m},{\nu }_{2}) & \cdots  & {I}_{0}({p}_{m},{\nu }_{n})\end{array}\right].$$

Let *p*_*m*_ be the tuned parameter (in our case, the RF modulation frequency), and *ν*_*n*_ the laser emission frequency. Each element corresponds to the intensity at a specific tuning point and emission frequency, where the matrix **M** has dimensions of *m* × *n*.

- External cavity laser. The matrix is diagonal—at each tuning point (*p*_*m*_), only one specific frequency (*ν*_*n*_) is emitted. A well-conditioned matrix (with independent rows) results in stable and reliable numerical solutions.
- Tunable comb laser. The matrix is no longer diagonal as the source is multimode: at different tuning points, multiple frequencies are emitted. This leads to high collinearity (multiple lines carrying similar spectral information), causing ambiguity and instability in the reconstruction. An ill-conditioned matrix may result in unstable or inaccurate solutions owing to high sensitivity to smallperturbations.

The frequency-dependent absorption of the unknown sample can be represented as a column vector of size *n* × 1, where each entry corresponds to the absorption coefficient at a specific frequency

8 $${\bf{x}}=[\begin{array}{c}1{0}^{-A({\nu }_{1})}\\ 1{0}^{-A({\nu }_{2})}\\ \vdots \\ 1{0}^{-A({\nu }_{n})}\end{array}],$$

where *A* is the absorption vector of the sample as a function of the THz frequency. If the transmitted light intensity is measured as a function of the laser tunability, it is represented as a column vector of size *m* × 1,

9 $${\bf{b}}=[\begin{array}{c}S({p}_{1})\\ S({p}_{2})\\ \vdots \\ S({p}_{m})\end{array}]$$

where *S* is the integrated transmitted intensity as a function of the tuning parameter (in our case, the microwave modulation frequency). This integrated intensity **b** can be modelled as the matrix product **b = Mx**

10 $${\bf{b}}=\left[\begin{array}{c}{I}_{0}({p}_{1},{\nu }_{1})\,1{0}^{-A({\nu }_{1})}+{I}_{0}({p}_{1},{\nu }_{2})\,1{0}^{-A({\nu }_{2})}+\cdots +{I}_{0}({p}_{1},{\nu }_{n})\,1{0}^{-A({\nu }_{n})}\\ {I}_{0}({p}_{2},{\nu }_{1})\,1{0}^{-A({\nu }_{1})}+{I}_{0}({p}_{2},{\nu }_{2})\,1{0}^{-A({\nu }_{2})}+\cdots +{I}_{0}({p}_{2},{\nu }_{n})\,1{0}^{-A({\nu }_{n})}\\ \vdots \\ {I}_{0}({p}_{m},{\nu }_{1})\,1{0}^{-A({\nu }_{1})}+{I}_{0}({p}_{m},{\nu }_{2})\,1{0}^{-A({\nu }_{2})}+\cdots +{I}_{0}({p}_{m},{\nu }_{n})\,1{0}^{-A({\nu }_{n})}\end{array}\right].$$

Therefore, the absorption profile **x** can be retrieved by knowing the matrix **M** (for example, from prior FTIR characterization) and collecting the integrated transmitted intensity through the sample with an appropriate detector. This general concept applies to any laser source, provided **M** is known and reproducible, allowing mathematical reconstruction of the sample’s absorption profile **x** from an integrated intensity measurement **b**.

- For the external cavity laser, as **M** is diagonal, inversion is straightforward and the absorption profile is easily retrieved.
- For the comb laser, **M** is rectangular and inversion is not well defined. Instead, a pseudoinverse matrix is computed in the context of a least squares problem (as *m* > *n*, the system is overdetermined):

11 $$\begin{array}{c}{{\bf{M}}}^{+}={({{\bf{M}}}^{{\rm{T}}}{\bf{M}})}^{-1}{{\bf{M}}}^{{\rm{T}}}\,{\rm{a}}{\rm{n}}{\rm{d}}\,{\bf{x}}={{\bf{M}}}^{+}{\bf{b}}\end{array},$$

where **M**^T^ is the transpose of matrix **M**. However, owing to collinearity and overdetermination, ambiguity in **M** may cause large variations in **x** even with small changes in **b** (that is, the system is highly sensitive to noise). This instability is quantified using the condition number, *k*(**M**), defined as the ratio of the largest to the smallest singular values:

12 $$k({\bf{M}})=\frac{{\sigma }_{\max }}{{\sigma }_{\min }}$$

To address this, two strategies are considered:

1. Singular value decomposition. The matrix **M** is factorized as:
  13 $$\begin{array}{c}{\bf{M}}={\bf{U}}{\boldsymbol{\Sigma }}{{\bf{V}}}^{{\rm{T}}}\end{array}$$
  where **U** and **V** are orthogonal matrices of singular vectors, and **Σ** is a diagonal matrix of singular values. Singular values below a threshold are truncated to form a low-rank approximation of **M**, reducing sensitivity to noise and improving numerical stability. This results in a regularized, although approximate, solution.
2. Regularization (Tikhonov/ridge regression). A penalty term is added to control the size of **x**, preventing amplification of noise and overfitting:
  14 $$\begin{array}{c}\mathop{\min }\limits_{x}(| | {\bf{M}}{\bf{x}}-{\bf{b}}| {| }^{2}+{\lambda }^{2}| | {\bf{L}}{\bf{x}}| {| }^{2})\end{array}$$
  Here *λ* is the regularization parameter that controls the smoothness, and **L** is a matrix defining the nature of the constraint. In our case, **L** includes: **D**_1,_ the central difference matrix of the first derivative; and **D**_2_, the central difference matrix of the second derivative. We obtain
  15 $${{\bf{x}}}_{{\lambda }_{1}{\lambda }_{2}}=({{\bf{M}}}^{{\rm{T}}}{\bf{M}}+{\lambda }_{1}{{\bf{D}}}_{1}^{{\rm{T}}}{{\bf{D}}}_{1}+{\lambda }_{2}{{\bf{D}}}_{2}^{{\rm{T}}}{{\bf{D}}}_{2}{)}^{-1}{{\bf{M}}}^{{\rm{T}}}{\bf{b}}.$$
  This preserves the general shape of the original profile while suppressing high-frequency noise. Parameters *λ*_1_ and *λ*_2_ are selected via cross-validation.

#### Experiment

The QCL emission is first characterized with an FTIR set-up by sweeping the RF injection frequency. The matrix **M** is then constructed: *m* rows represent RF sweep points, *n* columns represent THz frequency peaks, and each element corresponds to the lasing intensity of a mode at a given RF frequency and THz emission.

Owing to slight shifts in central THz frequencies as the RF is swept, 8 THz bands are defined: [2.9348, 2.9417, 2.9510, 2.9609, 2.9708, 2.9780, 2.9844, 2.9937]. The THz maximum standard error of the mean is 1 GHz.

The aim of the experiment is to detect a water vapour absorption line centred at approximately 2.97 THz. The laser emission is focused through a double parabolic mirror set-up onto a pyroelectric detector. The laser is operated in continuous-wave mode with RF injection. A mechanical chopper modulates the path at 30 Hz (optimal for pyroelectric detector response). The RF is swept across 10 points (7.15–12.15 GHz), and the integrated detector intensity is averaged over 200 iterations. Measurements are performed under two conditions: (1) nitrogen-purged environment (reference: minimal water vapour absorption); and (2) ambient humidity (with water vapour).

The **b** vector (integrated intensity) is computed by normalizing the humid measurement with respect to the nitrogen baseline. An illustration of the measurement set-up and plots of the experimental results are shown in Fig. 5.

## Online content

Any methods, additional references, Nature Portfolio reporting summaries, source data, extended data, supplementary information, acknowledgements, peer review information; details of author contributions and competing interests; and statements of data and code availability are available at https://doi.org/10.1038/s41586-026-10387-w.

## Supplementary information

Supplementary Information Supplementary Discussion and Notes containing additional measurement and simulation results that support the findings of the article. It comprises sections 1–10, including Supplementary Figs. 1–15 containing details such as SWIFT spectroscopy measurements, comb properties and generalizations, experimental parameter sweeps, and simulated comb properties.

Supplementary Video 1 Simulation video at 6 GHz. Animation of the spatiotemporal evolution of the optical and microwave fields at a driving frequency of 6 GHz, displaying slow optical pulse propagation.

Supplementary Video 2 Simulation video at 7 GHz. Animation of the spatiotemporal evolution of the optical and microwave fields at a driving frequency of 7 GHz, displaying resonant pulse propagation at the normal group velocity.

Supplementary Video 3 Simulation video at 9 GHz. Animation of the spatiotemporal evolution of the optical and microwave fields at a driving frequency of 9 GHz, displaying fast optical pulse propagation.

Supplementary Video 4 Simulation video at 15 GHz. Animation of the spatiotemporal evolution of the optical and microwave fields at a driving frequency of 15 GHz, displaying fast double optical pulse propagation.

Peer Review File

## Source data

Source Data Fig. 1

Source Data Fig. 2

Source Data Fig. 5

## Figures and Tables

**Extended Data Table 1:**
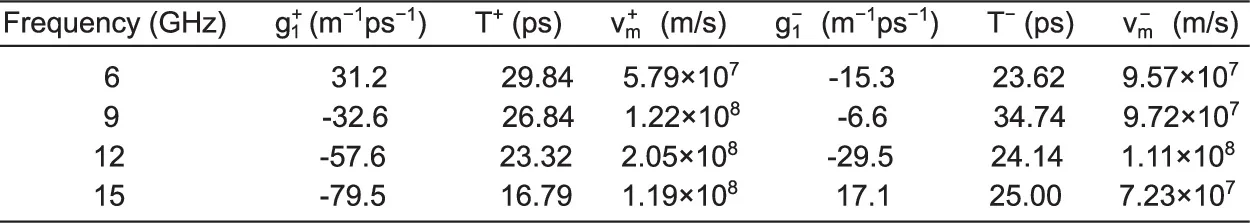
Numerical model simulation results with extracted pulse parameters

The simulations were performed in different regimes (single slow pulse, single fast pulse, double fast pulse), mostly at the same frequencies as studied in the Main Text Fig. 4. The parameters describe the gain slope ${g}_{1}^{\pm }$, the pulse duration *T*^±^, and the group velocity ${v}_{m}^{\pm }$ for the forward and backward propagation direction. Good agreement is found between the numerical and analytical model results.

**Extended Data Table 2:**
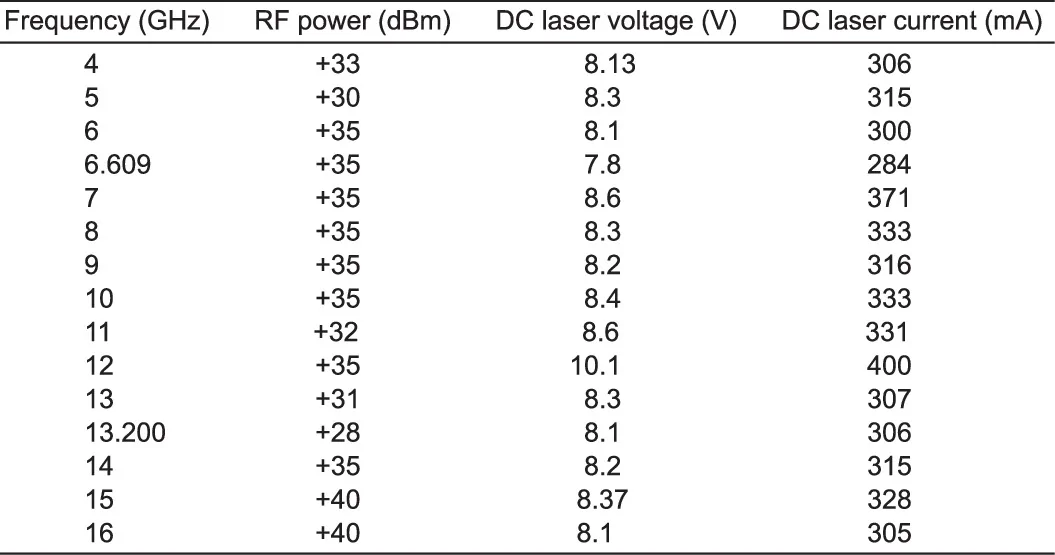
Experimental SWIFT spectroscopy measurement parameters

This table lists the experimental parameters, namely the RF frequency, RF power at the source, and DC laser bias (voltage and current) used with the planarized ridge sample studied in Main Text Figs. 3, 4, and in Extended Data Figs. 2, 3. As mentioned, the tapered waveguide sample, which was used for the absorption spectroscopy experiment in the Main Text Fig. 5, instead produces tunable comb spectra with a stable central frequency. This is achieved while keeping all the other parameters fixed, tuning only the RF modulation frequency, making the system very straightforward for use in applications. See also Extended Data Fig. 4 for measurement results on another tapered waveguide device.

## Data Availability

All the simulation and experimental data supporting this study are available from the corresponding authors upon request. The data used to generate the plots in Figs. 1–5 are published on figshare^56^. Source data are provided with this paper.

## Code availability

The code used to model the QCL active region is available in the public repository: https://github.com/cph-tum/monacoQC. Further information on the simulation code is available from the corresponding authors upon request.
