# Supplementary material for: Continuously tunable coherent pulse generation in a semiconductor laser
Source: Nature. 2026 Apr 15;652(8111):892–8. doi: 10.1038/s41586-026-10387-w (PMC13661238; doi:10.1038/s41586-026-10387-w)
Supplement: Supplementary file 1 [file 41586_2026_10387_MOESM1_ESM.pdf]

nature portfolio

https://doi.org/10.1038/s41586-026-10387-w

Supplementary information

Continuously tunable coherent pulse generation in a semiconductor laser

In the format provided by the authors and unedited

Supplementary Information for Continuously tunable coherent pulse generation in a semiconductor laser

Urban Senica[1,2*], Michael A. Schreiber[3], Marco Raffa[1], Paolo Micheletti[1], Mattias Beck[1], Christian Jirauschek[3], Jérôme Faist[1], Giacomo Scalari[1*]

[1]Institute for Quantum Electronics, ETH Zürich, 8093 Zürich, Switzerland
[2]John A. Paulson School of Engineering and Applied Sciences, Harvard University, 02138 Cambridge, USA
[3]TUM School of Computation, Information and Technology, Technical University of Munich (TUM), 85748 Garching, Germany

*To whom correspondence should be addressed;
E-mail: usenica@seas.harvard.edu, scalari@phys.ethz.ch

Contents

S1 Resonant modulation 3

S2 SWIFT spectroscopy frequency sweep 3

S3 Comb properties and generalizations 4

S4 Simulated intracavity fields 5

S5 Light-Current-Voltage Characteristics 7

S6 Modulation Frequency Limits 8

S7 Laser Bias Sweep 11

S8 Central Emission Frequency Stability and Tunability 12

S9 Output Power Dependence on Detuning 15

S10 Simulated Comb Properties 17
S10.1 Build-up Dynamics . . . 17
S10.2 Single vs Double Pulse Regime . . . 18
S10.3 Dependence on the DC Operating Point . . . 20
S10.4 Dependence on the Modulation Strength . . . 20
S10.5 Increased Microwave Attenuation . . . 20

References 23

In the following, we present additional findings along with experimental and simulation results on planarized THz QCL devices,[1] supporting the main manuscript's findings. Unless stated otherwise, these correspond to the 6 mm long, 40 μm wide homogeneous ridge sample.

S1 Resonant modulation

In Extended Data Fig. 2, we show SWIFT spectroscopy results under standard (resonant) driving conditions. Here, the microwave injection frequency is close (to within a few MHz) to the natural repetition rate, and resonant modulation sidebands are generated, which can injection-lock the longitudinal cavity modes, stabilizing the periodic laser output and broadening the emission spectrum.[2,3] In the case of fundamental and second harmonic mode-locking of the homogeneous ridge sample in Extended Data Fig. 2, the spectrum product measured directly with a slow detector (DTGS) and the SWIFT spectrum obtained via the optical beatnote using a fast detector (Schottky diode) have an excellent agreement. The latter allows us to obtain the intermodal phase differences and reconstruct the time-domain output profile, producing spectral bandwidths up to 500 GHz and short pulses down to 5.6 ps under resonant modulation conditions. The exact resonant frequency was found experimentally by biasing the laser at the threshold and performing a fine sweep of the modulation frequency within ± 10 MHz around the measured free-running repetition rate.

We should note that with active mode-locking, the obtainable pulse durations are in the several picoseconds to tens of picoseconds range, as is demonstrated in this work. This is related to inherent pulse duration limitations of this technique due to gain-induced pulse broadening, as explained, for example, in the Kuizenga-Siegman model[4] and the Haus mode-locking theory.[5] Ultrashort (femtosecond) pulses are accessible with passive mode locking, e.g., using a saturable absorber.[6] However, in contrast to our work, with passive mode locking, there is no tunable parameter that would enable a significant tunability of the repetition rate of a monolithic device (without changing the physical cavity dimensions). The pulses generated with our device are comparably short in perspective to the THz carrier frequency, i.e., a pulse encompasses roughly 17 optical cycles for resonant modulation. According to Extended Data Fig. 2, the comb spectrum then spans approximately 20% of the center frequency.

S2 SWIFT spectroscopy frequency sweep

In Extended Data Fig. 3, we present the full SWIFT spectroscopy results at each modulation frequency between 4-16 GHz, showing the spectrum product, SWIFT spectrum, and the extracted intermodal phase differences. The same spectral bandwidth window of 400 GHz is used in each panel for a better comparison. The spectrum products are color-coded depending on the specific modulation frequency, i.e., red for $f_{\mathrm{mod}} < f_{\mathrm{rep},0}$, green for $f_{\mathrm{rep},0} < f_{\mathrm{mod}} < 2f_{\mathrm{rep},0}$, and blue for $f_{\mathrm{mod}} > 2f_{\mathrm{rep},0}$. This is the same set of measurement results as presented in the 2D colormap in Fig. 3a in the main text. As can be seen, for each frequency point, the intermodal phase

differences are nearly perfectly flat across the measured spectrum, which would correspond to Fourier transform-limited mode-locked short pulses (see also the main Fig. 3 for the time-domain results). The spectra are broader for modulation frequencies closer to the fundamental or harmonic of the natural repetition rate $f_{\mathrm{rep},0}$= 6.61 GHz. We should note that the SWIFT spectra typically have a worse signal-to-noise ratio than the spectrum products, which is due to a lower sensitivity of the Schottky diode fast detector as compared to the DTGS slow detector. This does not occur due to any lack of coherence of the weaker modes, but simply because their intensities are too low to be picked up by the Schottky diode. Additionally, for high modulation frequencies above $2f_{\mathrm{rep},0}$, the measured spectral bandwidths are narrower due to a reduced power output from the RF amplifiers and increasing RF cable and connector losses. For low modulation frequencies below $f_{\mathrm{rep},0}$ (bottom row in Extended Data Fig. 3), the spectral comb lines appear to be broadened, however, this is only a numerical artifact of the Fourier transform of the finite-length interferogram. This effect is more pronounced at lower modulation frequencies, as the mode spacing is approaching the FTIR resolution limit of 2.25 GHz, and is not related to any real broadening of the comb lines. Despite approaching this measurement resolution limit, we were able to retrieve the correct mode amplitudes, frequencies, and intermodal phases, validating the coherence of the comb states and reconstructing the emission of coherent pulse trains in the time domain.

In Extended Data Table 2, we summarized all the crucial experimental parameters used for the SWIFT spectroscopy measurements, which were performed at a fixed heat sink temperature of 40 K. The reported RF power is for the set power at the RF source without accounting for cable and sample coupling losses.

S3 Comb properties and generalizations

In our experiments, the highest obtainable modulation frequency of 19 GHz was limited by the upper-frequency cut-off of the microwave generator and amplifier equipment. Numerical simulation results suggest that the modulation frequency can in principle be very high, with a fundamental upper limit imposed by the laser gain bandwidth. On the other hand, the lowest modulation frequency where coherent mode-locked pulses were still observed experimentally was at f_{mod} = 3.5 GHz, which is slightly above $f_{\mathrm{rep},0}/2$ = 3.3 GHz. Our measurements indicate that with such a slow modulation, the laser operation shifts towards a gain-switched regime,[7] producing broadband, low-coherence emission.[8] A more detailed investigation can be found in the later section S6.

Additionally, we can evaluate several of the tunable comb properties with numerical simulations. To prove that the simulated optical fields are mode-locked, we evaluate the power and phase noise quantifiers $M_{\Delta\mathrm{P}}$ and $M_{\Delta\Phi}$ from Ref.[9] This analysis shows that all injection frequencies in Fig. 3 of the main text fulfill the mode-locking conditions, i.e. $M_{\Delta\mathrm{P}} < 0.005\,P_{\mathrm{av}}$ and $M_{\Delta\Phi} < 0.02$, where P_{av} denotes the roundtrip-averaged optical power. As shown in Extended Data Fig. 7 and Fig. S11, the comb is typically stabilized within around 1 μs, amounting

to $\sim 10^4$ completed cavity roundtrip times. A necessary condition for the formation of these coherent states is a sufficient modulation amplitude across the entire laser cavity. The tunable coherent pulse train does not form with low modulation amplitudes or with large microwave propagation attenuation, where the microwaves are absorbed before reaching the front laser facet. Another requirement is that the laser is biased close to the lasing threshold, as this generates dynamic regions of both gain and loss. With an increasing DC laser bias, the laser regime first transitions to broader emission spectra, where individual sub-combs still feature the mode spacing following the microwave modulation frequency but are separated into several spectral portions with different f_{ceo} offset frequencies (see also Extended Data Fig. 6). A further increase of the laser bias results in emission spectra with the natural mode spacing imposed by the cavity boundary conditions. This occurs due to an increasing gain saturation, which decreases the effective modulation amplitude, eventually hindering the formation of tunable spectra. A more detailed study of these aspects can be found in the later sections S7 and S10.

S4 Simulated intracavity fields

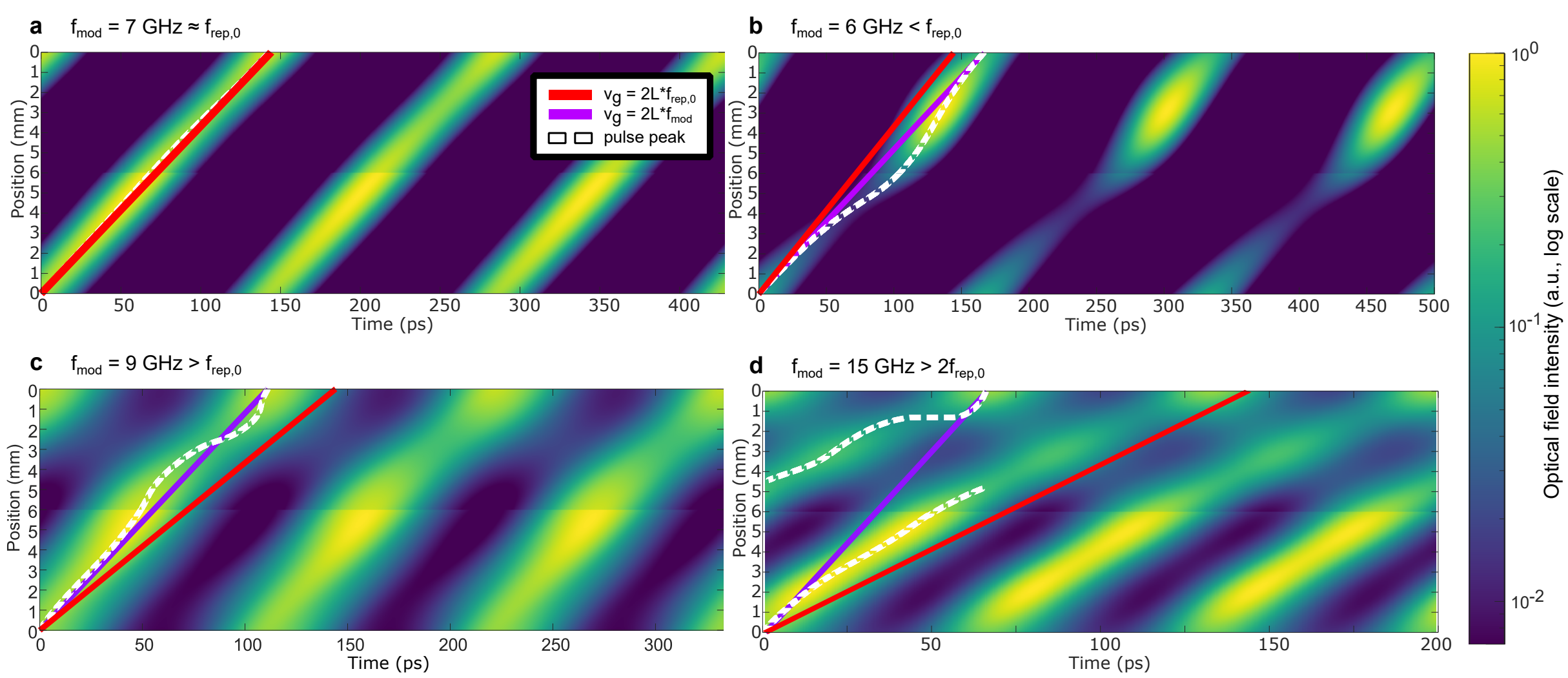

Figure S1: Comparison of different modulation frequencies of 7 GHz $\approx f_{\mathrm{rep},0}$, 6 GHz $< f_{\mathrm{rep},0}$, 9 GHz $> f_{\mathrm{rep},0}$, and 15 GHz $> 2f_{\mathrm{rep},0}$, showing three roundtrip times of each periodic waveform in the unfolded cavity representation in the laboratory reference frame.

In this section, we provide additional detailed information and visualizations of the intracavity fields presented in Fig. 4 of the main text. The diverse landscape of intracavity waveform dynamics showcased in the selected frequency examples is related to the specific spatiotemporal distribution of the modulation microwaves, which depends on the electrode/microwave waveguide geometry and its frequency-dependent properties, such as impedance, losses, reflectivity, and the resulting interference between counter-propagating microwaves, as shown already in Fig. 1c.

This also influences the resulting output pulses in terms of their duration, shape, and asymmetry, as observed in experiments and simulations.

In Fig. S1, we plot the intracavity optical field intensities in real space-time coordinates, i.e., in the laboratory reference frame. In this view, the group velocity is proportional to the slope of the propagating optical pulse. The red lines mark the reference group velocity in the resonant modulation case, while the violet lines show the roundtrip-averaged group velocity for each modulation frequency, defined as $v_\mathrm{g} = 2Lf_\mathrm{mod}$. The white dashed lines track the peak of the optical pulse as it propagates within the cavity, completing a single roundtrip by traversing the diagonal direction (violet line). Any deviation of the white dashed line is proportional to a variation in the local group velocity. In this laboratory reference frame, it is more straightforward to study the exact pulse shape, amplitude, and variation of group velocity in real space-time coordinates. This is especially useful for studying the case of $f_\mathrm{mod} = 15$ GHz $> 2f_\mathrm{rep,0}$, where it is clearly visible that at any given time t, there are two counterpropagating pulses present in the cavity, and the group velocity of each single pulse is not much greater than the natural one (white dashed vs. solid red line).

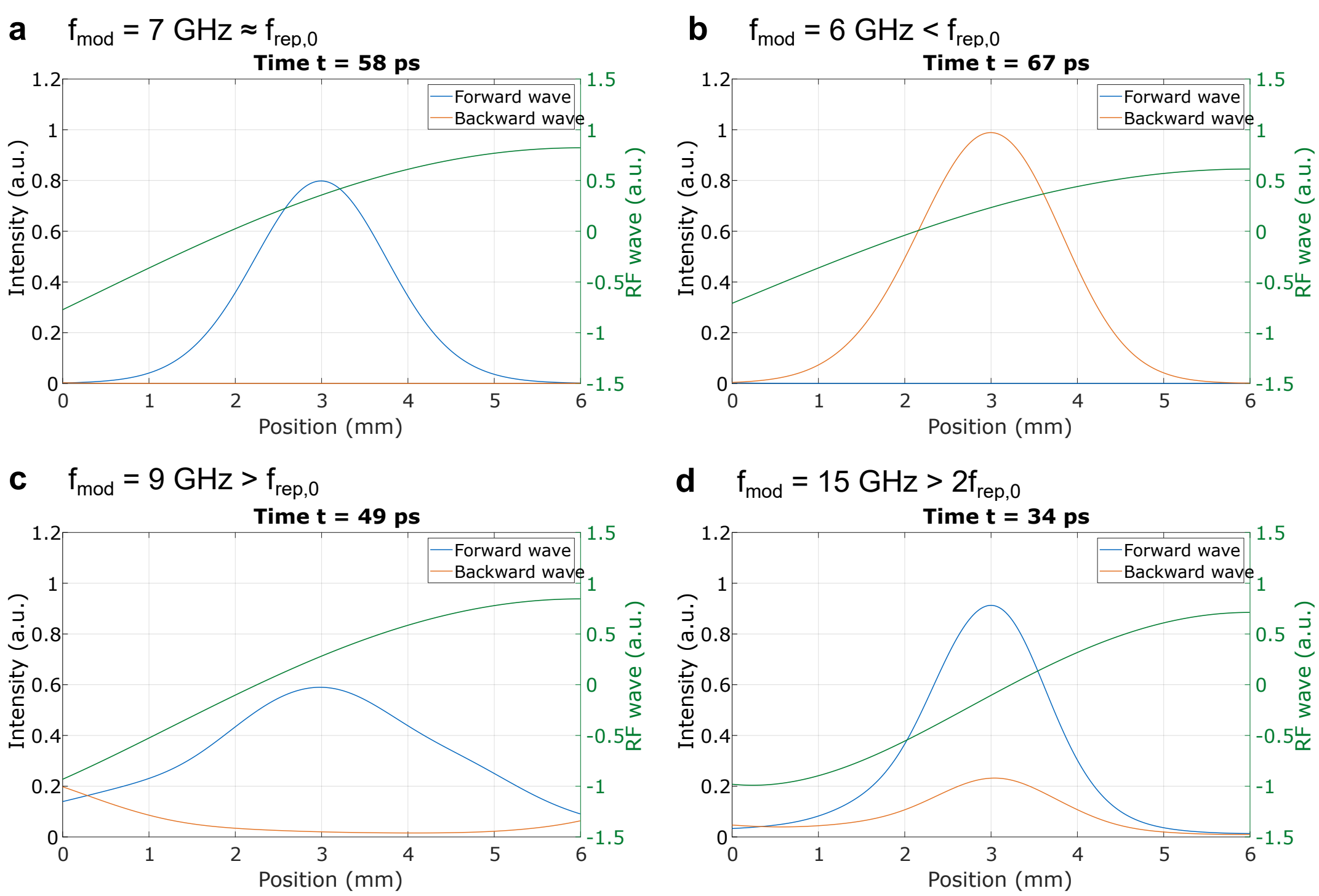

Figure S2: Snapshots of the animated time evolution of the microwave and optical fields (movies are available in the online publication).

For all of the presented modulation frequency examples, we also generated movies of the intracavity microwave and optical fields, which can be studied dynamically by playing the videos or frame-by-frame. In Fig. S2, we show a single frame for each case, while full movies are

available in the online publication.

S5 Light-Current-Voltage Characteristics

In Fig. S3, we show the Light-Current-Voltage (LIV) characteristics measured in pulsed (10% duty cycle, 500 ns pulses) and in free-running continuous-wave (CW) operation at a heat sink temperature of 40 K. The laser sample displays a relatively low threshold current of 305 mA (corresponding to a threshold current density of 127 mA/cm^2) and a peak output power of 2.5 mW.

Additionally, we performed a study of the laser IV characteristic curves of the planarized ridge sample in CW with an additional microwave modulation, as a function of the modulation frequency. These were performed with a power of +32 dBm at the RF source and a heat sink temperature of 40 K, where the DC laser bias source was operated in voltage-driven mode with a limit of 10.2 V to avoid any potential damage to the sample. As shown in Fig. S4, both the threshold current as well as the IV curve slope (proportional to the differential resistivity) depend on the modulation frequency. While the lowest threshold is observed for a resonant modulation at the natural repetition frequency, the highest is obtained for a modulation frequency of 12 GHz. These modulation frequency-dependent electrical properties affect both the varying DC laser bias and the related central emission frequency shift.

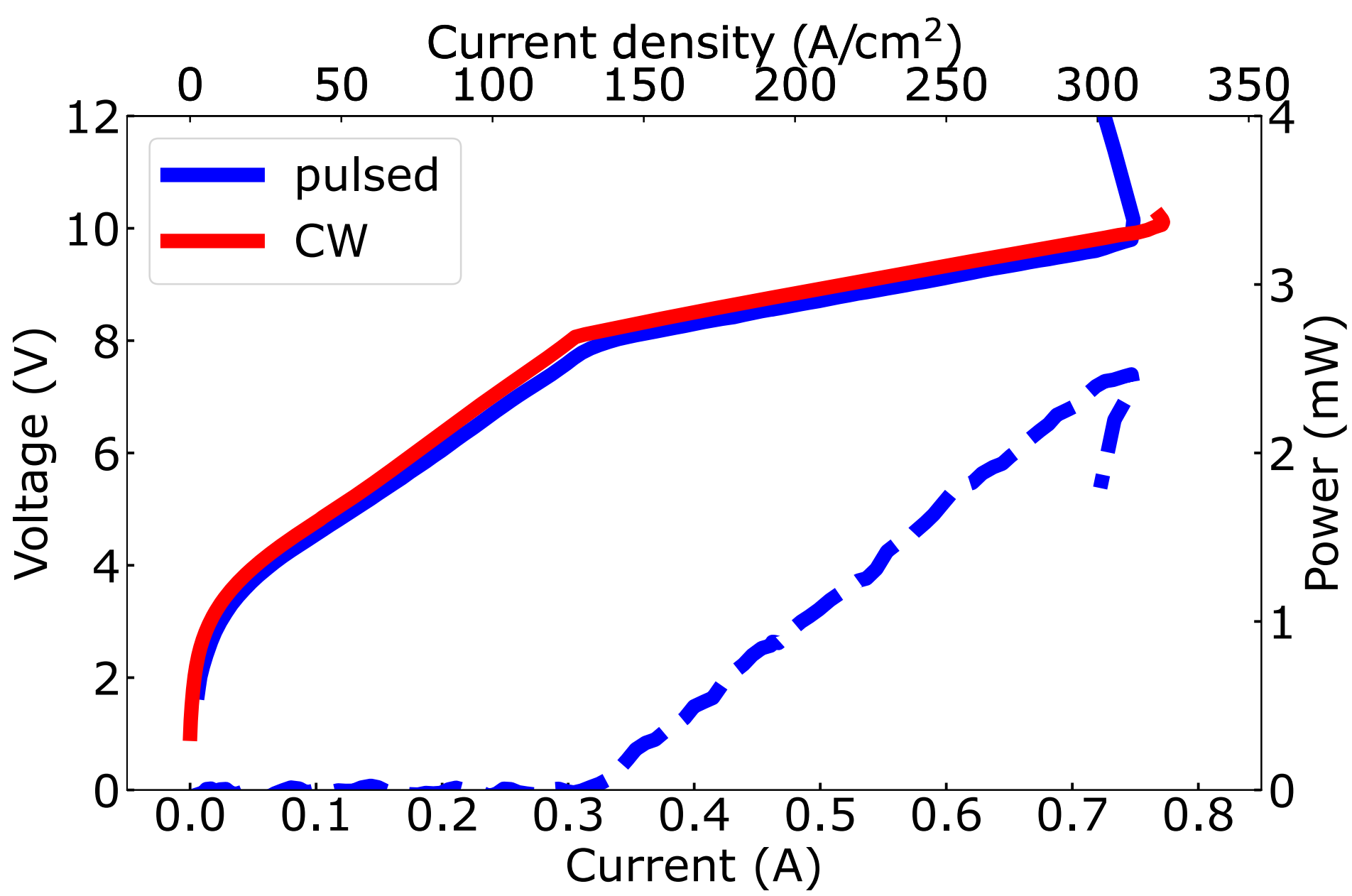

Figure S3: LIV curves measured in pulsed mode (10% DuC, 500 ns pulses) and in CW at a heat sink temperature of 40 K.

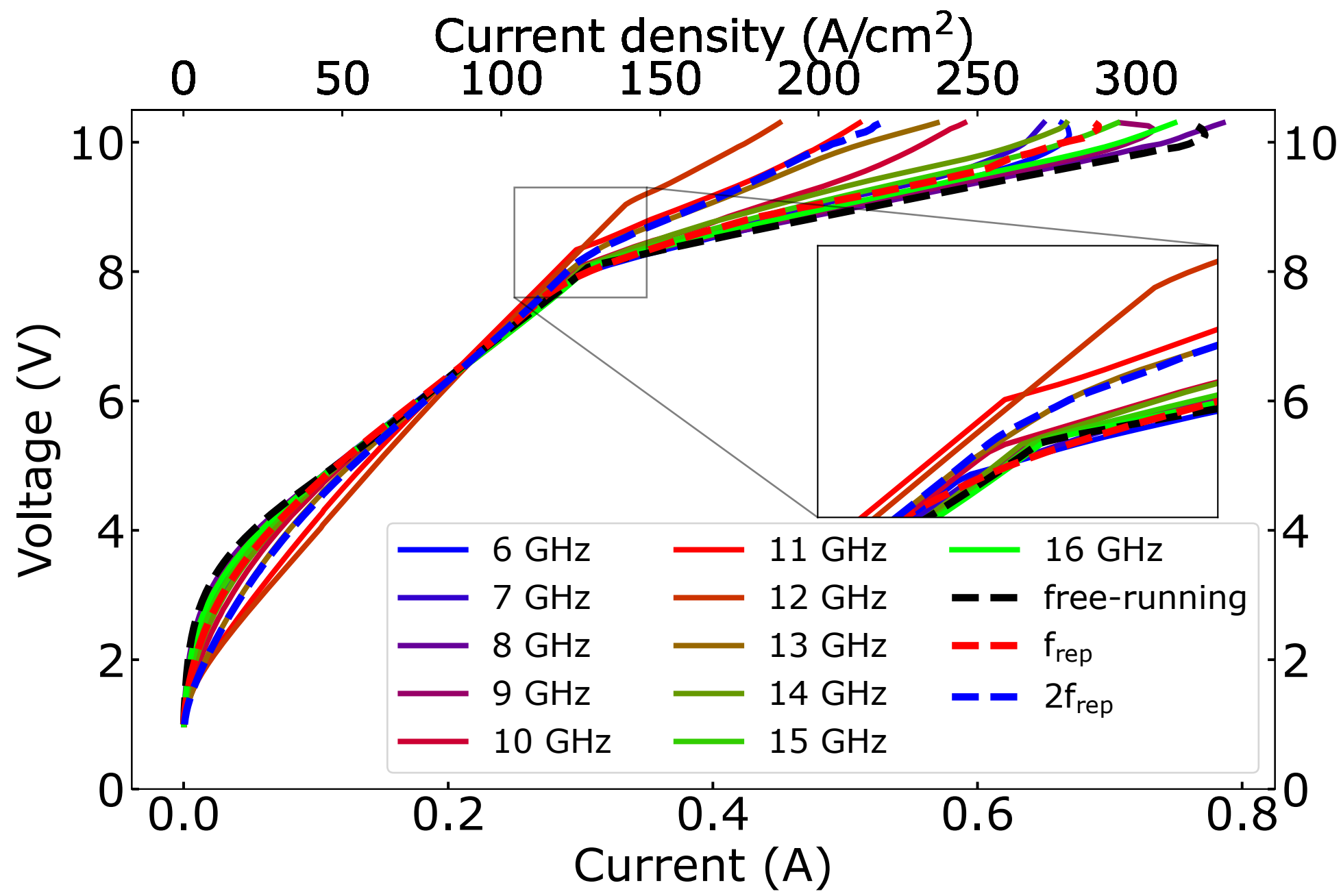

Figure S4: IV curves measured with different microwave modulation frequencies with a power of +32 dBm at the source and a heat sink temperature of 40 K.

S6 Modulation Frequency Limits

We also investigated the modulation frequency limits for obtaining the tunable coherent pulse states.

In principle, very high modulation frequencies should be possible, provided that the modulation amplitudes are large, the microwave losses are low, and the modulation frequency is lower than the laser gain bandwidth. Under these conditions, a sufficient spatiotemporal gain modulation along the entire laser cavity would support the formation of tunable mode-locked pulses. With increasing frequency, the microwave losses of the cables, wire bonds, as well as the propagation losses within the planarized waveguide sample itself, can increase significantly. These can occur due to conductor losses (skin effect), substrate losses (loss tangent), and radiative losses, which all increase with frequency. For sufficiently large losses, the microwaves injected at the back facet would be strongly attenuated before reaching the front facet, hindering the formation of a standing RF wave and the traveling gain window.

To study the microwave losses of our system, we performed frequency-dependent eigenmode numerical simulations of the planarized waveguide 2D cross-section. As shown in Fig. S5, at a frequency of 10 GHz, the microwave effective index is around 2, while the propagation losses are around 1.2 dB/mm, which increase exponentially with frequency (dB/mm units). These properties are also included in our extensive numerical simulations of the modulated laser cavity

in the form of a 1D microwave transmission line model.

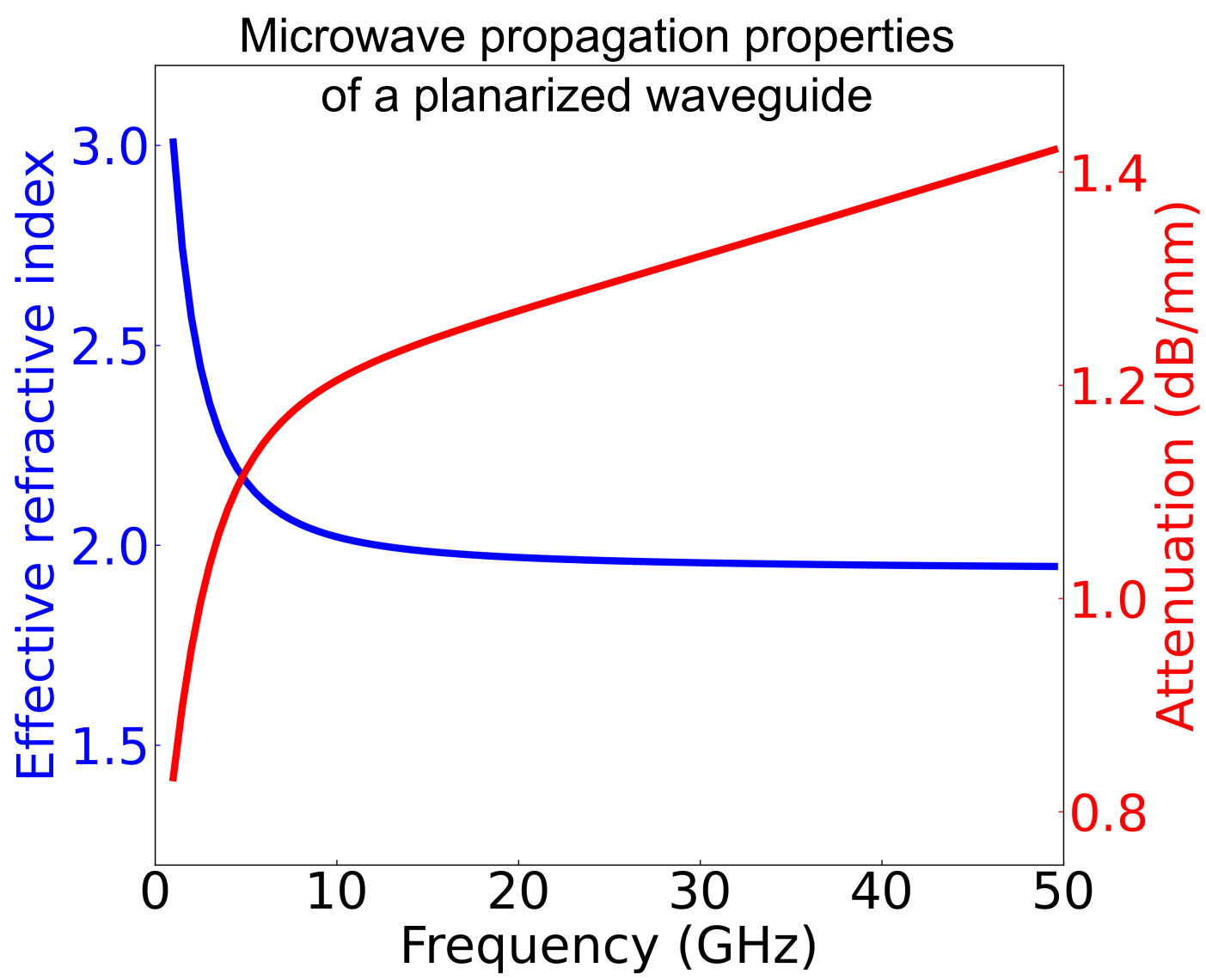

Figure S5: Simulated microwave propagation properties of a planarized waveguide with an active waveguide width of 40 μm, height of 10 μm, and top metal width of 300 μm and thickness of 500 nm.

Experimentally, in our specific system and sample, the required RF modulation powers (applied at the source) are generally at +25 dBm or above. In terms of studying the effects of microwave loss, we did not perform an experimental study as these are fixed by the sample geometry, and the highest modulation frequency of around 19 GHz was limited by our RF source and amplifier equipment. Therefore, we could not identify the highest modulation frequency experimentally. However, we explored the limitations with numerical simulations. As shown in Fig. S6, very high modulation frequencies are in principle possible (here shown for f_{mod} = 35 GHz $> 5 f_{\mathrm{rep},0}$). Such high modulation frequencies have already been demonstrated for resonantly modulated THz QCLs (see Refs.[10,11]). Additionally, we performed a numerical simulation study, where we show that in the case of significantly increased microwave propagation loss (4.3 dB/mm), the output pulse stream experiences intensity fluctuations and does not support a coherent mode-locked state, resulting instead in a noisy emission spectrum (more details are in the Section S10.5 and Fig. S15).

In terms of the theoretical high-frequency modulation limit, neglecting the limitations of RF equipment and microwave loss that we discussed in the previous paragraphs, there are two additional limiting factors: the gain bandwidth and the gain recovery time. High-frequency modulation is also closely related to harmonic mode locking, since several pulses will be propagating within the cavity simultaneously. With increased repetition rates, the pulses are shorter and closely spaced in time. In this context, it was found that short gain recovery times support the propagation of harmonic pulses.[12] Additionally, even with extremely fast gain

dynamics, the final limitation is the gain bandwidth:[12] shorter pulses feature larger mode spacings and broader optical bandwidths; however, only emission frequencies that lie within the laser gain bandwidth can be generated, since the active material needs to provide sufficient gain to overcome the roundtrip losses to sustain lasing action. Therefore, the gain bandwidth needs to be sufficiently wide, such that several modes can fit within this bandwidth for the given modulation frequency. In our case, the active region we used has a relatively broad gain bandwidth of around 1 THz, and a relatively short gain recovery time of around 10 ps, so theoretically, modulation frequencies in the order of around 100 GHz could be possible.

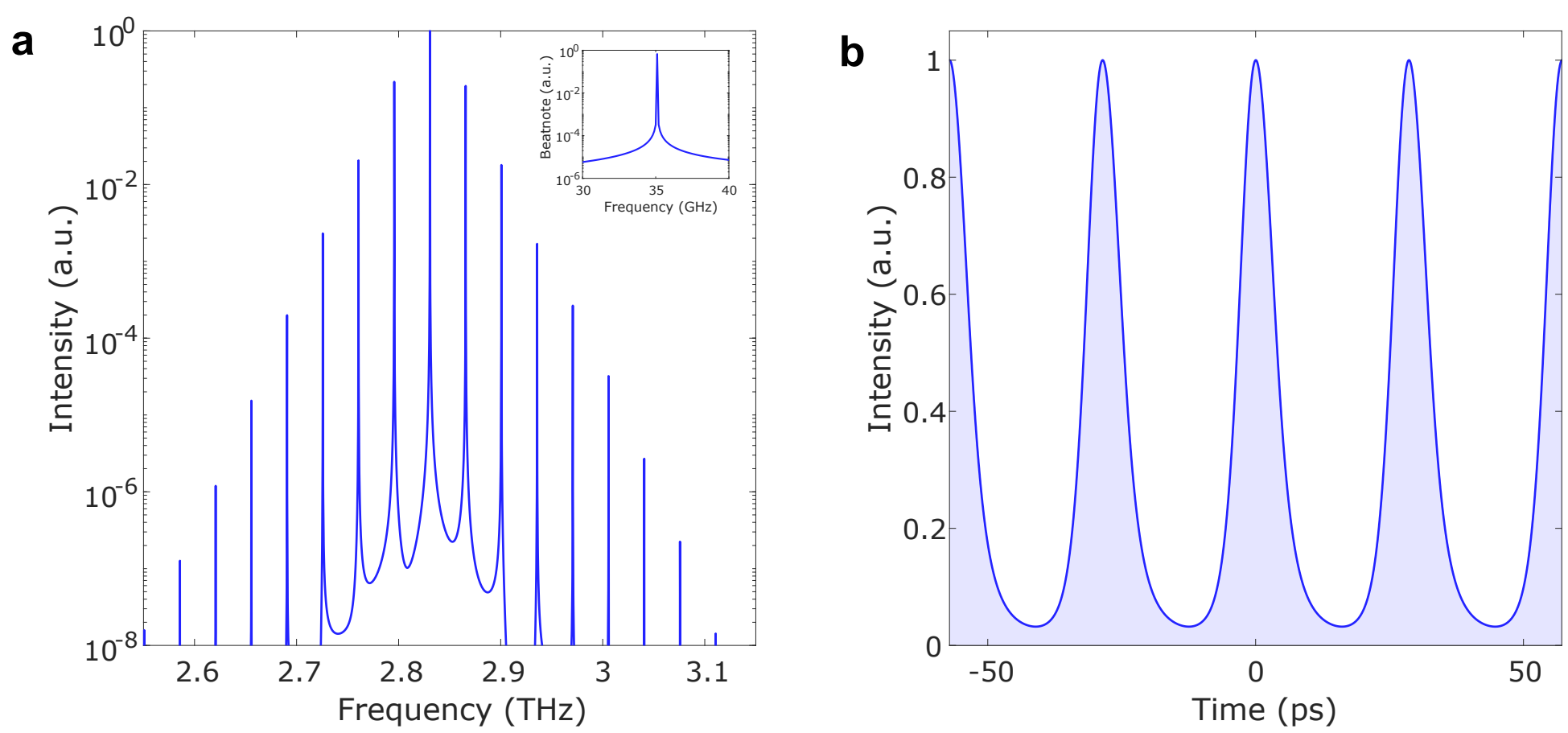

Figure S6: Simulation result with a modulation frequency of f_{mod} = 35 GHz $> 5f_{\text{rep},0}$. This simulation shows that the tunability is, in principle, possible for much higher modulation frequencies than the current experimental limit. **a** displays the intensity spectrum and **b** the corresponding time domain data.

On the low-frequency end, the lowest experimentally obtainable modulation frequency was 3.5 GHz, which is close to $f_{\text{rep},0}/2 = 3.3$ GHz. There are two competing operation regimes, as shown in Extended Data Fig. 5. The modulation amplitude needs to be large enough for the spatiotemporal gain modulation to generate the tunable coherent pulse, as shown in panel a for an RF injection power of +30 dBm. However, if the modulation power is further increased to +32 dBm, the laser enters a gain-switched regime,[7] producing broadband, low-coherence emission with a mode spacing of $f_{\text{rep},0}/2$. In this case, the amplitude and duration of the negative RF half-cycle are so significant that the pulse gets fully attenuated (absorbed). In other words, it is energetically more favorable for the laser to enter a gain switching regime, where pulses are propagating with the normal group velocity, but are fully absorbed between each modulation period, with a new pulse generated each time, i.e., an incoherent pulse stream. Additionally, for very low modulation frequencies, we are approaching the quasi-static regime for the RF field (subwavelength dimensions with reduced RF field modulation along the cavity), another factor contributing to a smaller gain slope (see also the simulated RF field distributions in Fig. 2c of

the main text).

Considering the combination of all these effects, this means that below the low-frequency limit, there is no suitable amplitude of the RF modulation field that would be simultaneously strong enough to generate tunable pulses and weak enough not to fully attenuate them within the slow modulation roundtrip time, hindering the formation of a coherent mode-locked state. We should note that this only happens at the low-frequency cut-off point, while for high modulation frequencies, the energy breathing effect is much smaller, since pulses are more cleanly co-propagating with the traveling gain window throughout the entire roundtrip time, without significant attenuation effects.

S7 Laser Bias Sweep

We also performed an experimental laser bias sweep. In Extended Data Fig. 6, we highlight three different operating regimes that were typically observed, in this case at a heat sink temperature of 80 K for a fixed modulation frequency of 8 GHz and RF power of +35 dBm at the microwave source. The top half of each panel shows the intensity interferogram, while the bottom half shows the emission spectrum.

In panel a, the tunable coherent pulse state is displayed, which is obtained for a relatively low laser bias close to the lasing threshold. In panel b, we show that when the bias is further increased, the emission spectrum broadens and breaks up into several spectral lobes. Each lobe still features a mode spacing that matches the modulation frequency, however, their spectral separation differs from the modulation frequency, also visible as stitching points (highlighted by the black arrows). In other words, a series of sub-combs with different offset frequencies f_{ceo} is formed. Several factors influence the formation of such a state. By breaking up into several sub-combs, i.e., into several time-delayed pulses, the gain saturation is reduced compared to a single broadband and short pulse. On the other hand, the frequency shift between individual lobes also means the average frequency offset between the lasing and cold cavity modes can be reduced, e.g., by centering each lobe on a cavity mode. This effectively reduces the modulation detuning compared to a single broadband comb spectrum.

When the laser bias is relatively high, i.e., close to the NDR, the emission spectrum is even broader, but now features the natural mode spacing imposed by the cavity boundary conditions (length). On the one hand, with increasing laser bias, the effective microwave modulation amplitude is reduced due to increased gain saturation. On the other hand, being so close to the NDR, it is also possible that the laser is switching off during the positive RF wave half cycle, producing a broadband, low-coherence state akin to gain switching[7] (see also Extended Data Fig. 5b). Since such spectra are incoherent, as is evident from the nonsymmetric and decaying interferogram in the top half of the panel, SWIFT spectroscopy cannot be used to investigate this regime. Instead, an incoherent time-resolved method would be required.

S8 Central Emission Frequency Stability and Tunability

In Fig. S7, we show measurement results on the planarized ridge sample for a modulation frequency sweep between 4 and 16 GHz with a finer step of 0.1 GHz. The emission spectra in panel a show that the central frequency shifts with the modulation frequency, especially in the range between 11-13 GHz. This is attributed to the higher DC laser bias (see also Fig. S4), variable RF modulation powers, and an additional radiative transition specific to this active region, both resulting in part of the emission spectrum being centered around 3.9 THz. Nevertheless, the mode spacing still matches exactly the modulation frequency, as confirmed in panel b.

One obvious application of the tunable combs presented in this work could be spectral measurements, for example, to obtain high-resolution absorption features of an investigated chemical compound or gas species. We demonstrated this by developing an absorption spectroscopy experiment, using a tapered planarized sample with a relatively constant central emission frequency, requiring only a simple THz intensity detector. More details can be found in the main text, Fig. 5. We also investigated a third laser sample, namely a 4.2 mm long tapered planarized waveguide,[13] where the natural repetition rate is $f_{\mathrm{rep},0} = 9.4$ GHz, measured at a heatsink temperature of 80 K, compatible with liquid nitrogen cooling (instead of liquid helium).

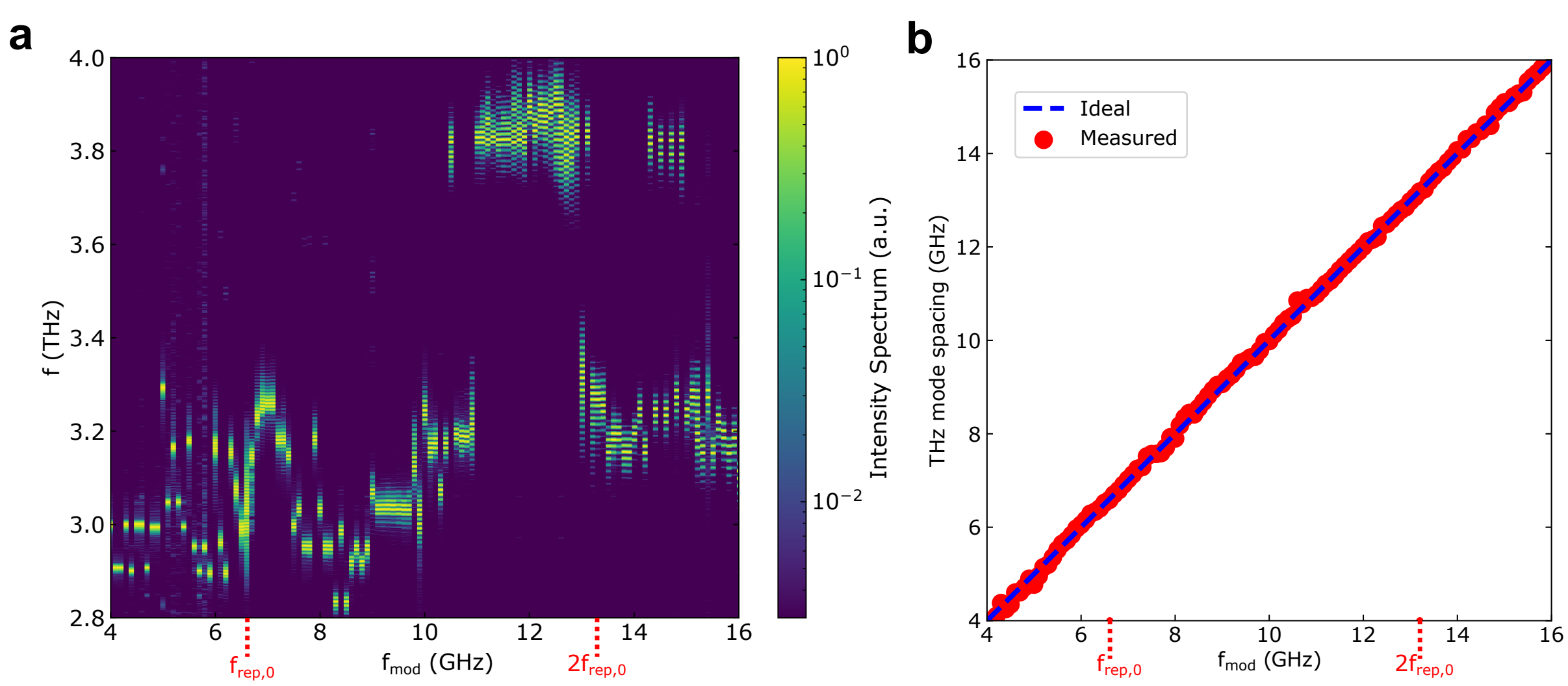

Figure S7: Experimental modulation frequency sweep between 4 and 16 GHz with a step size of 0.1 GHz for the planarized ridge sample. Both the emission spectra in **a** and the extracted mode spacing in **b** highlight the continuous repetition rate tunability over extreme values of detuning.

In Extended Data Fig. 4a, we plot the measured emission spectra for a modulation frequency sweep range between 8.5 and 18 GHz in steps of 0.1 GHz. These broadband spectra feature equidistant mode spacings that always match the microwave modulation frequency, which is visible directly from the emission spectrum map (opening diagonal lines) and also in Extended Data Fig. 4b, where we plot the extracted mode spacing as a function of the modulation frequency.

Considering the results on these three different devices, we can think about both the central frequency stability as well as its tunability, which would be useful in targeting different wavelength regions using the same device, despite the limited bandwidths of emission spectra in the tunable mode-locking regime. In terms of tuning the central emission frequency, there are several possible tuning knobs: the operating temperature, the DC laser bias, and the RF power can all contribute to shifting the emission wavelength. This is already visible in Fig. S7 of the planarized ridge sample, where different operating conditions, namely DC bias and RF power, result in a change of the central emission frequency.

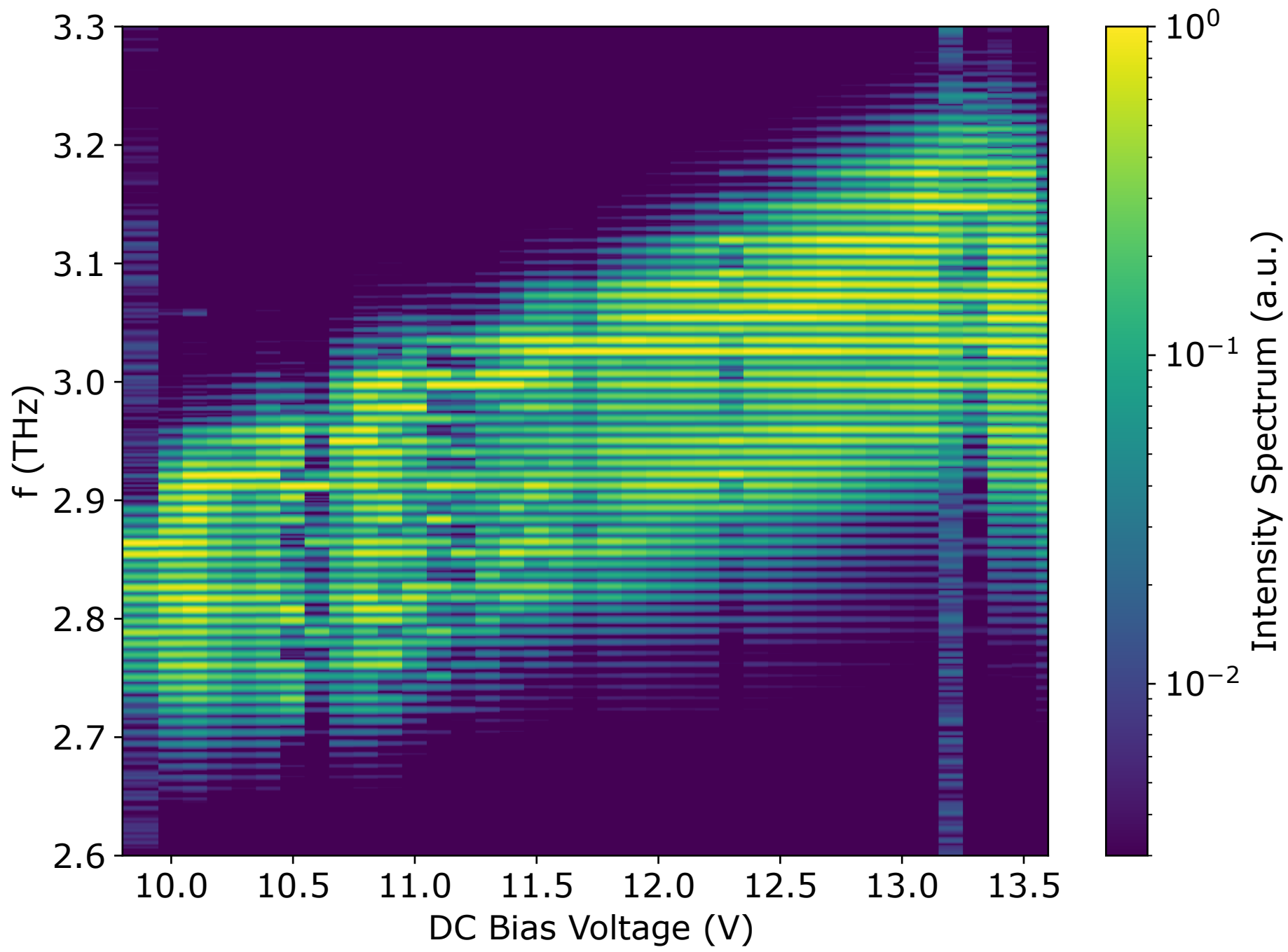

Figure S8: Experimental DC bias voltage sweep of a tapered device at resonant modulation conditions ($f_{\mathrm{mod}} \approx f_{\mathrm{rep},0}$), highlighting the blue-shifted central emission frequency with increasing bias.

In contrast, the central frequency is very stable in tapered devices, where only a sweep of the RF frequency is required, while the temperature, DC laser bias, and RF power are stabilized at constant values. By tuning one of these additional parameters, we can also tune the central emission frequency in the tapered devices. In Fig. S8, we show the emission spectra of a resonantly modulated tapered device from the previous paragraph ($f_{\mathrm{mod}} \approx f_{\mathrm{rep},0}$), where the central frequency is blue-shifted during an increasing DC laser bias sweep. We should note that in this example, we are sweeping across the entire DC bias voltage range of a resonantly modulated device, so the emission bandwidths and central frequency shifts are relatively large, but this effect can also be employed for detuned modulations within smaller ranges of DC bias voltages

(closer to the lasing threshold), where the tunable mode-locking is supported. In principle, one could also think of seeding the central emission frequency with another single-mode laser that is coupled into the tunable comb laser cavity. Additionally, if aiming for a more widely separated frequency range, THz QCLs allow for a very flexible engineering of the emission frequency by modifying the active region design, i.e., by changing the unit cell layer thicknesses of the GaAs/AlGaAs superlattice, such that specific frequency regions can be targeted.

Another related aspect is the stability and tuning of the carrier offset frequency (CEO), f_{ceo}. We should first emphasize that electrically pumped semiconductor lasers, including QCLs, are generally not CEO-stabilized, as most CEO detection techniques require an octave-spanning coherent comb spectrum for f-2f self-referencing. However, once the repetition rate is stabilized (e.g., via active mode-locking), the f_{ceo} can be tuned by an actuator, which in the case of QCLs can be achieved via the injection current (DC laser bias) or the operating temperature, which both modify the refractive index of the active material. The f_{ceo} stability is also directly related to the coherence and linewidth of the laser, and as already discussed, the tunable comb states are fully coherent, with simulations suggesting that there is no significant effect on CEO stability with modulation frequency detuning beyond the longer cavity build-up/stabilization times (see also Fig. S11).

Moreover, there is another way of directly visualizing the tunable comb data from the spectral measurements, namely plotting the interferogram traces obtained with the FTIR spectrometer, which combines the signal with its time-delayed version in a trace equivalent to an autocorrelation signal. The intensity interferograms are displayed in a 2D colormap in Fig. S9 for both the homogeneous ridge and tapered laser sample, where the horizontal axis is the variable modulation frequency and the vertical axis is the optical path delay. Within each intensity interferogram, i.e., within each vertical colored line slice in the plot, periodic intensity bursts occur at fixed positions: $N \times c/f_{\text{rep}}$, where N denotes the Nth burst, c is the speed of light in vacuum, and f_{rep} is the repetition rate that equals the spectral mode spacing. The necessary conditions for tunable, fully coherent comb states are that each interferogram is symmetric and non-decaying in amplitude (in the vertical direction) and that the measured bursts occur at the expected optical path delays. These positions were determined by using f_{mod} in the above formula and added to the figure as dashed red lines. As highlighted in Fig. S9, all of these conditions are fulfilled for every measurement across the entire modulation frequency range. This alternative way of plotting the spectral measurement data provides a straightforward visualization and verification of the tunable repetition rate across the entire modulation frequency range, regardless of any shifts in the central emission frequency or the detailed shape of the emission spectrum. We should note that in panel b, some interferograms (e.g., at 14 GHz) were not recorded for the entire available range of the FTIR spectrometer due to a malfunctioning delay stage. Nevertheless, all of the recorded bursts confirm a perfect matching to the driving modulation frequency.

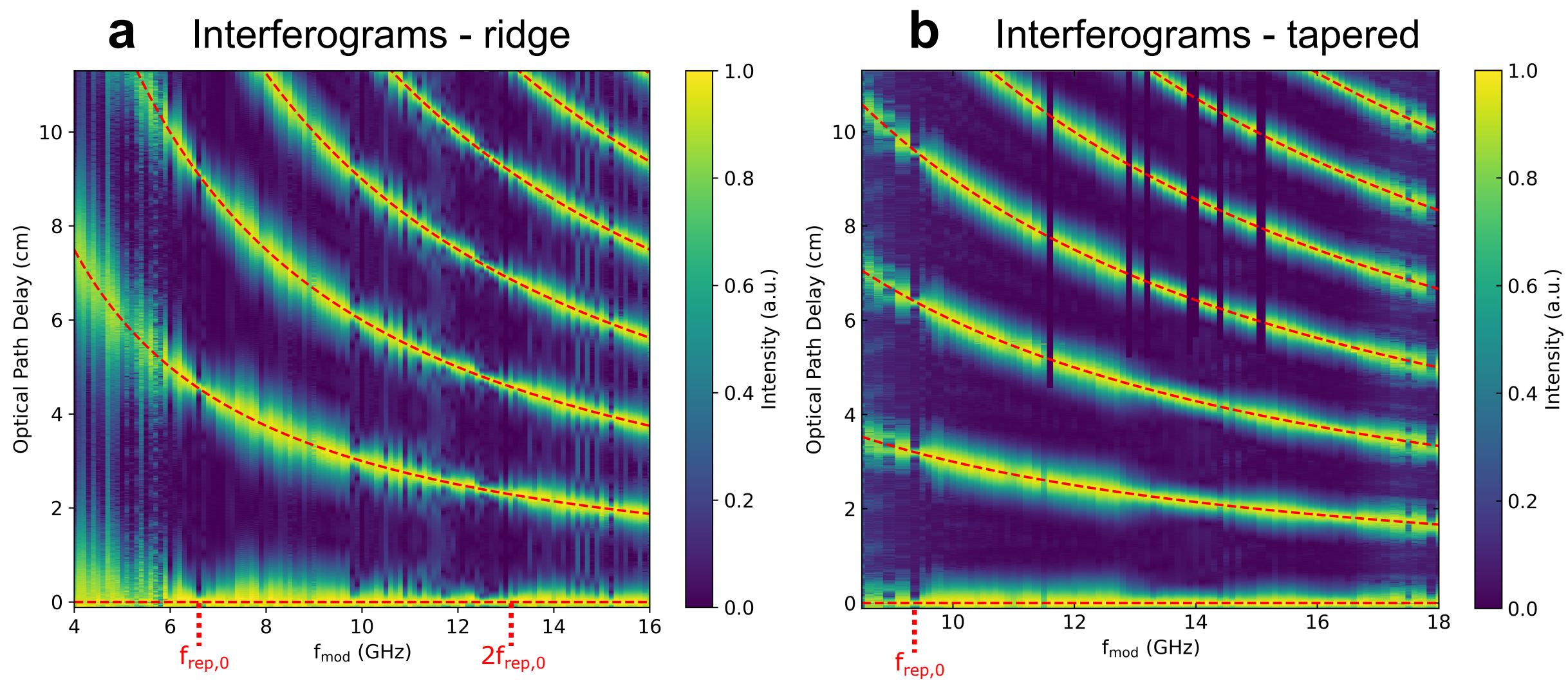

Figure S9: A 2D colourmap representation of the normalized intensity interferograms as a function of the modulation frequency for **a** the homogeneous ridge and **b** the tapered waveguide sample. The intensity bursts of the symmetric and nondecaying interferograms (along the vertical direction) match the analytically calculated positions (red dashed lines), confirming the generation of coherent tunable states in a straightforward visualization across the entire measurement range.

S9 Output Power Dependence on Detuning

In this section, we investigate the wall plug efficiency and output power dependence on the modulation frequency detuning.

Due to relatively high threshold current densities (100 - 1000 A/cm^2) and relatively high operating voltages (typically above 8 V) required to align the cascade structures, THz QCLs inherently have a significant electrical power consumption (see also Ref.[14]). In combination with limited thermal management compared to buried and/or epitaxial-side-down mounted semiconductor lasers, this results in modest wall plug efficiencies, i.e., a conversion between the electrical input power to optical output power of, at most, only a few percent ($\lesssim 2\%$).

In the case of our homogeneous ridge sample presented in the main text, at the maximum DC bias point, we are applying a voltage of 10 V and a current of 0.7 A, i.e., an electrical consumption of 7 W. The measured output in pulsed mode is around 2.5 mW. Doubling this value to include the back facet output coupling and dividing by the electrical power consumption, we get an external wall plug efficiency of around 0.1%. Here, we should emphasize that the mirror losses $a_{\mathrm{mirr}} = -\frac{1}{2L}\ln(R_1 R_2)$ are relatively low due to highly reflective cleaved facets ($R_1 = R_2 = 70\%$) and the relatively long waveguide length ($L = 6$ mm). Mirror losses are directly related to output coupling and slope efficiency via the expression $\frac{\mathrm{d}P}{\mathrm{d}I} = \frac{N_{\mathrm{p}} h\nu}{e} \frac{a_{\mathrm{mirr}}}{a_{\mathrm{tot}}} \frac{\tau_{\mathrm{eff}}}{\tau_{\mathrm{eff}}+\tau_2}$, where a_{mirr} and a_{tot} are the mirror and total loss, τ_{eff} and τ_2 are the carrier lifetimes, N_{p} is

the number of periods, ν is the laser frequency, h is Planck's constant and e is the elementary charge. For THz QCLs, the very low emission frequencies ν of around 3 THz also contribute to a relatively low slope efficiency. However, this can be improved significantly with cavity engineering, e.g., using low-reflectivity end facets and surface-emitting antennas (see also Ref.[15] , where we demonstrated a factor of 7 improvement in slope efficiency and output power). Moreover, the active material we use is optimized for broadband operation, not for high-power and high wall plug efficiency (even when optimized for those figures of merit, THz QCLs only reach around 2% wall plug efficiency, see also Ref.[16]).

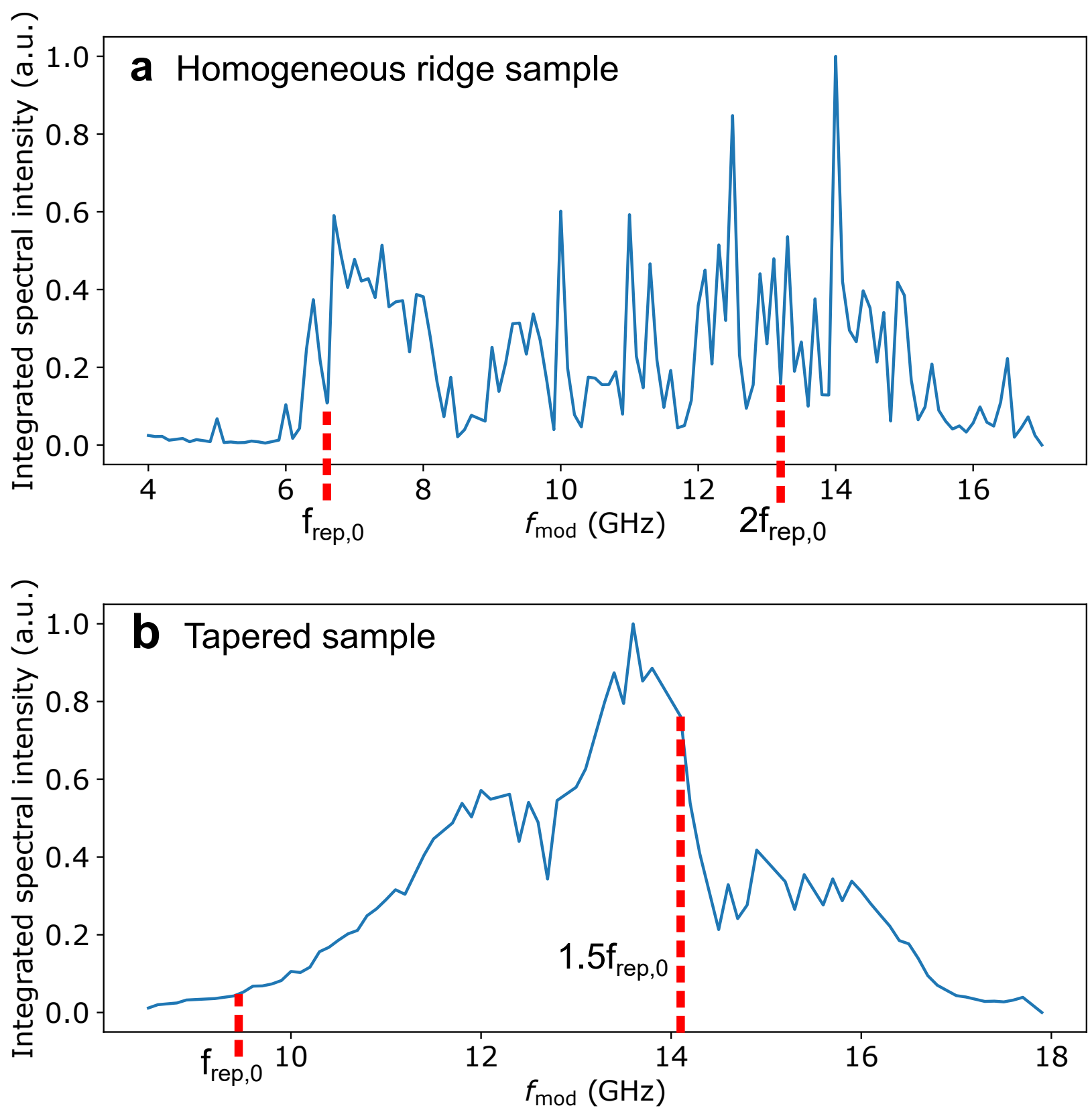

Figure S10: Integrated spectral intensity of an experimental sweep of the modulation frequency of **a** the homogeneous ridge sample and **b** the tapered sample. The latter shows a clear trend of increasing the output power between the fundamental and second harmonic cavity resonant frequency.

Regarding the dependence on detuning, intuitively, we expect the efficiency to be largest at resonant modulation ($f_{\mathrm{mod}} = N \times f_{\mathrm{rep},0}$), when the optical pulse is co-propagating with the traveling net gain window without any phase delay. In order to gain an experimental insight, we plot the integrated spectral intensity as a function of frequency detuning, normalized to the largest value over the entire sweep. In the case of the homogeneous ridge sample in Fig. S10a,

the intensity is oscillating without a clear trend, which is due to simultaneously changing the DC laser bias and RF power along with the RF modulation frequency. However, plotting the dependence for the tapered waveguide sample in Fig. S10b, where only the RF modulation frequency is changing with all the other parameters kept constant, there is in fact a clear trend. The integrated spectral intensity (proportional to the output power and wall plug efficiency) is actually increasing with the modulation frequency detuning, reaching a maximum between the resonant harmonics. The explanation is as follows: when the modulation frequency detuning is increased, the phase delay between the traveling gain window and the optical pulse increases. Additionally, the optical pulses have longer durations, i.e., they are more stretched out throughout the cavity and have lower peak intensities. In our THz QCL device with relatively short gain recovery times, these conditions result in a reduced gain saturation, effectively increasing the gain per roundtrip and generating higher output powers. In the resonant modulation case, the emission spectra are the broadest and the optical pulses are the shortest, however, the shorter optical pulses with higher peak intensities also induce a larger gain saturation. Therefore, the larger the modulation frequency detuning away from the resonant harmonics, the greater the output powers (and wall plug efficiencies), resulting in a maximum value in the proximity of $f_{\mathrm{mod}} = 1.5 \times f_{\mathrm{rep},0}$.

S10 Simulated Comb Properties

S10.1 Build-up Dynamics

The performed dynamical simulations allow us to directly track the temporal build-up of the tunable frequency comb. In Extended Data Fig. 7a, we show the simulated time trace of the optical intensity, out-coupled from the front facet of the laser resonator. In this simulation, a modulation frequency of $f_{\mathrm{mod}} = 9$ GHz $> f_{\mathrm{rep},0}$ was applied. After initial fluctuation of the intensity, the system converges to a locked state with constant pulse maxima. Extended Data Fig. 7b and 7c depict two spectra recorded at $t = 200$ ns and $t = 1180$ ns, respectively. The corresponding time intervals used for the Fourier transformations are highlighted in the time trace shown in Extended Data Fig 7a, using color-coded segments. While the first spectrum still exhibits side modes, the second spectrum shows a clean frequency comb with a mode spacing following the modulation frequency. Accordingly, a narrow RF beatnote at the modulation frequency and its higher harmonics is obtained for the steady-state case, as can be seen in the inset of Extended Data Fig. 7c.

We also performed a simulation study of the dependence of the build-up time for different modulation frequencies f_{mod}. The results of these simulations are displayed in Fig. S11. For resonant modulation (see Fig. S11 a), the system quickly stabilizes to a constant mode-locked pulse train. In contrast, the build-up time of the pulse train increases for a larger detuning of the modulation frequency from the natural repetition frequency $f_{\mathrm{rep},0}$ of the laser cavity. We should note that the build-up time generally depends on several parameters, such as the DC laser bias.

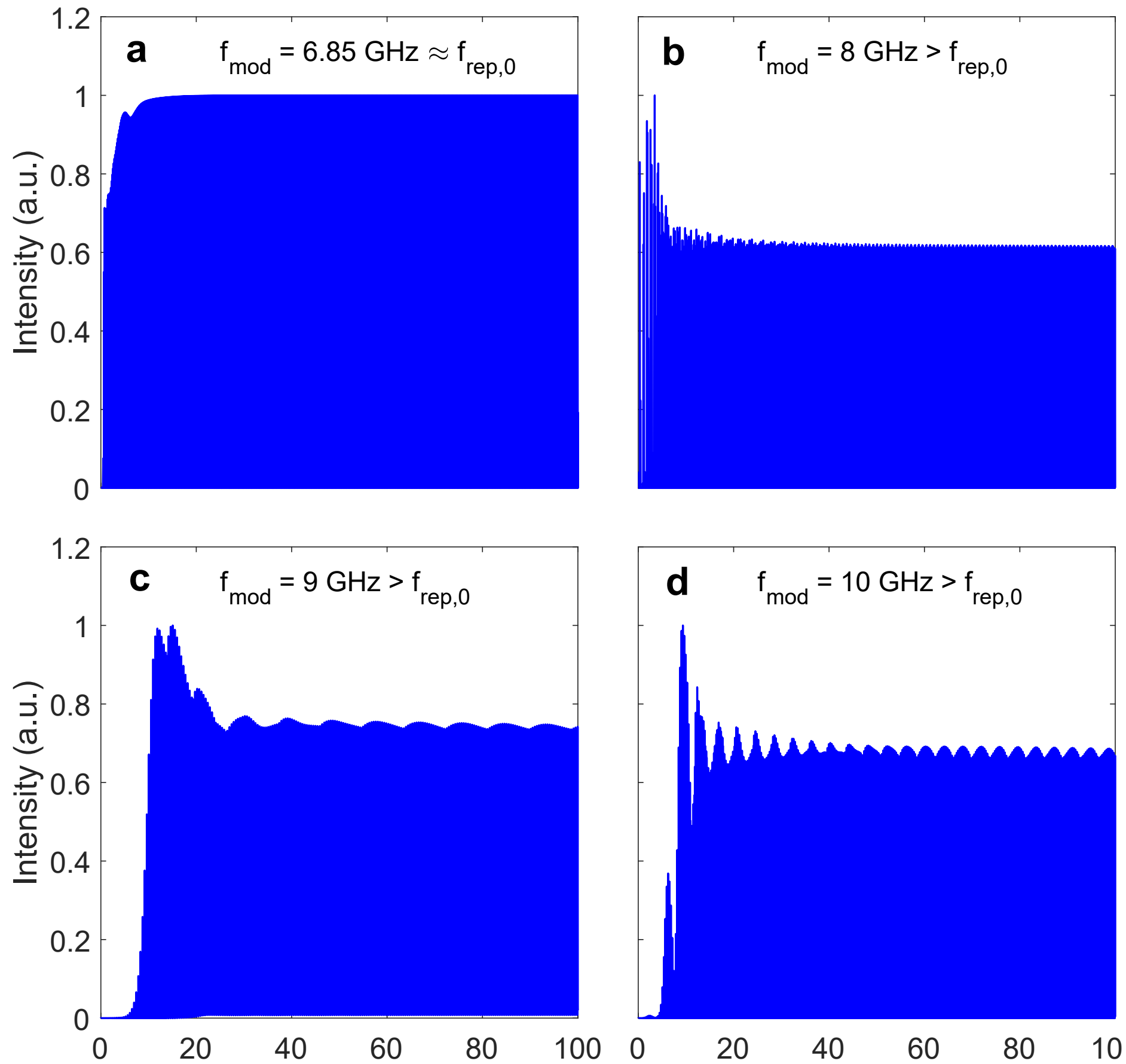

Figure S11: Comparison of the build-up dynamics for a variable modulation frequency.

S10.2 Single vs Double Pulse Regime

Our experiments and simulations indicate that the laser internally switches to a double pulse intracavity state already below $2 \times f_{\text{rep},0}$, while the repetition rate tuning is still fully continuous for any frequency above the low-frequency modulation limit of 3.5 GHz, as verified in experiments (with a fine frequency step) as well as in numerical simulations.

We performed additional numerical simulations to study this in greater detail. As shown in Extended Data Fig. 8, we can observe the switching to a double pulse at a modulation frequency of 12.8 GHz, which is below the second harmonic. There is a significant intermittent field already at 12 GHz, but in this case, it is still part of the single pulse, as indicated by the red arrow in panel b. At 12.8 GHz, these intermittent fields break off into separate pulses, generating a double pulse structure, as shown in panel c. It is important to note that, in terms of the intracavity field dynamics, these two cases are quite distinct. For example, for a modulation frequency of $f_{\text{mod}} = 1.8 \times f_{\text{rep},0}$, there is either a single pulse traveling with an increased group velocity

of $v_g = 1.8 \times v_{g,0}$, or two pulses traveling with a reduced group velocity of $v_g = 0.9 \times v_{g,0}$. Whether the state will consist of a single fast pulse or a double slow pulse structure depends essentially on which state is energetically more favorable to the laser operation. Additionally, with an increasing modulation frequency, the microwave field distribution features an increasing number of maxima and minima along the cavity length, also contributing to the generation of several intracavity pulses (see also the simulated microwave field distribution as a function of frequency in Fig. 2c of the main text). Based on our numerical simulations, the transition to a double intracavity pulse regime happens already well below the second harmonic modulation frequency (at around $f_{\mathrm{mod}} = 1.85 \times f_{\mathrm{rep},0}$).

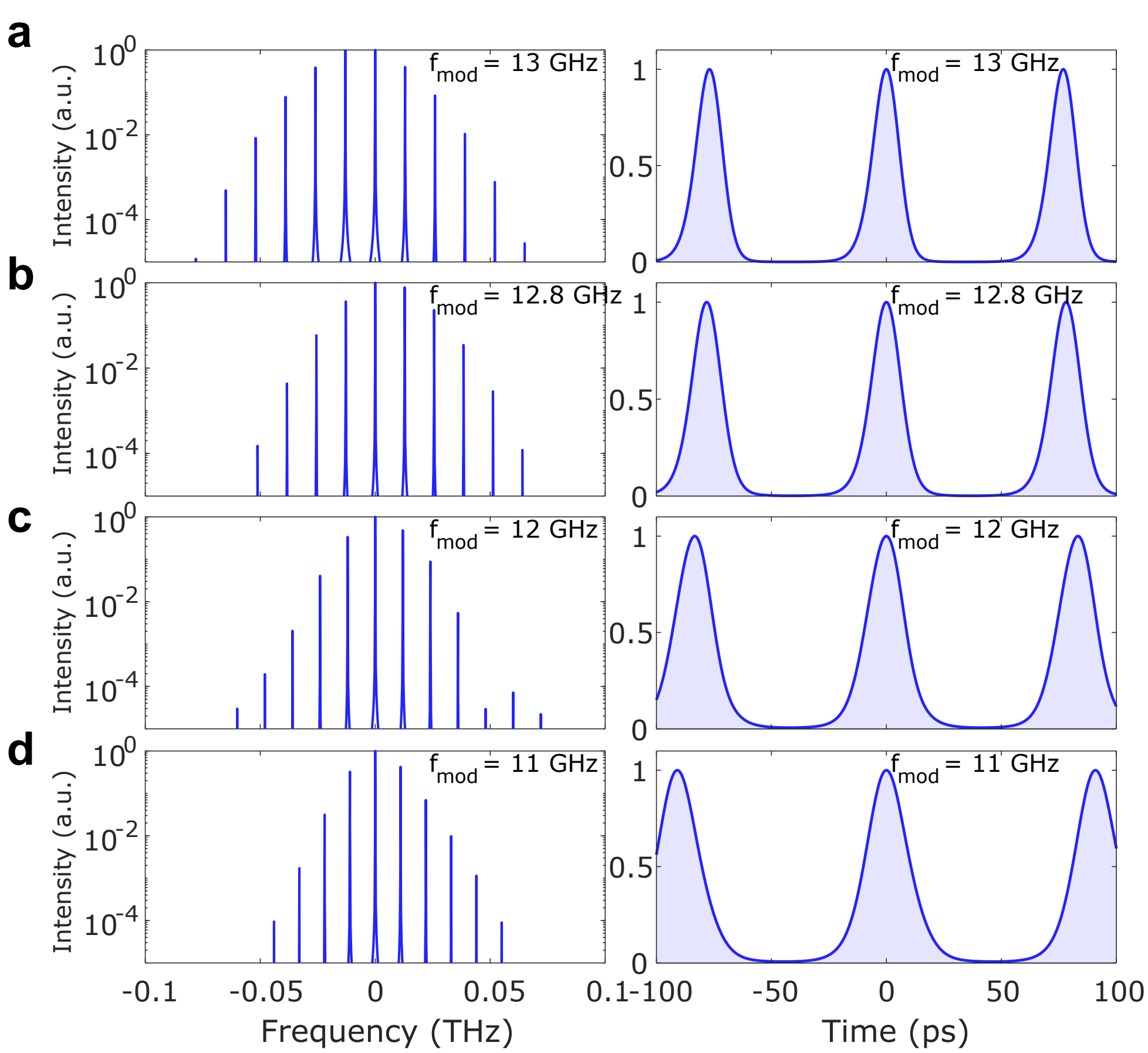

Figure S12: Numerically simulated output spectra and pulse trains display repetition rates exactly matched to the modulation frequency, without any clear spectral or temporal signatures differentiating between the single and double intracavity pulse regimes.

Nevertheless, in terms of the output intensity that is observable in experiments and important for applications, there is always a coherent pulse train with a repetition frequency that exactly matches the driving modulation frequency f_{mod}. Regardless of being in the single or double

intracavity pulse regime, the output pulses are always well isolated and show no traces of intermittent field components. The output pulses can also be visualized in Extended Data Fig. 8 as a line slice at the cavity position of 6 mm, indicating the location of the output facet. Even though the transition between the single and double pulse regimes can occur abruptly as we increase the modulation frequency, the repetition rate tuning is still fully continuous and does not disrupt the mode-locked operation. This is also evident in Fig. S12, where there are no clear spectral or temporal signatures in the output emission spectra and pulse trains that would indicate a difference between the operation in the single and double intracavity pulse regimes.

S10.3 Dependence on the DC Operating Point

To investigate the dependence of the detuned dynamics on the operating point, we performed a set of simulations with varying waveguide losses a for the off-resonant injection case of f_{mod} = 10 GHz. For all simulations, the same modulation strength is used. Figure S13 displays the last 800 ps of the resulting time domain data with the corresponding intensity spectra from a total simulation time of roughly 1.7 μs. As in the experiment, for higher operating points above threshold, the waveform of the outcoupled field deviates more strongly from the periodic waveform shown in the main text, corresponding to a non-locked state of the laser.

S10.4 Dependence on the Modulation Strength

Additionally, we investigate the influence of the modulation strength on the outcoupled pulses. To this end, we carried out simulations with two different modulation strengths, shown in Fig. S14. In these simulations, the modulation frequency is set to $f_{\mathrm{mod}} = 10$ GHz. While the blue line (corresponding to the stronger modulation) shows a sequence of distinct pulses, for the orange line (corresponding to the weaker modulation) the pulses are longer and not entirely separated.

S10.5 Increased Microwave Attenuation

We show the advantage of strong RF modulation along the whole waveguide, by increasing the damping coefficient of the microwave to a_{m}= 1000 m^{-1} = 4.3 $\mathrm{dB/mm}$ in a simulation. In this case, the RF wave remains unchanged at the injection point but experiences a strong attenuation along the waveguide. The resulting optical intensities in the frequency and time domain are shown in Fig. S15 after a simulation time of roughly 1.7 μs. Overall, the spectrum appears noisy and no sign of the frequency tunability is visible. Furthermore, the waveform in the time domain does not constitute a periodic pulse train, but rather exhibits an increased jitter, corresponding to an unlocked state of the device.

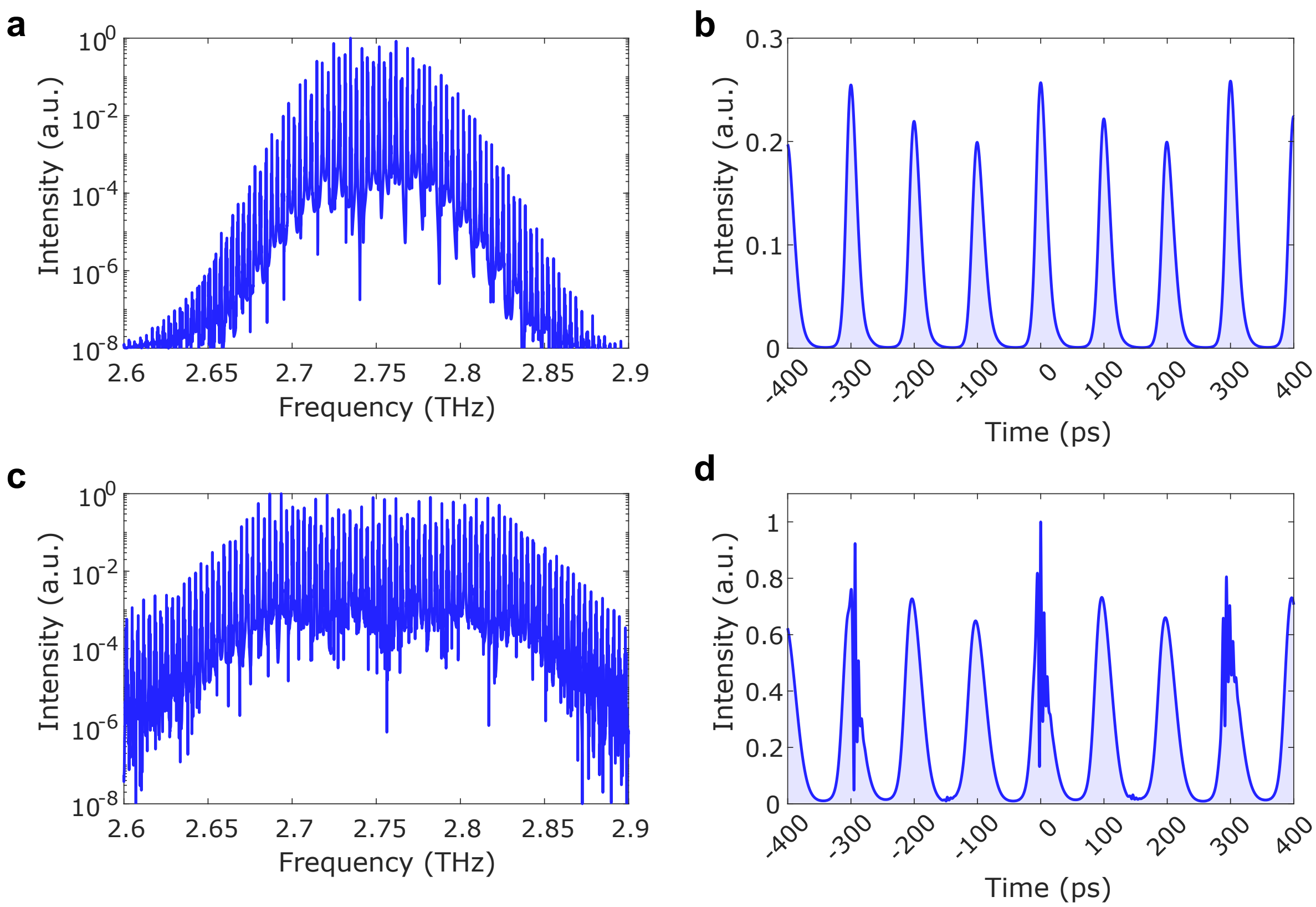

Figure S13: Simulation results with varying waveguide losses, corresponding to shifts of the operating point with respect to the threshold. While the intensity spectra are shown in the left column, the right column displays the corresponding time domain data. For the waveguide loss we use $a = 2000\ \mathrm{m}^{-1}$ in **a** and **b**, and $a = 1700\ \mathrm{m}^{-1}$ in **c** and **d**.

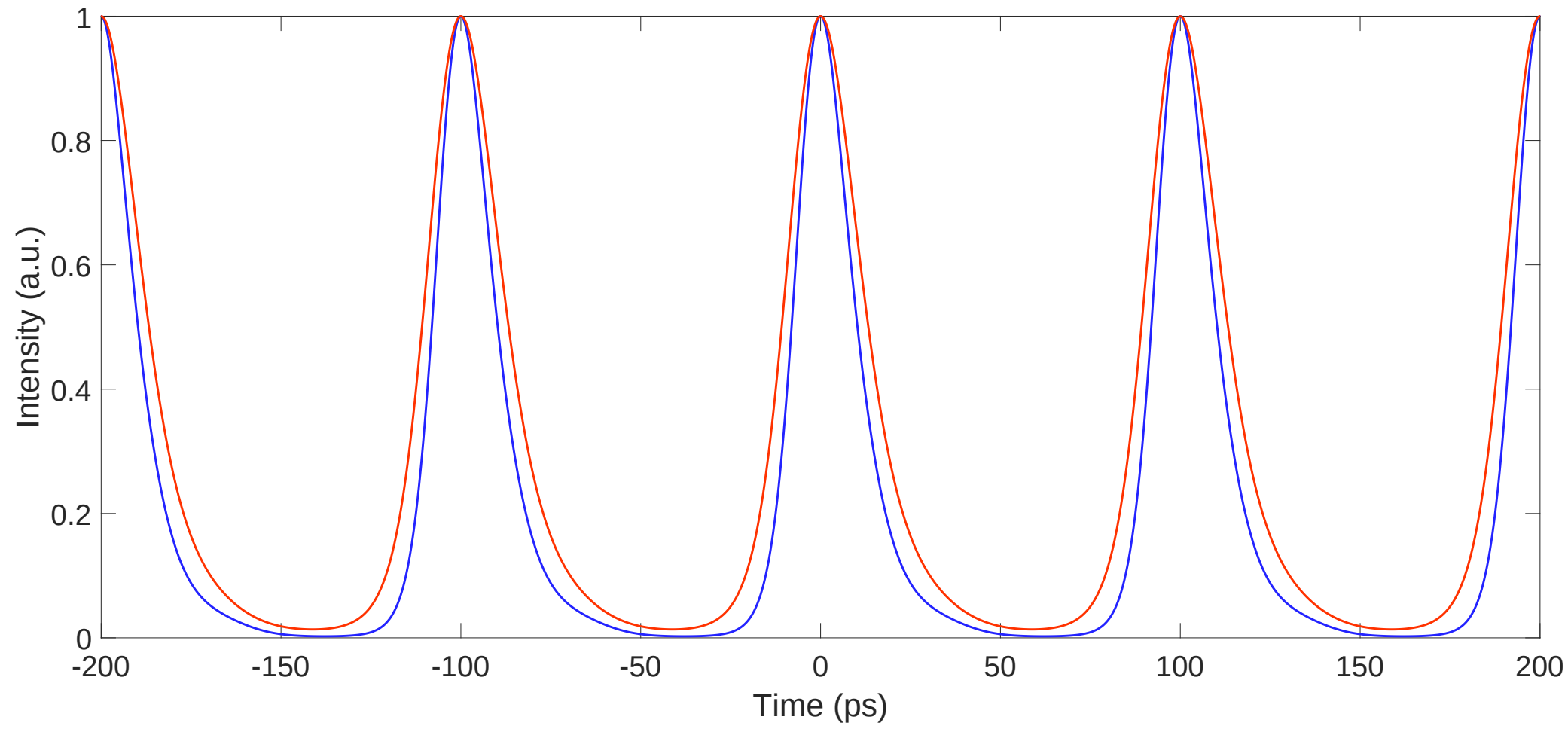

Figure S14: Simulation results for varying modulation strengths. The blue line corresponds to a modulation bias of 0.325 kV/cm, while the orange curve belongs to a modulation strength of 0.25 kV/cm. Clearly, a pulse shortening is obtained for stronger modulation.

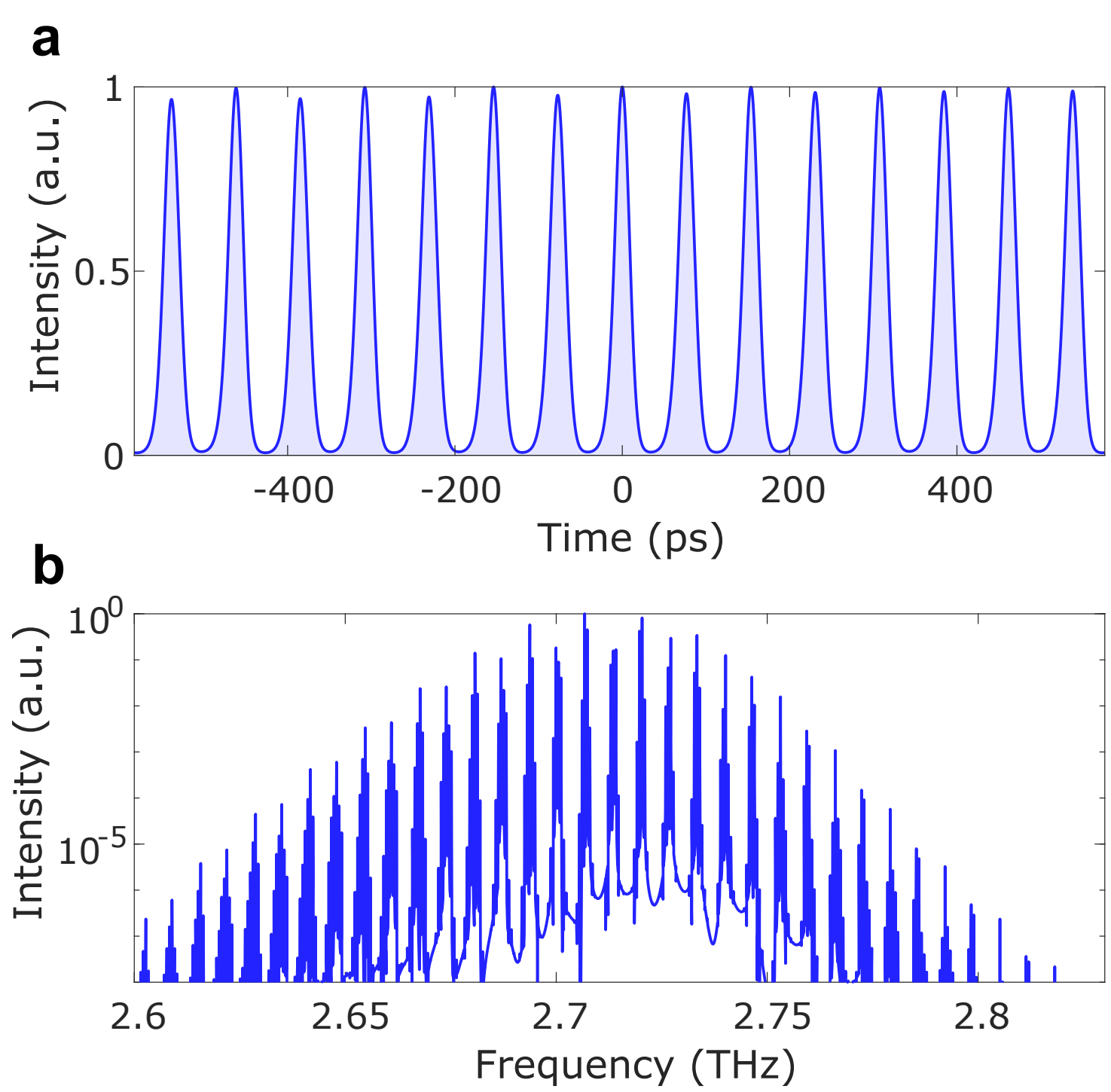

Figure S15: Simulation results for increased microwave losses. **a** Out-coupled intensity as a function of time and **b** corresponding intensity spectrum.

References

[1] Senica, U. *et al.* Planarized THz quantum cascade lasers for broadband coherent photonics. *Light: Science & Applications* **11**, 347 (2022).

[2] Hillbrand, J., Andrews, A. M., Detz, H., Strasser, G. & Schwarz, B. Coherent injection locking of quantum cascade laser frequency combs. *Nature Photonics* **13**, 101–104 (2019).

[3] Schneider, B. *et al.* Controlling quantum cascade laser optical frequency combs through microwave injection. *Laser & Photonics Reviews* **15**, 2100242 (2021).

[4] Kuizenga, D. & Siegman, A. FM and AM mode locking of the homogeneous laser-Part I: Theory. *IEEE Journal of Quantum Electronics* **6**, 694–708 (1970).

[5] Haus, H. A theory of forced mode locking. *IEEE Journal of Quantum Electronics* **11**, 323–330 (1975).

[6] Vladimirov, A. G. & Turaev, D. Model for passive mode locking in semiconductor lasers. *Physical Review A—Atomic, Molecular, and Optical Physics* **72**, 033808 (2005).

[7] Täschler, P., Miller, L., Kapsalidis, F., Beck, M. & Faist, J. Short pulses from a gain-switched quantum cascade laser. *Optica* **10**, 507–512 (2023).

[8] Cargioli, A. *et al.* Quantum cascade lasers as broadband sources via strong RF modulation. *APL Photonics* **9**, 036110 (2024).

[9] Silvestri, C., Columbo, L. L., Brambilla, M. & Gioannini, M. Coherent multi-mode dynamics in a quantum cascade laser: amplitude-and frequency-modulated optical frequency combs. *Optics Express* **28**, 23846–23861 (2020).

[10] Gellie, P. *et al.* Injection-locking of terahertz quantum cascade lasers up to 35GHz using RF amplitude modulation. *Optics Express* **18**, 20799–20816 (2010).

[11] Maineult, W. *et al.* Microwave modulation of terahertz quantum cascade lasers: a transmission-line approach. *Applied Physics Letters* **96** (2010).

[12] Silvestri, C., Qi, X., Taimre, T. & Rakić, A. D. Harmonic active mode locking in terahertz quantum cascade lasers. *Physical Review A* **108**, 013501 (2023).

[13] Senica, U. *et al.* Frequency-modulated combs via field-enhancing tapered waveguides. *Laser & Photonics Reviews* **17**, 2300472 (2023).

[14] Razeghi, M., Bai, Y. & Wang, F. High-power, high-wall-plug-efficiency quantum cascade lasers with high-brightness in continuous wave operation at 3–300μm. *Light: Science & Applications* **14**, 252 (2025).

[15] Senica, U. *et al.* Broadband surface-emitting THz laser frequency combs with inverse-designed integrated reflectors. *APL Photonics* **8**, 096101 (2023).

[16] Li, Y.-Y. *et al.* High power-efficiency terahertz quantum cascade laser. *Chinese Physics B* **25**, 084206 (2016).
