# Supplementary material for: Continuously tunable coherent pulse generation in a semiconductor laser
Source: Nature. 2026 Apr 15;652(8111):892–8. doi: 10.1038/s41586-026-10387-w (PMC13661238; doi:10.1038/s41586-026-10387-w)
Supplement: Supplementary file 2 [file 41586_2026_10387_MOESM6_ESM.pdf]

nature portfolio

Peer Review File

Continuously tunable coherent pulse generation in a semiconductor laser

Corresponding Author: Dr Urban Senica

Any redactions in this file are there to maintain patient confidentiality, the confidentiality of unpublished data, or to remove third-party material.

This file contains all reviewer reports in order by version, followed by all author rebuttals in order by version.

Version 0:

Reviewer comments:

Referee #1

(Remarks to the Author)
Comments on “Continuously tunable coherent pulse generation in semiconductor lasers”
Senica et al report in this work the experimental demonstration of a THz mode-locked QCL with distributed gain modulation, which enables continuously repetition rate tuning without changing the cavity geometric dimension.
The key performance of such laser is the continuously tunability over a wide-range of repetition rate, while the key underlying mechanism is the pulse pulling effect through imbalanced gain when the modulation frequency is detuned from the free-running resonant frequencies. The tunable comb structures and their locked phase relations are confirmed using the SWIFT spectroscopy measurement, while a series of parameter dependencies and dynamic features of such tunable laser is investigated based on numerical models.
This work present systematic measurements and simulations results that clearly shows the crucial pulse pulling mechanism and the resultant variable pulse group velocity (i.e. tunable repetition rate), offering a novel manipulation regime for the well-known mode-locking technology, which may attract wide interests in this broad research field.
The experimental and simulation methods used in this experiment are valid and widely used. The manuscript is clearly written and easy to read.
Therefore, I would like to recommend this manuscript for publication in Nature, given that the following issues are addressed properly.
1. When the gain modulation frequency is detuned from the resonant frequency of the cavity, the gain slope would induce pulse pulling effect in order to drag the pulse along with the propagating gain window. How is the fundamental mode-locking condition is still fulfilled in such detuned case, such that the pulse profile (close to transform-limit) is maintained while the background noise is fully suppressed? (since in this case the pulse is no longer stably confined within the gain window)
2. The pulse pulling effect induced by the gain modulation given significant detuning from the free-running resonant frequency seem to introduce significant (although transient) attenuation within a round-trip evolution (e.g. Fig.5(b)) (a strong energy “breathing”). What is maximum attenuation allowed for a coherent pulse evolution (instead of being in the gain switching regime) in such detuned case? In addition, how the pulse energy breathing ratio related to the frequency detuning?
3. The author claims that repetition rate tuning rate is limited by the comb stabilization time, which is on the order of ~1us. What determines the length of this time? In addition, Fig.S10 in the supplement gives a simulated build up time of only a few tens of nanosecond. Why is this stabilization time significantly longer than the build-up time?
4. The induced microwave in top and bottom metallic layer is strongly reflected at the emitting facet of the cavity to create desired modulations. Need the facet on the other side (where the microwave is launched) to be strongly absorptive for the microwave in order to achieved the desired distributed modulation?
5. It can be noticed that when the modulation frequency approached the 2nd harmonic, the gaps between adjacent pulses is filled with non-trivial residual field, as least within a certain range of position during each round trip (Fig.5c). Meanwhile the laser switched to double-pulse state given a modulation above the 2nd harmonic of the cavity resonant frequency (Fig.5d). I wonder if such switching to N=2 occurs as the intermittent field between the pulses arise as secondary pulses? It the repetition rate tuning still continuously during such switching of harmonic order ?
6. The author claims that the tunable comb spacing can be used for high resolution gapless spectroscopy. However, the spectral envelop profile changed dramatically when the comb spacing is tuned (Fig.S2). In addition, the central comb

position also seems to change under different modulation frequency. These features may make it difficult to use since the comb profile needs to be calibrated for each different modulation frequency. The authors are expected to address these concerns for this application.

Referee #2

(Remarks to the Author)
The authors report on a novel modelocking regime in which coherent pulses can be generated with frequency spacings that are not defined by the physical cavity length in a monolithic geometry. This is possible because a sufficiently strong radio frequency modulation of the (semiconductor) gain medium detuned from the natural cavity resonance results in strong pulse shaping effects that change the group velocity of the pulse allowing to tune the pulse time spacing to an arbitrary modulation frequency. The manuscript presents experimental data using a THz quantum cascade laser as a test system for this idea and demonstrate continuous tuning of the pulse repetition rate from 4 to 16 GHz – both with lower and higher modulation than the natural cavity repetition rate of 6.6 GHz. They present both a simple analytical model based on the traditional Haus formalism for modelocking to explain the mechanism observed and numerical simulations for the specific experimental setup to confirm the observed behavior and parameters.

To the best of my knowledge, it is the first time such a continuous tunability of the repetition rate is demonstrated with a monolithic platform. The basic physics underlying the principles presented here have been treated theoretically long ago as well cited by the authors in particular in [17], but so far detuning the modulation frequency in active modelocking was rather seen as a source of instability and a curiosity, rather than an effect that can lead to useful cavity dynamics. In that regard, this manuscript presents an original, new point of view to an 'old' problem. The significance of the result is on rethinking active modelocking dynamics that could spark interest. In my opinion, this is the strongest point of interest of the manuscript. Concerning significance: modelocked lasers are tremendously important technologically for many fields and on-chip coherent pulse generation, in particular with tunability in a restrained geometry, are a big challenge. Having said this, I am not fully convinced of the immediate significance of this technology in its current state of performance. It may be possible, that some points could be better generalized and clarified in the manuscript to address these points:

• It seems that this new regime can only be stable in a rather niche technology case for semiconductor lasers with rather long pulses: from the analysis performed it seems these pulling time effects will only be effective for 'cleanly' shaping the group velocity if the pulses are long and dispersion and nonlinear effects can be neglected. I believe there is some analysis missing on whether this mechanism could be sustained in the case of more broadband, shorter pulse durations where effects such as dispersion, self-phase modulation, spectral filtering (and the resulting time clipping) etc, would be present and cannot be neglected. This would give the claims more generality for other laser types and would also broaden the application spectrum of such integrated lasers in general.
• The main application put forward is that of frequency comb spectroscopy. Whereas this seems appropriately identified since tunable repetition rate would allow to resolve features 'missed' or poorly resolved in static repetition rate spectroscopic experiments (in particular at very high repetition rates), it would seem that the point above of the rather long pulse duration and rather narrowband operation is a strong limitation for this particular application. Can this concept be combined with central frequency tunability on chip?
• In the supplementary material, Fig. S15 indicates that the central wavelength but also the overall bandwidth significantly changes while the repetition rate is tuned. The tapered device Fig. S 16 performs better and possibly could be more practically used for spectroscopy, however it is not clear why it is not possible to perform a proof of principle experiment of absorption spectroscopy using this comb system. It would make the case of the usefulness of the devices much stronger.
• Generally, parameters vary significantly when tuning the repetition rate, which makes the tunability aspect somehow questionable in general. It would be useful for the reader to present a more detailed path forward to have tunability without significant changes in other output parameters.
• As pointed several times by the authors, effective spatio-temporal gain modulation is only effective when operating the laser close to threshold, but threshold operation is often associated with noisy operation regimes. It would be important to comment on this point in particular on the linewidth of the obtained combs, when comparing to other comb technologies. In the supplementary material, there seems to be some variability in the linewidth as well in the different operation points.
• This technique allows to tune the repetition rate, but it is unclear how is the carrier envelope offset frequency stability affected by operating in this regime. This deserves a more detailed discussion as well.

With all the parameter limitations as correctly identified by the authors that limit also the tunability range in practice, I ask myself if this technique and the technology has really the potential to surpass more complex, but more standard, techniques to tune the repetition rate in integrated geometries – perhaps not fully continuously. For example Mach Zender interferometers have been shown in integrated platforms to provide a lot of parameter flexibility for repetition rate multiplication. It would also be good to refer to the special advantages of this technique comparing with external techniques as well.

I would also like to refer the authors to the case of random fiber lasers, where I believe such spatio temporal gain dynamics are used for similar cases of 'mode-less' cavities. I believe it makes sense to draw some parallels to this technology as well. For example: von der Weid, J.P., Correia, M.M., Tovar, P. et al. A mode-locked random laser generating transform-limited optical pulses. Nat Commun 15, 177 (2024) for one recent publication in this area.

The data and methodology used in the paper are of excellent quality as well as the presentation of the results. The conclusions are valid and relevant work is appropriately cited to the best of my knowledge – with some suggestions above to expand the scope.

Concerning the overall clarity of the manuscript it would be useful for a general audience to have a more general introduction presenting also other comb technologies and their capabilities (not only in terms of repetition rate tunability, but other parameters of relevance) to be able to place the results in proper context, and how and in which cases this new method promises to outperform other integrated technologies. This may also be appropriate in the conclusion section, as the experimental results have already been presented.

Referee #3

(Remarks to the Author)
The manuscript "Continuously tunable coherent pulse generation in semiconductor lasers" by Urban Senica et. al reported a monolithic semiconductor laser with a continuously tunable repetition rate from 4~16 GHz. The authors proposed a novel regime, where the repetition rate of a mode-locked semiconductor laser can be tuned continuously and significantly both above and below the fundamental frequency defined by the cavity length. The presented results are encouraging and novel. But I do have some major concerns in terms of the practical applications of their work. On one hand, the modulation amplitude needs to be large enough for the spatiotemporal gain modulation to generate the tunable coherent pulse, for example, the RF powers are all above 30 dBm in the manuscript. This may severely limit their applications in the claimed fields such as sensing or telecommunications. On the other hand, the authors describe in the manuscript and in Supplementary Material that the measurements are operated inside a shield under low vacuum conditions of 10-6 mbar and cooled temperatures ~40 K, I wonder why such extreme operating conditions are needed? As far as I know, the THz QCLs can be operated in >150 K. If such extreme conditions are necessary, it may also severely limit their usage. It would be beneficial to provide some insights on what conditions are needed for applying this concept to widely used semiconductor lasers such as DFBs, FPs, etc.

Some minor comments are given below:
1. The authors provide limited info about the sample, for example, 6 mm long, 40 µm wide homogeneous ridge. More details would be helpful, such as specific materials and dimensions of waveguide, metals, III-V substrate, the waveguide loss, the reflection coefficient of waveguide facets, heat dissipation, microwave properties, etc.

2. What is the photoelectric conversion efficiency of the sample? Is there any difference between the photoelectric conversion efficiencies of resonant frequency, harmonics and other frequencies?

3. The authors mentioned that the high modulation frequencies are in principle high, provided that the modulation amplitudes are large, the microwave losses are low, and the modulation frequency is lower than the laser gain bandwidth. How large is the amplitude? How small is microwave loss? And how broad is the gain bandwidth? Theoretically, is there an exact modulation frequency limit?

4. The spatiotemporal gain modulation is implemented by injecting microwaves into waveguide to generate a standing wave, does this mean that the generated pulse train is still constrained by the FSR and the tuning is not strictly continuous?

5. Is it possible for the authors to apply similar concepts to the widely used rare earth doped fiber lasers? If so, what would be the conditions?

6. In the comb properties and generalizations section, all the figures are in the Supplementary Material. The authors might consider moving some figures to the main text.

Version 1:

Reviewer comments:

Referee #1

(Remarks to the Author)
Senica et al have satisfactorily addressed all the issues raised in my previous comments and I have no more questions to raise. Nevertheless, I do have one suggestion for the authors to consider concerning the first two questions raised in my previous comments (one concerns the mode-locking stability and the other concerns the energy breathing ratio). Since the manuscript is mainly on a novel mode-locking mechanism, these two aspects of ultrafast laser dynamics would be worth mentioning in the main text with brief discussion of the phenomena and the underlying mechanism rather than being only in the supplementary material.

Referee #2

(Remarks to the Author)
I believe the manuscript with its modifications really have improved and I support publication. The change in the title and the additions in the main text more accurately capture the essence of the result and clarify the scope compared also to other technoloogies. The extra supplementary material is very helpful for a wide audience.
I understood better the implications thanks to the answers to the comments, and appreciate the great effort put in answering the questions.

In particular the simple spectroscopy experiment strengthens the case of possible 'usefulness' in spit of the parameters still being not perfectly in the state of the art in absolute values. Although it is still a simple 'proof of concept' it is already meaningful given the sources are a new class of sources.

Referee #3

(Remarks to the Author)
All my previous concerns have been adequately addressed by the authors. Therefore, I recommend the manuscript for publication.

Open Access This Peer Review File is licensed under a Creative Commons Attribution 4.0 International License, which permits use, sharing, adaptation, distribution and reproduction in any medium or format, as long as you give appropriate credit to the original author(s) and the source, provide a link to the Creative Commons license, and indicate if changes were made.
In cases where reviewers are anonymous, credit should be given to 'Anonymous Referee' and the source.
The images or other third party material in this Peer Review File are included in the article’s Creative Commons license, unless indicated otherwise in a credit line to the material. If material is not included in the article’s Creative Commons license and your intended use is not permitted by statutory regulation or exceeds the permitted use, you will need to obtain permission directly from the copyright holder.
To view a copy of this license, visit https://creativecommons.org/licenses/by/4.0/

Notes on revision made to manuscript
Continuously tunable coherent pulse generation in semiconductor lasers

The authors would like to thank the Editor and Reviewers for their constructive comments and suggestions that have helped improve the quality of this manuscript. The manuscript has undergone a thorough revision according to the Editor's and Reviewers' feedback. Please see below our responses. For the Reviewers' convenience, our replies are in **blue**, and we have highlighted all the significant changes in the revised manuscript.

Major Changes

In this section, we would first like to highlight some major changes to the manuscript, which are relevant to all the Reviewers and the Editor.

1. **Updated title**
   We have updated the manuscript title to "Continuously and widely tunable coherent pulse generation in semiconductor lasers", as we believe this more accurately captures the essence of our reported research work.

2. **New co-author**
   A new co-author (Marco Raffa) has been added to the author list, as he performed significant additional experimental work after the first author (Urban Senica) moved to another university.

3. **Main Text & Supplementary Information**
   Due to reaching the limit of the text length and the number of figures in the Main Text, as instructed by the Editor, parts of the Main Text had to be reported in a more compact form or moved to Methods or Supplementary Information. These have also been expanded to include answers to Reviewers' questions and suggestions. The Methods section has been moved to the end of the same document as the Main Text.

4. **New experiment**
   The most significant update has been the development of an absorption spectroscopy experiment with our tunable comb laser. While more details can be found in the Main Text and Methods, we provide a short summary here below.

As shown in the illustration in Fig. 1(a) (new Fig. 5 in the Main Text), the compact experimental setup consists of only a few elements. The tunable comb laser emission shines on a sample under test with characteristic absorption features within the emission spectrum. By tuning the RF modulation frequency, the emission spectrum is swept, and the detector intensity is recorded. The absorption spectrum is then reconstructed using an algorithm to solve the matrix system of equations $\mathbf{b} = \mathbf{M}\mathbf{x}$, where $\mathbf{b}$ contains the measured detector intensities, $\mathbf{M}$ the comb laser emission spectra, and $\mathbf{x}$ the frequency-dependent absorption of the sample, each describing its dependence on the modulation frequency and the optical frequency. The absorption profile can be retrieved

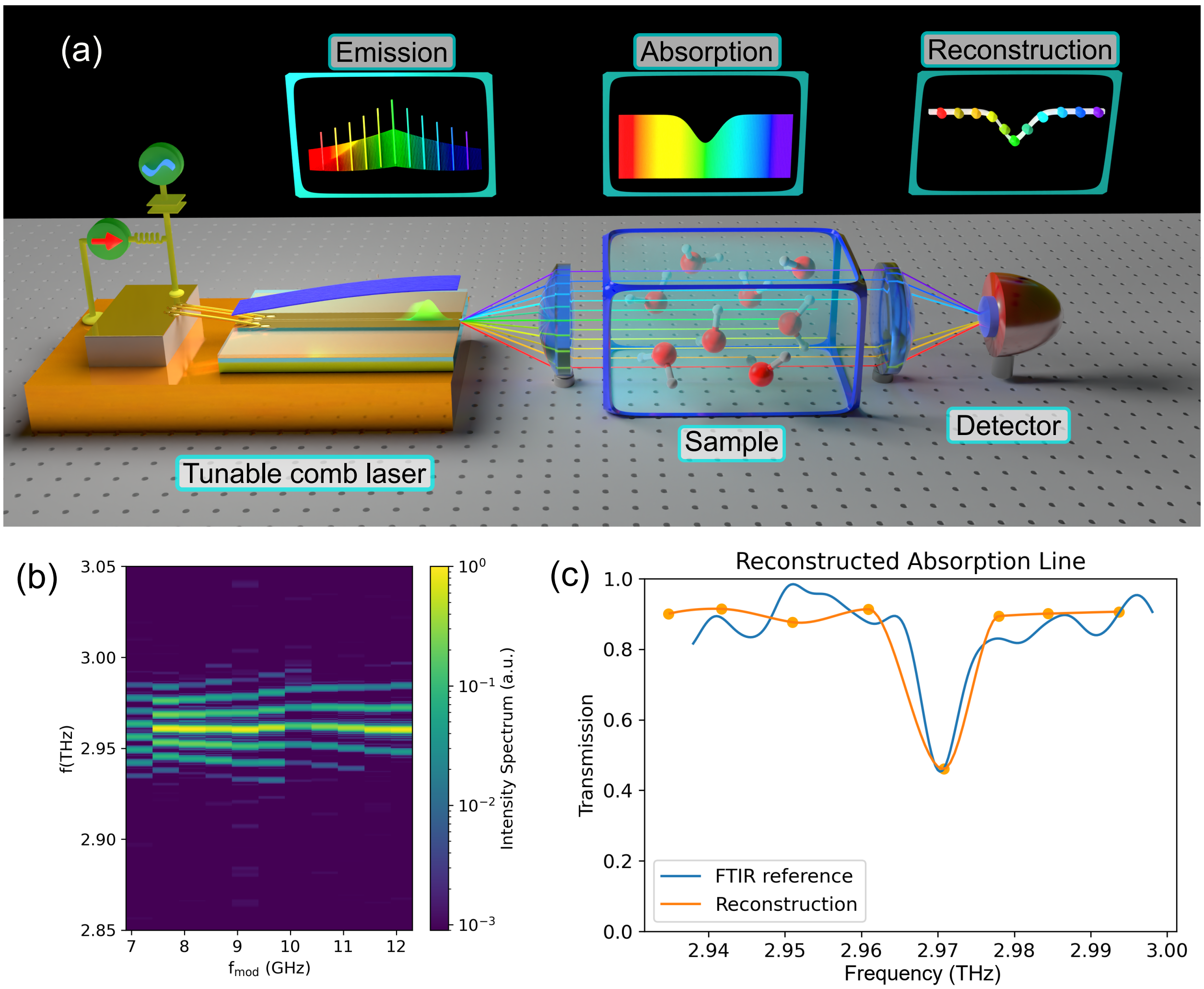

Figure 1: **Absorption spectroscopy.** **(a)** Illustration of the experimental setup, requiring only an intensity detector in combination with our tunable comb laser to investigate a sample under test. **(b)** Emission spectrum of the tunable comb as a function of modulation frequency. **(c)** The reconstructed absorption line (orange) shows excellent agreement with the reference FTIR spectrometer measurement (blue).

from a known laser source matrix $\mathbf{M}$ and the measured detector intensity as a function of the modulation frequency.

In the experiment, we used one of our tapered waveguide samples, where only the modulation frequency is swept, while all the other crucial parameters (DC laser bias, RF power, temperature, etc.) are stabilized at constant values. This produces a tunable comb spectrum with a relatively stable central emission frequency, as can be seen in Fig. 1(b). The investigated sample is water vapor with characteristic absorption peaks in the THz spectral range. In Fig. 1(b), we plot the reconstructed absorption line at 2.97 THz, and compare it with a reference measurement with an FTIR spectrometer. The two methods show good agreement in all the crucial aspects: the absorption resonance frequency, width, and depth.

This proof-of-principle experiment highlights the potential applicability of the repetition-rate frequency tunable comb laser in spectroscopy, featuring a very compact and simple experimental setup, requiring only the tunable comb laser, the sample under test, and an intensity detector. Moreover, by using a tapered waveguide device, only the modulation frequency is swept while all the other parameters are kept constant, resulting in a very straightforward, robust, and reliable measurement protocol for the end user.

Response to the reviewers

Reviewer 1

Comments on "Continuously tunable coherent pulse generation in semiconductor lasers"

Senica et al report in this work the experimental demonstration of a THz mode-locked QCL with distributed gain modulation, which enables continuously repetition rate tuning without changing the cavity geometric dimension. The key performance of such laser is the continuously tunability over a wide-range of repetition rate, while the key underlying mechanism is the pulse pulling effect through imbalanced gain when the modulation frequency is detuned from the free-running resonant frequencies. The tunable comb structures and their locked phase relations are confirmed using the SWIFT spectroscopy measurement, while a series of parameter dependencies and dynamic features of such tunable laser is investigated based on numerical models. This work present systematic measurements and simulations results that clearly shows the crucial pulse pulling mechanism and the resultant variable pulse group velocity (i.e. tunable repetition rate), offering a novel manipulation regime for the well-known mode-locking technology, which may attract wide interests in this broad research field. The experimental and simulation methods used in this experiment are valid and widely used. The manuscript is clearly written and easy to read. Therefore, I would like to recommend this manuscript for publication in Nature, given that the following issues are addressed properly.

Reviewer Comment 1.1 — 1. When the gain modulation frequency is detuned from the resonant frequency of the cavity, the gain slope would induce pulse pulling effect in order to drag the pulse along with the propagating gain window. How is the fundamental mode-locking condition is still fulfilled in such detuned case, such that the pulse profile (close to transform-limit) is maintained while the background noise is fully suppressed? (since in this case the pulse is no longer stably confined within the gain window)

Reply: The Reviewer correctly summarized the essence of the pulse pulling effect, i.e., the phase shift and the corresponding gain slope (or gradient) across the pulse results in a dragging along with the traveling net gain window, effectively changing the pulse group velocity, such that it is synchronized to the external microwave driving frequency. Indeed, even in the detuned case, the dragged pulse is actually still stably confined within the net gain window. In Kärtner's paper [18], a linear stability analysis even yields unconditional stability for detuned modulation. However, investigation of effects beyond this analysis (turbulence) gives some stability limit. For a more thorough discussion, we refer to F.X. Kärtner, "Ultrafast Optics", MIT OpenCourseWare, `https://ocw.mit.edu/courses/6-977-ultrafast-optics-spring-2005/pages/lecture-notes/` Chapter 5.7.

All those effects are intrinsically considered in our extensive numerical simulations. We note that in these simulations, also the effect of spontaneous emission can be considered through added numerical noise (see C. Jirauschek, "Dynamic modeling of quantum optoelectronic devices", Proc. 2023 IEEE Nanotechnology Materials and Devices Conference). Therefore, the stability of mode-locking with detuning in our system has been demonstrated and proven with several methods: using the simplified analytical model, employing extensive numerical simulations, and experimentally using SWIFT spectroscopy.

Reviewer Comment 1.2 — 2. The pulse pulling effect induced by the gain modulation given significant detuning from the free-running resonant frequency seem to introduce significant (although transient) attenuation within a round-trip evolution (e.g. Fig.5(b)) (a strong energy "breathing"). What is maximum attenuation allowed for a coherent pulse evolution (instead of being in the gain switching regime) in such detuned case? In addition, how the pulse energy breathing ratio related to the frequency detuning?

Reply: The Reviewer makes an excellent point. Indeed, with frequency detuning, there can be significant energy breathing throughout the roundtrip time. The breathing ratio is larger for very low modulation frequencies below the natural repetition rate ($f_{\mathrm{mod}} < f_{\mathrm{rep},0}$), and this is related to the fact that within a significant fraction of the roundtrip, the pulse propagation is actually going "against" the traveling gain modulation window (in the opposite direction) and being strongly attenuated (this can also clearly be seen in the Supplementary Movies). For higher frequencies, this effect is not so pronounced (as can be seen in the former Fig. 5(c,d), which is now Fig. 4(c,d)). At low frequencies, it is exactly this strong attenuation (breathing) that results in the laser going to the gain switching regime, which is an incoherent pulse stream.

Experimentally, we were able to demonstrate tunable mode-locked pulses down to 3.5 GHz, which is close to $f_{\mathrm{rep},0}/2 = 3.3$ GHz. There are two competing operation regimes, as shown in Fig. 2 (Fig. S10). The modulation amplitude needs to be large enough for the spatiotemporal gain modulation to generate the tunable coherent pulse, as shown in panel (a) for an RF injection power of +30 dBm. However, if the modulation power is further increased to +32 dBm, the laser enters a gain-switched regime (see also Täschler, et al., "Short pulses from a gain-switched quantum cascade laser", Optica 10, 507, 2023), producing broadband, low-coherence emission with a mode spacing of $f_{\mathrm{rep},0}/2$. In this case, the amplitude and duration of the negative RF half-cycle are so significant that the pulse gets fully attenuated (absorbed). In other words, it is energetically more favorable for the laser to enter a gain switching regime, where pulses are propagating with the normal group velocity, but are fully absorbed between each modulation period, with a new pulse generated each time, i.e., an incoherent pulse stream. Additionally, for very low modulation frequencies, we are approaching the quasi-static regime for the RF field (subwavelength dimensions with reduced RF field modulation along the cavity), another factor contributing to a smaller gain slope (see also the simulated RF field distributions in Fig. 2(c) of the Main Text).

Considering the combination of all these effects, this means that below the low-frequency limit, there is no suitable amplitude of the RF modulation field that would be simultaneously strong enough to generate tunable pulses and weak enough not to fully attenuate them within the slow modulation roundtrip time, hindering the formation of a coherent mode-locked state. We should note that this only happens at the low-frequency cut-off point, while for high modulation frequencies, the energy breathing effect is much smaller, since pulses are more cleanly co-propagating with the traveling gain window throughout the entire roundtrip time, without significant attenuation effects.

We have also expanded the discussion on this in the Supplementary Information, section S6.

Reviewer Comment 1.3 — 3. The author claims that repetition rate tuning rate is limited by the comb stabilization time, which is on the order of 1us. What determines the length of this time? In addition, Fig.S10 in the supplement gives a simulated build up time of only a few tens of nanosecond. Why is this stabilization time significantly longer than the build-up time?

Reply: The Reviewer is absolutely correct that the pulse/comb stabilization time is much longer than the laser build-up time, which is a common phenomenon for mode-locked lasers, see, e.g., F. X. Kärtner

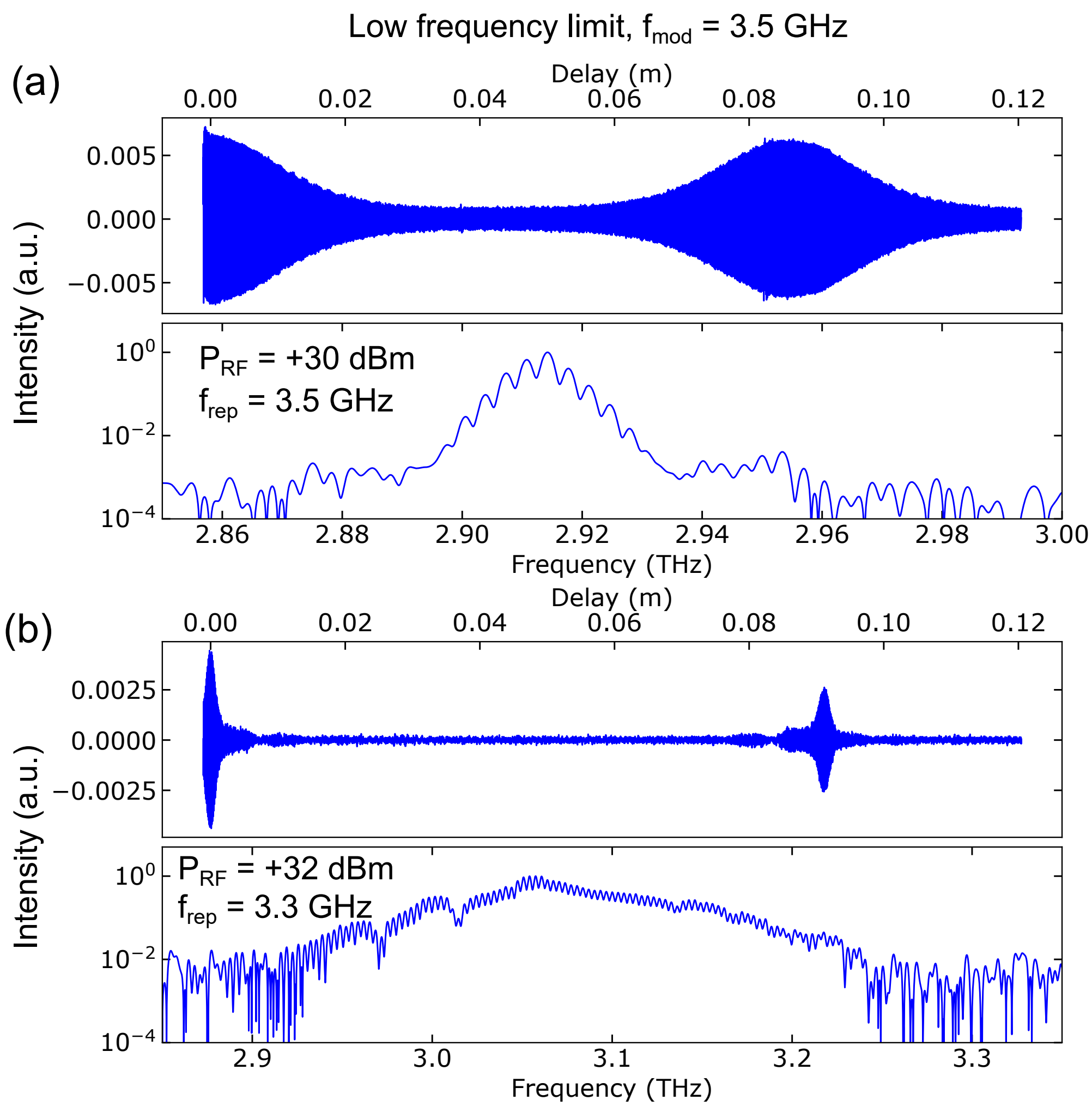

Figure 2: Measurement results at the experimentally-observed lower modulation frequency limit of 3.5 GHz, displaying in **(a)** the tunable coherent pulse state and in **(b)** the broadband, low-coherence gain-switched state with a mode spacing of $f_{rep,0}/2$, obtained for increased modulation amplitudes ($P_{RF} \geq +32$ dBm).

et al., J. Opt. Soc. Am. B 12 (1995). Specifically, for detuned lasers this effect has been thoroughly discussed in F. X. Kärtner, "Ultrafast Optics", MIT OpenCourseWare, `https://ocw.mit.edu/courses/6-977-ultrafast-optics-spring-2005/pages/lecture-notes/`, Chapter 5.7.

The comb stabilization time depends on several factors, e.g., it increases with the length of the cavity and with the amount of detuning from the resonant frequency. Typically, several thousand roundtrips are required to fully stabilize. We have performed a numerical simulation study of the comb stabilization for several driving frequencies, as shown in Fig. 3 (Fig. S18), where it is clear that the stabilization time increases with detuning further away from the cavity resonance.

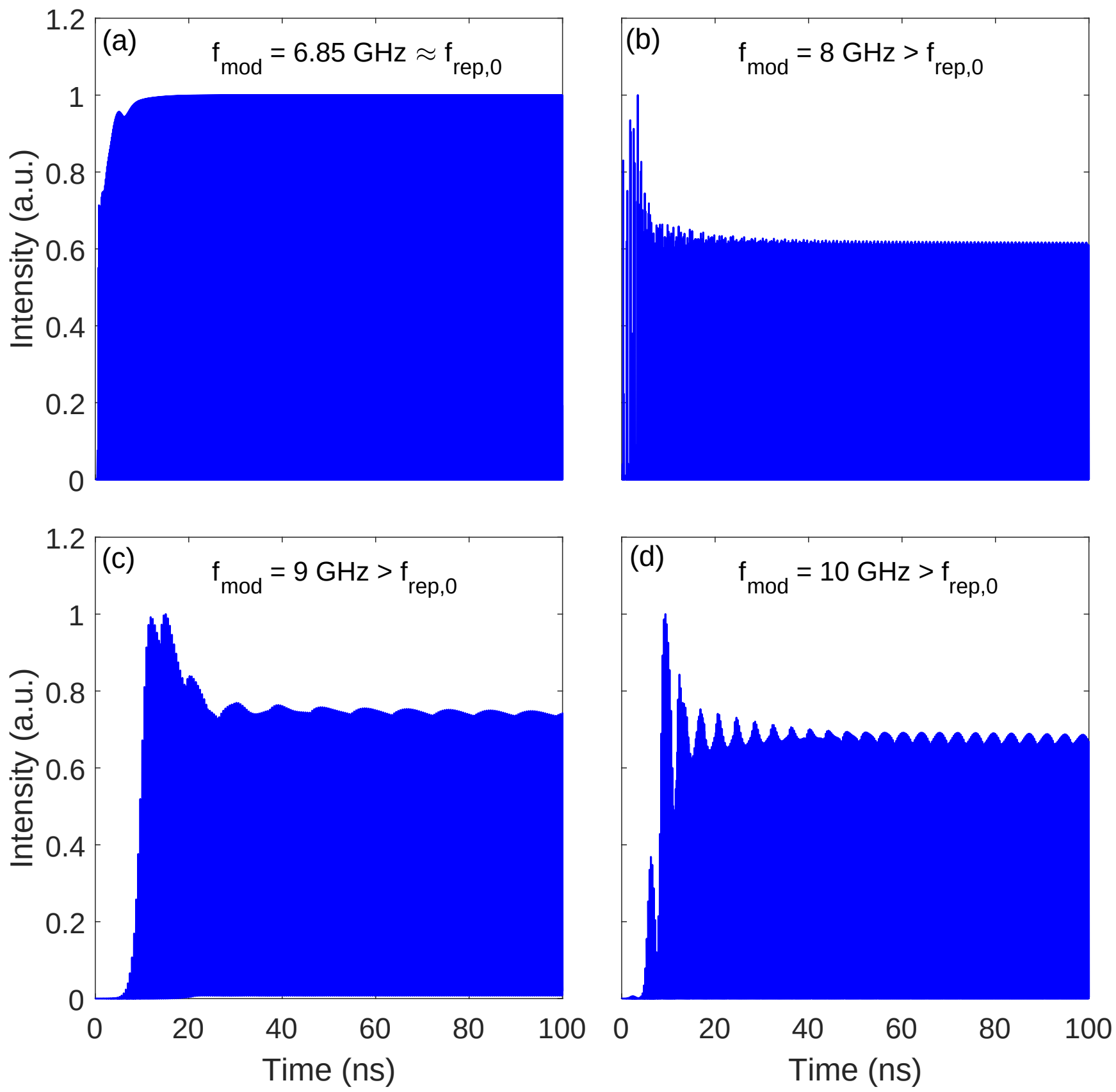

Figure 3: Comparison of the build-up dynamics for a variable modulation frequency.

In the original Fig. S10, we showed the zoomed-in simulation results of the first few hundred nanoseconds to highlight the transient behaviour. In the updated Fig. 4 (Fig. S17), we now show the full transient behaviour up to the first 1200 ns in panel (a). In this simulation, a modulation frequency of $f_{\mathrm{mod}} = 9$ GHz $> f_{\mathrm{rep},0}$ was applied. After an initial fluctuation of the intensity, the system converges to a locked state with constant pulse maxima. Figures 4 (b) and (c) depict two spectra recorded at $t = 200$ ns and $t = 1180$ ns, respectively. The corresponding time intervals used for the Fourier transformations are highlighted in the time trace shown in Fig 4 (a), using color-coded segments. While the first spectrum still exhibits side modes, the second spectrum shows a clean frequency comb with a mode spacing following the modulation frequency. Accordingly, a narrow RF beatnote at the modulation frequency and its higher harmonics is obtained for the steady-state case, as can be seen in the inset of

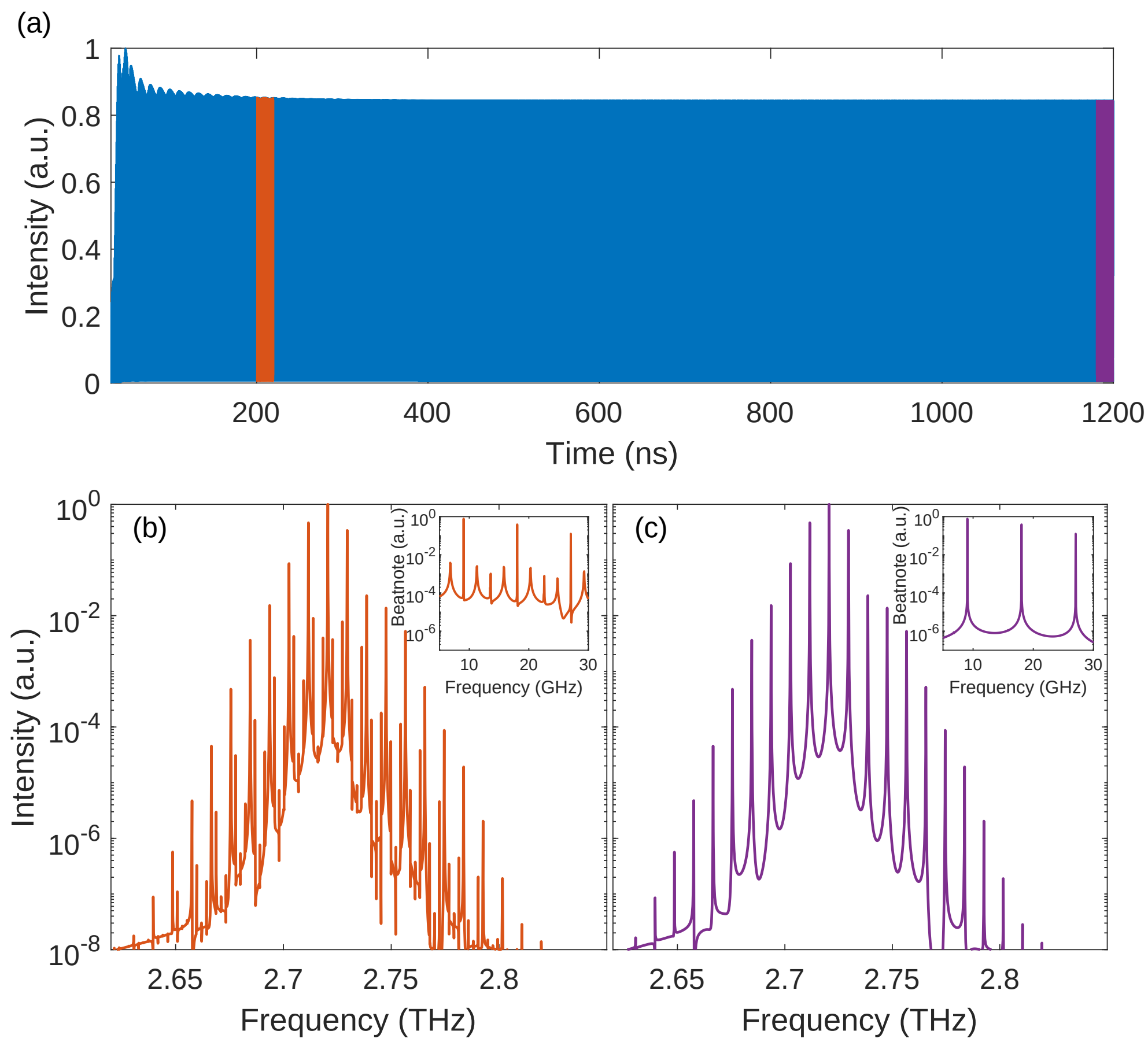

Figure 4: Simulation results, showing the temporal build-up of the tunable frequency comb. **(a)** Out-coupled intensity over time. **(b)** Intensity spectrum, recorded after 200 ns. **(c)** Intensity spectrum, recorded after 1180 ns. The insets show the RF beatnotes of the respective spectra.

Fig. 4 (c).

The new/updated Figures and text were added to the Supplementary Information, section S10.1.

Reviewer Comment 1.4 — 4. The induced microwave in top and bottom metallic layer is strongly reflected at the emitting facet of the cavity to create desired modulations. Need the facet on the other side (where the microwave is launched) to be strongly absorptive for the microwave in order to achieved the desired distributed modulation?

Reply: We thank the Reviewer for the excellent question. There is, in fact, no special engineering needed on the back (injecting) facet: the modulation standing wave forms when there are two counterpropagating microwaves in the cavity, for which only an injection at the back facet and a reflection at the front facet is required. We have actually performed extensive 3D RF/electromagnetic simulations of the entire laser device, bonding wires, and the RF PCB, including realistic material parameters with losses, to confirm the formation of (quasi) standing waves, and these results are reported already in Fig. 1(c) in the Main Text. These field distributions match very well with a 1D microwave transmission line model, which was subsequently used in the extensive numerical modeling of the modulated laser cavity

and the microwave and optical field dynamics. We should also note that while the coplanar waveguide RF PCB is designed for 50 Ω matching, the bonding wires and planarized waveguide are not matched to 50 Ω so there is some residual reflectance at this point, which reduces the RF coupling efficiency.

Reviewer Comment 1.5 — 5. It can be noticed that when the modulation frequency approached the 2nd harmonic, the gaps between adjacent pulses is filled with non-trivial residual field, as least within a certain range of position during each round trip (Fig.5c). Meanwhile the laser switched to double-pulse state given a modulation above the 2nd harmonic of the cavity resonant frequency (Fig.5d). I wonder if such switching to N=2 occurs as the intermittent field between the pulses arise as secondary pulses? It the repetition rate tuning still continuously during such switching of harmonic order ?

Reply: This is another excellent observation by the Reviewer. Our experiments and simulations indicate that the laser actually switches to a double pulse state already below $2 \times f_{\mathrm{rep},0}$, and the repetition rate tuning is still fully continuous for any frequency above the low-frequency modulation limit of 3.5 GHz, as verified in experiments (with a fine modulation frequency step) as well as in numerical simulations.

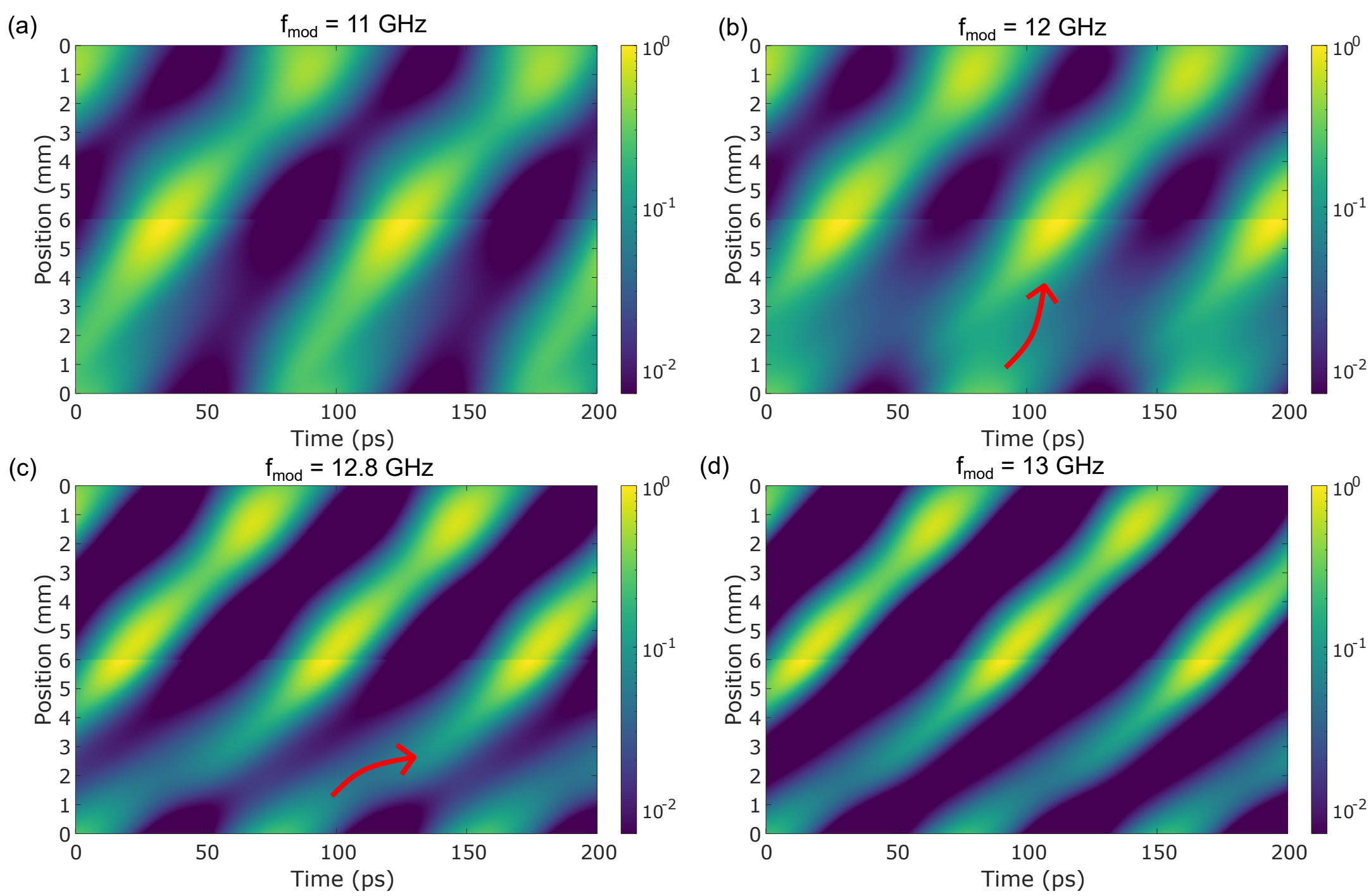

Figure 5: Numerical simulations of the intracavity fields for a varying modulation frequency, studying the transition between the single and double pulse regimes (red arrows), with the time dynamics displayed using the laboratory reference frame.

We performed additional numerical simulations to study this in greater detail. As shown in Fig. 5 (Fig. S19), we can observe the switching to a double pulse at a modulation frequency of 12.8 GHz, which is below the second harmonic. There is a significant intermittent field already at 12 GHz, but in this case, it is still part of the single pulse, as indicated by the red arrow in panel (b). At 12.8 GHz, these intermittent fields break off into separate pulses, generating a double pulse structure, as shown

in panel (c). It is important to note that, in terms of the intracavity field dynamics, these two cases are quite distinct. For example, for a modulation frequency of $f_{\mathrm{mod}} = 1.8 \times f_{\mathrm{rep},0}$, there is either a single pulse traveling with an increased group velocity of $v_{\mathrm{g}} = 1.8 \times v_{\mathrm{g},0}$, or two pulses traveling with a reduced group velocity of $v_{\mathrm{g}} = 0.9 \times v_{\mathrm{g},0}$. Whether the state will consist of a single fast pulse or a double slow pulse structure depends essentially on which state is energetically more favorable for the laser operation. Additionally, with an increasing modulation frequency, the microwave field distribution features an increasing number of maxima and minima along the cavity length, also contributing to the generation of several intracavity pulses (see also the simulated microwave field distribution as a function of frequency in Fig. 2(c) of the Main Text). Based on our numerical simulations, the transition to a double intracavity pulse regime happens already well below the second harmonic modulation frequency (at around $f_{\mathrm{mod}} = 1.85 \times f_{\mathrm{rep},0}$).

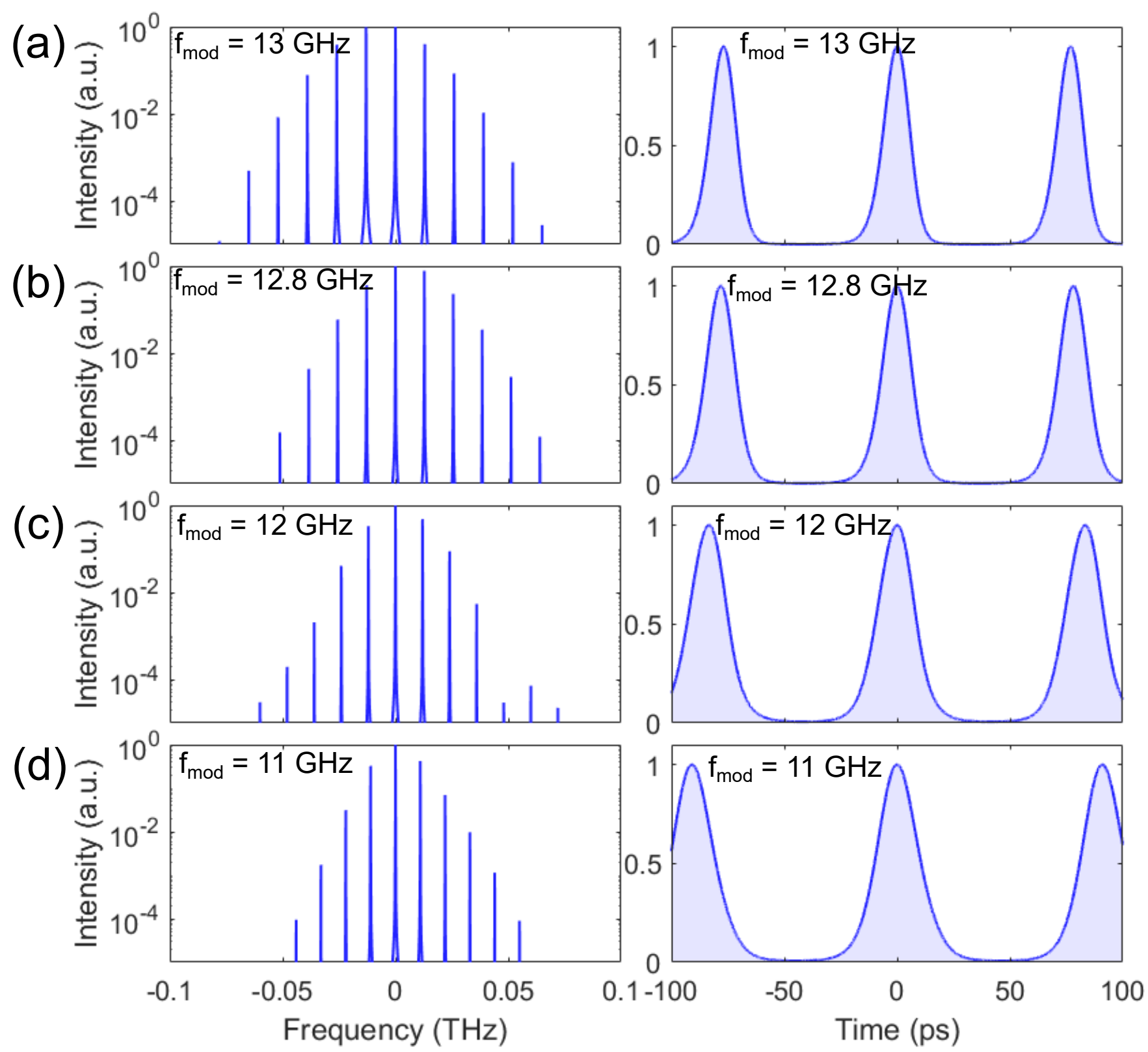

Figure 6: Numerically simulated output spectra and pulse trains display repetition rates exactly matched to the modulation frequency, without any clear spectral or temporal signatures differentiating between the single and double intracavity pulse regimes.

Nevertheless, in terms of the output intensity that is observable in experiments and important for applications, there is always a coherent pulse train with a repetition frequency that exactly matches the driving modulation frequency f_{mod}. Regardless of whether the laser operates in the single or double intracavity pulse regime, the output pulses are always well isolated and show no traces of intermittent field components. The output pulses can also be visualized in Fig. 5 as a line slice at the cavity position of 6 mm, indicating the location of the output facet. Even though the transition between the single

and double pulse regimes can occur abruptly as we increase the modulation frequency, the repetition rate tuning is still fully continuous and does not disrupt the mode-locked operation. This is also evident in Fig. 6 (Fig. S20), where there are no clear spectral or temporal signatures in the output emission spectra and pulse trains that would indicate a difference between the operation in the single and double intracavity pulse regimes.

This discussion and figure have also been added to the Supplementary Information, Section S10.2.

Reviewer Comment 1.6 — 6. The author claims that the tunable comb spacing can be used for high resolution gapless spectroscopy. However, the spectral envelop profile changed dramatically when the comb spacing is tuned (Fig.S2). In addition, the central comb position also seems to change under different modulation frequency. These features may make it difficult to use since the comb profile needs to be calibrated for each different modulation frequency. The authors are expected to address these concerns for this application.

Reply: The Reviewer raises an excellent point. In order to showcase the usability of our platform in practical applications, we have developed an absorption spectroscopy experiment using a tapered waveguide device, where both the spectral profile and the central emission frequency are relatively stable for a wide range of modulation frequencies while keeping all other crucial parameters fixed at constant values (DC laser bias, RF power, temperature, etc.). This way, we were able to measure a water vapor absorption peak at around 2.97 THz, showing excellent agreement with reference FTIR spectrometer measurements, and requiring only a simple THz intensity detector.

More details are provided in the Main Text and Methods, within sections entitled Absorption Spectroscopy.

Reviewer 2

The authors report on a novel modelocking regime in which coherent pulses can be generated with frequency spacings that are not defined by the physical cavity length in a monolithic geometry. This is possible because a sufficiently strong radio frequency modulation of the (semiconductor) gain medium detuned from the natural cavity resonance results in strong pulse shaping effects that change the group velocity of the pulse allowing to tune the pulse time spacing to an arbitrary modulation frequency. The manuscript presents experimental data using a THz quantum cascade laser as a test system for this idea and demonstrate continuous tuning of the pulse repetition rate from 4 to 16 GHz – both with lower and higher modulation than the natural cavity repetition rate of 6.6 GHz. They present both a simple analytical model based on the traditional Haus formalism for modelocking to explain the mechanism observed and numerical simulations for the specific experimental setup to confirm the observed behavior and parameters.

To the best of my knowledge, it is the first time such a continuous tunability of the repetition rate is demonstrated with a monolithic platform. The basic physics underlying the principles presented here have been treated theoretically long ago as well cited by the authors in particular in [17], but so far detuning the modulation frequency in active modelocking was rather seen as a source of instability and a curiosity, rather than an effect that can lead to useful cavity dynamics. In that regard, this manuscript presents an original, new point of view to an 'old' problem. The significance of the result is on rethinking active modelocking dynamics that could spark interest. In my opinion, this is the strongest point of interest of the manuscript. Concerning significance: modelocked lasers are tremendously important technologically for many fields and on-chip coherent pulse generation, in particular with tunability in a restrained geometry, are a big challenge. Having said this, I am not fully convinced of the immediate significance of this technology in its current state of performance. It may be possible, that some points could be better generalized and clarified in the manuscript to address these points:

Reviewer Comment 2.1 — • It seems that this new regime can only be stable in a rather niche technology case for semiconductor lasers with rather long pulses: from the analysis performed it seems these pulling time effects will only be effective for 'cleanly' shaping the group velocity if the pulses are long and dispersion and nonlinear effects can be neglected. I believe there is some analysis missing on whether this mechanism could be sustained in the case of more broadband, shorter pulse durations where effects such as dispersion, self-phase modulation, spectral filtering (and the resulting time clipping) etc, would be present and cannot be neglected. This would give the claims more generality for other laser types and would also broaden the application spectrum of such integrated lasers in general.

Reply: The Reviewer raises an important concern. With active mode-locking, the obtainable pulse durations are in the several picoseconds to tens of picoseconds range, as is demonstrated in this work. This is related to inherent pulse duration limitations of this technique due to gain-induced pulse broadening, as explained, for example, in the Kuizenga-Siegman model (D. Kuizenga & A. Siegman, "FM and AM mode locking of the homogeneous laser - Part I: Theory ", IEEE JQE 6, 11, 1970) and the Haus mode-locking theory (H. Haus, "A theory of forced mode locking", IEEE JQE 11, 7, 1975). Ultrashort (femtosecond) pulses are accessible with passive mode locking, e.g., using a saturable absorber (A.

Vladimirov & D. Turaev, "Model for passive mode locking in semiconductor lasers", PRA 72, 033808, 2005). However, in contrast to our work, with passive mode locking, there is no tunable parameter that would enable a significant tunability of the repetition rate of a monolithic device (without changing the physical cavity dimensions).

Compared to other semiconductor laser types, THz QCLs generally feature a significant amount of group velocity dispersion (GVD) due to the proximity of the emission frequency to the GaAs optical phonon Reststrahlen band (see also D. Burghoff, "Dispersion dynamics of quantum cascade lasers", Optica 3, 12, 2016). For example, the planarized THz QCL samples used in this work have a relatively large GVD value of around 5×10^4 fs^2/mm at 3 THz. Furthermore, QCLs exhibit strong resonant nonlinearities, often even referred to as "giant nonlinearity" (see, for example, Opacak, et al., "Frequency comb generation by Bloch gain induced giant Kerr nonlinearity", PRL 127, 093902, 2021), and also noticeable gain filtering (gain bandwidth ∼1 THz). The THz QCL active region material used in this work has a relatively large Kerr nonlinearity with a nonlinear refractive index of $n_2 = 1.8 \times 10^{-14}$ m^2/W. This is significantly larger compared to some other commonly used materials, e.g., bulk GaAs: $n_2 = 1 \times 10^{-17}$ m^2/W (Hurlbut, et al, "Multiphoton absorption and nonlinear refraction of GaAs in the mid-infrared", Optics Letters 32, 6, 2007); Si_3N_4: $n_2 = 4.8 \times 10^{-19}$ m^2/W (Ikeda, et al., "Thermal and Kerr nonlinear properties of plasma-deposited silicon nitride/silicon dioxide waveguides", Optics express 16, 17, 2008); $LiNbO_3$: $n_2 = 1.8 \times 10^{-19}$ m^2/W (Zhu, et al. "Integrated photonics on tin-film lithium niobate", Adv. in Opt. and Phot. 13, 2, 2021).

While the simplified analytical model, where some effects are neglected, is used to pinpoint the basic physical mechanism (pulse pulling), all of these effects are fully considered in our extensive numerical simulations. Thus, with respect to these parameters, THz QCLs are not in a specifically favorable regime among semiconductor lasers. Still, the resulting pulses are comparably short in perspective to the THz carrier frequency, i.e., a pulse encompasses roughly 17 optical cycles for resonant modulation. According to Fig. S2(a), the comb spectrum then spans approximately 20% of the center frequency. Concerning laser types beyond semiconductor lasers, applications based on solid state and fiber lasers are not an on-chip platform as targeted in this paper. In addition, the realization of distributed modulation along the whole geometry (rather than modulation in a lumped absorber element, as common in mode-locked solid state and fiber lasers) is crucial for this approach, as emphasized in the paper.

These properties and conclusions were now added to the Methods section and to the Supplementary Information, section S1.

Reviewer Comment 2.2 — • The main application put forward is that of frequency comb spectroscopy. Whereas this seems appropriately identified since tunable repetition rate would allow to resolve features 'missed' or poorly resolved in static repetition rate spectroscopic experiments (in particular at very high repetition rates), it would seem that the point above of the rather long pulse duration and rather narrowband operation is a strong limitation for this particular application. Can this concept be combined with central frequency tunability on chip?

Reply: The Reviewer raises an important concern about the limited bandwidth and pulse durations. As explained in the previous answer, these are inherently limited by the active mode-locking mechanism itself and are comparable to existing active mode-locked semiconductor lasers in the literature. As discussed in the Main Text and Methods of the revised Manuscript, we have now successfully demonstrated an absorption spectroscopy experiment using a tapered tunable comb laser (see also our answer to the next question below).

In terms of tuning the central emission frequency, there are several possible tuning knobs: the operating temperature, the DC laser bias, and the RF power can all contribute to shifting the emission wavelength. This is already visible in Fig. S12 for the homogeneous ridge sample, where different operating conditions, namely DC bias and RF power, result in a change of the central emission frequency. We are now also adding a new figure of the experimental emission spectra of a resonantly modulated tapered device ($f_{\mathrm{mod}} \approx f_{\mathrm{rep},0}$), where the central frequency is blue-shifted during an increasing DC laser bias sweep, shown also below in Fig. 7 (Fig. S14). We should note that in this example, we are sweeping across the entire DC bias voltage range of a resonantly modulated device, so the emission bandwidths and central frequency shifts are relatively large, but this effect can also be employed for detuned modulations within smaller ranges of DC bias voltages (closer to the lasing threshold), where the tunable mode-locking is stable. In principle, one could also think of seeding the central emission frequency with another single-mode laser that is coupled into the tunable comb laser cavity. Additionally, if aiming for a more widely separated frequency range, THz QCLs allow for a very flexible engineering of the emission frequency by modifying the active region design, i.e., by changing the unit cell layer thicknesses of the GaAs/AlGaAs superlattice, such that specific frequency regions can be targeted.

This discussion and the new figure were also added to the Supplementary Information, section S8.

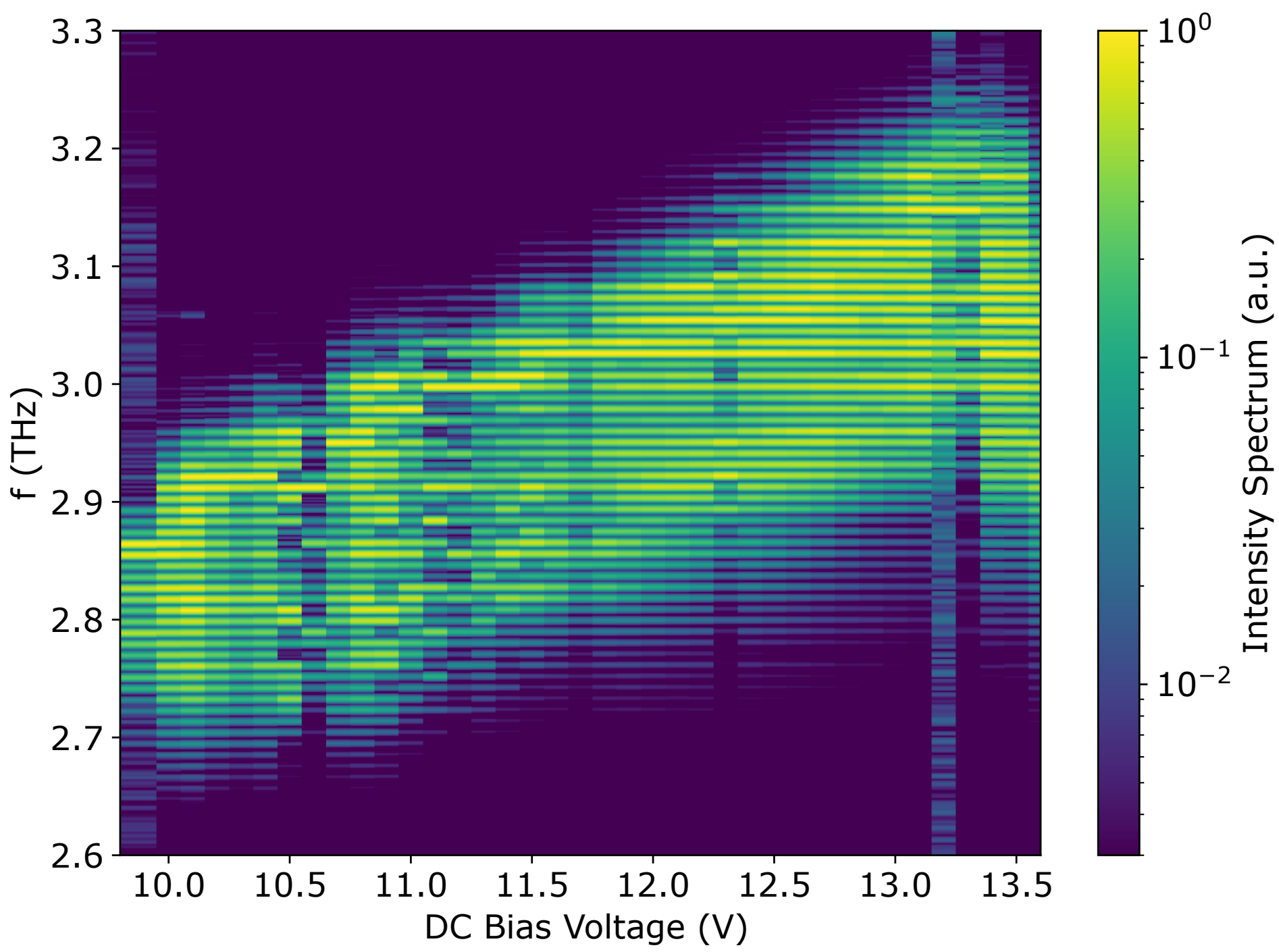

Figure 7: Experimental DC bias voltage sweep of a tapered device at resonant modulation conditions ($f_{\mathrm{mod}} \approx f_{\mathrm{rep},0}$), highlighting the blue-shifted central emission frequency with increasing bias.

Reviewer Comment 2.3 — • In the Supplementary Information, Fig. S15 indicates that the central wavelength but also the overall bandwidth significantly changes while the repetition rate is tuned. The tapered device Fig. S 16 performs better and possibly could be more practically

used for spectroscopy, however it is not clear why it is not possible to perform a proof of principle experiment of absorption spectroscopy using this comb system. It would make the case of the usefulness of the devices much stronger.

Reply: We thank the Reviewer for this excellent comment, which encouraged us to develop a proof-of-principle absorption spectroscopy experiment and demonstrate the practical applicability of our tunable comb system. In particular, we used a tapered device, where the central emission frequency is stable as a function of RF modulation frequency. In fact, during the spectroscopy experiments, all the other crucial parameters, i.e., temperature, DC laser bias, and RF power, were kept constant. The only variable parameter was the RF modulation frequency itself, making the system very straightforward for general users. After the tunable comb laser output is directed to a sample (with absorption features), it is coupled to a THz intensity detector, which is basically the only additional component required in the experimental setup, without the need for any interferometric or filtering components. The frequency-dependent transmission is then reconstructed from the matrix product of the measured emission and transmission spectra with an algorithm. In this way, we were able to successfully measure water vapor absorption at 2.97 THz and also verify it with a reference FTIR spectrometer measurement, showing good agreement.

More details are provided in the Main Text and Methods, within sections entitled Absorption Spectroscopy.

Reviewer Comment 2.4 — • Generally, parameters vary significantly when tuning the repetition rate, which makes the tunability aspect somehow questionable in general. It would be useful for the reader to present a more detailed path forward to have tunability without significant changes in other output parameters.

Reply: The Reviewer raises a valid concern. It is true that the parameters (DC laser bias and RF power) and the emission spectra can vary significantly for the homogeneous ridge device, but once the parameters are identified, they can be programmed into the sweep (the parameter settings and the corresponding spectra are reproducible).

More importantly, for both of the tapered devices presented, the data were obtained with a simple sweep of the RF modulation frequency, while all the other crucial parameters (temperature, DC laser bias, and RF power) were stabilized and kept at constant values. Moreover, throughout the modulation frequency sweep, the central emission frequency remains stable, making these devices usable for practical applications, as we have now also demonstrated with absorption spectroscopy experiments (see also the previous answer and more details in the Main Text and Methods (sections entitled Absorption Spectroscopy) and Supplementary Information, section S8).

Reviewer Comment 2.5 — • As pointed several times by the authors, effective spatio-temporal gain modulation is only effective when operating the laser close to threshold, but threshold operation is often associated with noisy operation regimes. It would be important to comment on this point in particular on the linewidth of the obtained combs, when comparing to other comb technologies. In the Supplementary Information, there seems to be some variability in the linewidth as well in the different operation points.

Reply: The Reviewer has another excellent comment. The fact that mode-locked operation is here relatively close to threshold is not unique to the tunable setup, but is common for mode-locked QCLs (see, e.g., C. Y. Wang et al., "Mode-locked pulses from mid-infrared quantum cascade lasers," Optics

Express 17, 12929, 2009; J. Faist et al., "Quantum cascade laser frequency combs," Nanophotonics 5, 272, 2016). It is true that, compared to operation at a higher DC laser bias, operating the laser close to threshold tends to be noisier due to a relatively larger spontaneous emission rate. However, compared to other semiconductor lasers, QCLs feature very low spontaneous emission rates, small linewidth enhancement factors, and very narrow linewidths (see, for example, T. L. Myers, et al., "Free-running frequency stability of mid-infrared quantum cascade lasers", Opt. Lett. 27, 3, 2002). Additionally, it has been shown that QCLs and QCL-based frequency combs feature very narrow intrinsic linewidths down to (sub)kHz levels (see Capelli, et al., "Intrinsic linewidth of quantum cascade laser frequency combs", Optica 2, 10, 2015, and Consolino, et al., "Fully phase-stabilized quantum cascade laser frequency comb", Nature Comm. 10, 2938, 2019).

Therefore, despite being operated close to the laser threshold, these states are in fact fully coherent, as proven experimentally via SWIFTS, which is very sensitive to coherence (see also Z. Han, et al., "Sensitivity of SWIFT spectroscopy", Optics Express 28, 5, 2020), and with extensive numerical simulations.

If in the last sentence, the Reviewer is referring to the experimental spectra in Fig. S3, the emission lines only appear to be broader for lower modulation frequencies due to a limited resolution of the FTIR spectrometer and not due to increased linewidths (as explained in the corresponding text in detail, this is related to the limited mirror delay length and the Fourier Transform). We have also shown in simulations that the combs are fully coherent, with narrow linewidths that satisfy the community-accepted conditions for mode-locked pulses (noise quantifiers), as we explain in the Main Text section Coherent measurements and simulations, and in the Supplementary Information sections S2 and S3, which have now also been expanded accordingly.

Reviewer Comment 2.6 — • This technique allows to tune the repetition rate, but it is unclear how is the carrier envelope offset frequency stability affected by operating in this regime. This deserves a more detailed discussion as well.

Reply: The Reviewer raises an important point. We should first emphasize that electrically pumped semiconductor lasers, including QCLs, are generally not CEO-stabilized, as most CEO detection techniques require an octave-spanning coherent comb spectrum for f-2f self-referencing. However, once the repetition rate is stabilized (e.g., via active mode-locking), the f_{ceo} can be tuned by an actuator, which in the case of QCLs can be achieved via the injection current (DC laser bias) or the operating temperature, which both modify the refractive index of the active material. The f_{ceo} stability is also directly related to the coherence and linewidth of the laser, and as discussed already in the previous answer, the tunable comb states are fully coherent, with simulations suggesting that there is no significant effect on CEO stability with modulation frequency detuning beyond the longer cavity build-up/stabilization times (see also the Supplementary Information, updated sections S8 and S10.1).

Reviewer Comment 2.7 — With all the parameter limitations as correctly identified by the authors that limit also the tunability range in practice, I ask myself if this technique and the technology has really the potential to surpass more complex, but more standard, techniques to tune the repetition rate in integrated geometries – perhaps not fully continuously. For example Mach Zender interferometers have been shown in integrated platforms to provide a lot of parameter flexibility for repetition rate multiplication. It would also be good to refer to the special advantages of this technique comparing with external techniques as well.

I would also like to refer the authors to the case of random fiber lasers, where I believe such spatio temporal gain dynamics are used for similar cases of 'mode-less' cavities. I believe it makes sense to draw some parallels to this technology as well. For example: von der Weid, J.P., Correia, M.M., Tovar, P. et al. A mode-locked random laser generating transform-limited optical pulses. Nat Commun 15, 177 (2024) for one recent publication in this area.

Reply: We thank the Reviewer for bringing our attention to these relevant examples.

In the case of MZIs, these are indeed useful for repetition rate multiplication, but require additional components and only allow for discrete changes in the repetition rate. This is essentially the same as harmonic mode locking, which inherently results in spectral gaps (in terms of going to higher harmonics, the spectral resolution is reduced drastically, by a factor of N for the Nth harmonic order).

There are also external cavity lasers, where a movable mirror is used to change the cavity dimensions and tune the repetition rate. This allows for a continuous tuning of the repetition rate, but requires a more complex setup with external components, slower tuning times, stringent free-space alignment of the entire external cavity, and also brings additional sources of noise, such as mechanical vibrations.

In the case of the random fiber laser reference, this involves the electrical time gating of a lumped gain element (SOA) in a long fiber cavity with a random distributed mirror. While they do demonstrate an electrically tunable repetition rate between 0.714 - 1.120 MHz (less than a factor of two, which is not sufficient to cover all the spectral gaps), it is essentially the cavity length that is adjusted such that the roundtrip time of the optical pulse is synchronized to the electrical gating of the lumped gain/modulator element. In other words, there is no gain/modulator effect on the pulse propagation (no pulse pulling effect with tunable group velocities), but rather an effective cavity length change in the form of a random distributed mirror, and in this way more comparable to cavities with tunable mirrors, where the cavity length and modulator frequency are both adjusted. Moreover, the produced pulse durations are very long (0.3-20 ns) with very modest optical bandwidths below 250 MHz, and the system consists of a number of separate components (SOA, pump laser, Er-doped fiber, single-mode fiber, random distributed reflector), so it is not really comparable with our system.

In summary, the advantage of our system compared to all of these examples is that it simultaneously combines the continuously and widely tunable repetition rate without any spectral gaps while using a simple monolithic semiconductor laser cavity, without any additional components or external parts of the setup, constituting a robust and reliable system with a compact on-chip footprint and straightforward operation by tuning only one parameter - the RF modulation frequency (especially in the case of tapered devices).

We have expanded the comparison to other systems in the Conclusion section of the Main Text.

Reviewer Comment 2.8 — The data and methodology used in the paper are of excellent quality as well as the presentation of the results. The conclusions are valid and relevant work is appropriately cited to the best of my knowledge – with some suggestions above to expand the scope.

Concerning the overall clarity of the manuscript it would be useful for a general audience to have a more general introduction presenting also other comb technologies and their capabilities (not only in terms of repetition rate tunability, but other parameters of relevance) to be able to place the results in proper context, and how and in which cases this new method promises to outperform other integrated technologies. This may also be appropriate in the conclusion section, as the experimental results have already been presented.

Reply: We thank the Reviewer for the kind words and the excellent suggestion. We have expanded the Conclusion section in the Main Text to provide a broader context of our results as recommended.

Reviewer 3

The manuscript "Continuously tunable coherent pulse generation in semiconductor lasers" by Urban Senica et. al reported a monolithic semiconductor laser with a continuously tunable repetition rate from 4∼16 GHz. The authors proposed a novel regime, where the repetition rate of a mode-locked semiconductor laser can be tuned continuously and significantly both above and below the fundamental frequency defined by the cavity length. The presented results are encouraging and novel. But I do have some major concerns in terms of the practical applications of their work. On one hand, the modulation amplitude needs to be large enough for the spatiotemporal gain modulation to generate the tunable coherent pulse, for example, the RF powers are all above 30 dBm in the manuscript. This may severely limit their applications in the claimed fields such as sensing or telecommunications. On the other hand, the authors describe in the manuscript and in Supplementary Information that the measurements are operated inside a shield under low vacuum conditions of 10-6 mbar and cooled temperatures ∼40 K, I wonder why such extreme operating conditions are needed? As far as I know, the THz QCLs can be operated in >150 K. If such extreme conditions are necessary, it may also severely limit their usage. It would be beneficial to provide some insights on what conditions are needed for applying this concept to widely used semiconductor lasers such as DFBs, FPs, etc.

Some minor comments are given below:

Reply: We thank the Reviewer for the insightful comments. Regarding the required RF powers, these can be reduced with optimized RF coupling and waveguide geometries and using a low-power active region/gain material (less RF power required to induce sufficient current modulation and voltage swing across the device). Currently, the RF coupling efficiency bottleneck is due to the bonding wires between the coplanar RF PCB and the planarized waveguide. A possible further improvement would be using a touch down RF probe (G-S-G) and optimizing the design of the waveguide extended top metalization to be matched to 50 Ω (we discussed the possibility of microwave and impedance design of planarized waveguides in more detail in Ref. [7], Senica, et al., "Planarized THz quantum cascade lasers for broadband coherent photonics", Light: Science & Applications 11, 347, 2022).

It is true that the homogeneous ridge device is operated in a low vacuum environment at a heat sink temperature of 40 K, but this is mainly to obtain the best possible performance for these samples (minimize atmospheric absorption and maximize output power), and it is not an inherent limitation. For example, one of the tapered waveguide samples was operated at 80 K, compatible with liquid nitrogen operation, and we had previously demonstrated frequency comb operation up to 115 K in CW with a tapered device in Ref. [23] Senica, et al., "Frequency-Modulated Combs via Field-Enhancing Tapered Waveguides", Laser & Phot. Rev. 17, 12, 2023. High-temperature THz QCLs are indeed getting close to room temperature (261 K = -12 °C), but those devices are mostly optimized for narrowband operation in pulsed mode (see also Khalatpour, et al., "Enhanced operating temperature in terahertz quantum cascade lasers based on direct phonon depopulation", Applied Physics Letters 122, 16, 2023). However, broadband active regions are also expected to raise the operating temperatures, and this concept is applicable also to other semiconductor lasers that work at room temperature. Therefore, we expect such tunable mode-locked devices to operate also at ambient conditions, which would make them widely applicable also outside the lab.

In terms of the types of semiconductor lasers that this approach is applicable to, there are two

fundamental conditions that need to be fulfilled. First of all, the laser cavity needs to support broadband operation, such that many longitudinal modes can be excited simultaneously. Secondly, the cavity needs to support the formation of a (quasi) standing RF wave along the entire device, such that a traveling gain window with a velocity proportional to the modulation frequency is generated. Both of these conditions can be fulfilled by Fabry-Pérot semiconductor lasers. Frequency-selective cavities, such as narrowband DFB and DBR semiconductor lasers, are not appropriate, since they severely limit the emission bandwidth, often targeting single-mode operation.

We have expanded the Conclusion section of the Main Text and the Methods section.

Reviewer Comment 3.1 — 1. The authors provide limited info about the sample, for example, 6 mm long, 40 µm wide homogeneous ridge. More details would be helpful, such as specific materials and dimensions of waveguide, metals, III-V substrate, the waveguide loss, the reflection coefficient of waveguide facets, heat dissipation, microwave properties, etc.

Reply: We thank the Reviewer for the suggestion, and extensive additional details about the samples have now been added to the Methods section. A complete study of planarized THz QCLs using the same active region material, including further details on the fabrication process, the optical, RF, and thermal properties, and the simulation and measurement results, can be found in Ref. [7], Senica, et al., "Planarized THz quantum cascade lasers for broadband coherent photonics", Light: Science & Applications 11, 347, 2022.

Reviewer Comment 3.2 — 2. What is the photoelectric conversion efficiency of the sample? Is there any difference between the photoelectric conversion efficiencies of resonant frequency, harmonics and other frequencies?

Reply: The Reviewer raises an excellent question. Due to relatively high threshold current densities (100 - 1000 A/cm^2) and relatively high operating voltages (typically above 8 V) required to align the cascade structures, THz QCLs inherently have a significant electrical power consumption (see also Razeghi, et al., "High-power, high-wall-plug-efficiency quantum cascade lasers with high-brightness in continuous wave operation at 3–300 μm", Light Sci & Appl 14, 252 (2025)). In combination with limited thermal management compared to buried and/or epitaxial-side-down mounted semiconductor lasers, this results in modest wall plug efficiencies, i.e., a conversion between the electrical input power to optical output power of, at most, only a few percent ($\lesssim 2\%$).

In the case of our homogeneous ridge sample presented in the Main Text, at the maximum DC bias point, we are applying a voltage of 10 V and a current of 0.7 A, i.e., an electrical consumption of 7 W. The measured output in pulsed mode is around 2.5 mW. Doubling this value to include the back facet output coupling and dividing by the electrical power consumption, we get an external wall plug efficiency of around 0.1%. Here, we should emphasize that the mirror losses $a_{\mathrm{mirr}} = -\frac{1}{2L}\ln(R_1 R_2)$ are relatively low due to highly reflective cleaved facets ($R_1 = R_2 = 70\%$) and the relatively long waveguide length ($L = 6$ mm). Mirror losses are directly related to output coupling and slope efficiency via the expression $\frac{\mathrm{d}P}{\mathrm{d}I} = \frac{N_{\mathrm{p}} h\nu}{e}\frac{a_{\mathrm{mirr}}}{a_{\mathrm{tot}}}\frac{\tau_{\mathrm{eff}}}{\tau_{\mathrm{eff}}+\tau_2}$, where a_{mirr} and a_{tot} are the mirror and total loss, τ_{eff} and τ_2 are the carrier lifetimes, N_{p} is the number of periods, ν is the laser frequency, h is Planck's constant and e is the elementary charge. For THz QCLs, the very low emission frequencies ν of around 3 THz also contribute to a relatively low slope efficiency. However, this can be improved significantly with cavity engineering, e.g., using low-reflectivity end facets and surface-emitting antennas (see also Senica, et al., "Broadband surface-emitting THz laser frequency combs with inverse-designed integrated reflectors",

APL Photonics 8, 096101 (2023), where we demonstrated a factor of 7 improvement in slope efficiency and output power). Moreover, the active material we use in this work is optimized for broadband operation, not for high power and high wall plug efficiency (even when optimized for those figures of merit, THz QCLs only reach around 2% wall plug efficiency, see also Li et al., "High power-efficiency terahertz quantum cascade laser", Chinese Physics B, 25, 8 (2016)).

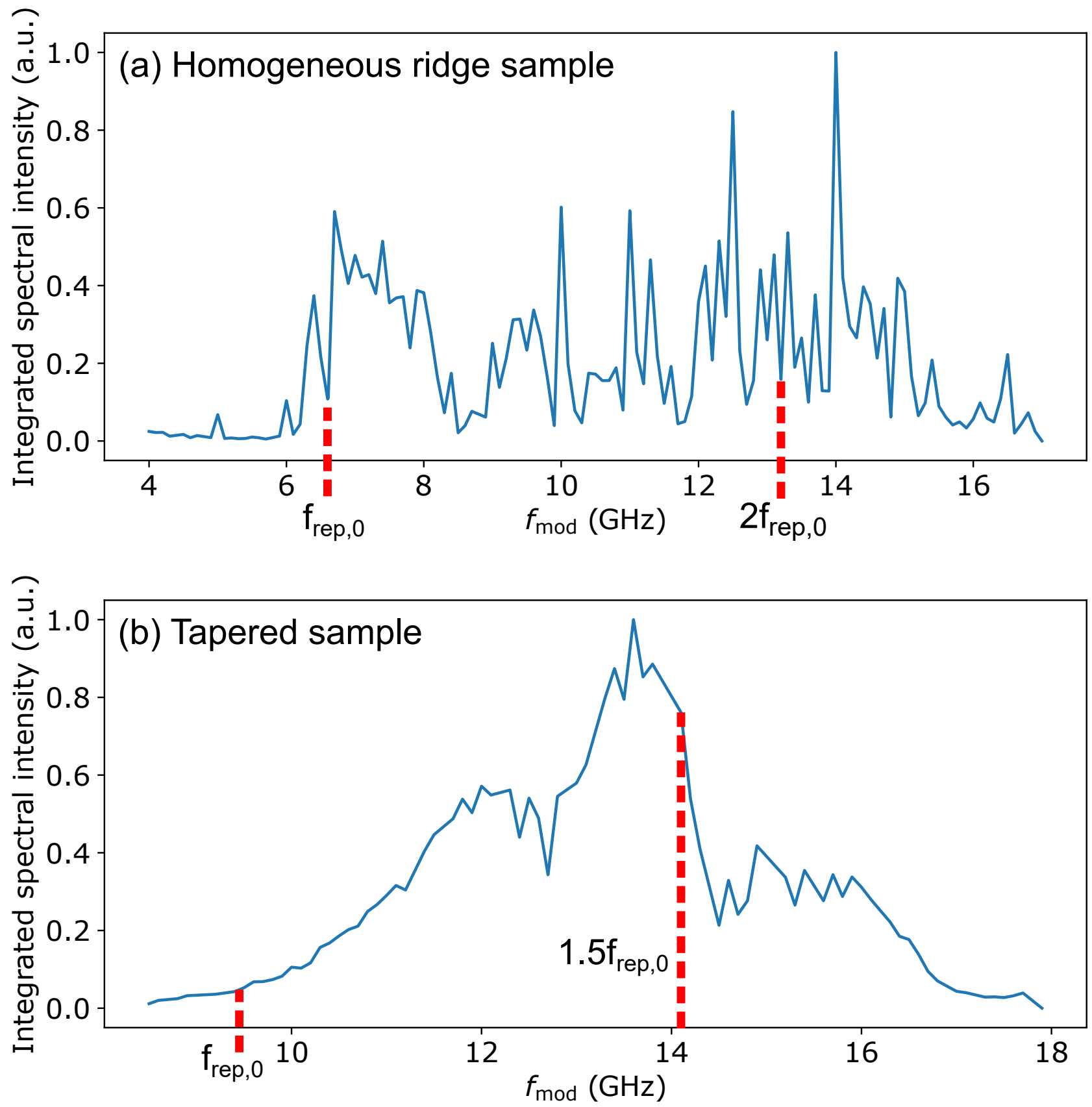

Figure 8: Integrated spectral intensity of an experimental sweep of the modulation frequency of **(a)** the homogeneous ridge sample and **(b)** the tapered sample. The latter shows a clear trend of increasing the output power when the modulation frequency lies between the fundamental and second harmonic cavity resonant frequency.

The Reviewer also raised an interesting comment about the efficiency dependence on the modulation frequency. Intuitively, we expect the efficiency to be largest at resonant modulation conditions ($f_{\text{mod}} = N \times f_{\text{rep},0}$), when the optical pulse is co-propagating with the traveling net gain window without any phase delay. In order to gain an experimental insight, we plot the integrated spectral intensity as a function of frequency detuning, normalized to the largest value over the entire sweep. In the case of the homogeneous ridge sample in Fig. 8(a) (Fig. S16), the intensity is oscillating without a clear trend, which is due to simultaneously changing the DC laser bias and RF power along with the RF modulation frequency. However, plotting the dependence for the tapered waveguide sample in Fig. 8(b), where only the RF modulation frequency is changing with all the other parameters kept constant, there is in fact a clear trend. The integrated spectral intensity (proportional to the output power and wall plug efficiency)

is actually increasing with the modulation frequency detuning, reaching a maximum between the resonant harmonics. The explanation is as follows: when the modulation frequency detuning is increased, the phase delay between the traveling gain window and the optical pulse increases. Additionally, the optical pulses have longer durations, i.e., they are more stretched out throughout the cavity and have lower peak intensities. In our THz QCL device with relatively short gain recovery times, these conditions result in a reduced gain saturation, effectively increasing the gain per roundtrip and generating higher output powers. In the resonant modulation case, the emission spectra are the broadest and the optical pulses are the shortest, however, the shorter optical pulses with higher peak intensities also induce a larger gain saturation. Therefore, the larger the modulation frequency detuning away from the resonant harmonics, the greater the output powers (and wall plug efficiencies), resulting in a maximum value in the proximity of $f_{\mathrm{mod}} = 1.5 \times f_{\mathrm{rep},0}$.

This discussion and the new figure were also added to the Supplementary Information, section S9.

Reviewer Comment 3.3 — 3. The authors mentioned that the high modulation frequencies are in principle high, provided that the modulation amplitudes are large, the microwave losses are low, and the modulation frequency is lower than the laser gain bandwidth. How large is the amplitude? How small is microwave loss? And how broad is the gain bandwidth? Theoretically, is there an exact modulation frequency limit?

Reply: We thank the Reviewer for the relevant comment. With our statement, we wanted to emphasize the general conditions required for achieving the tunable mode-locked regime also for relatively high modulation frequencies, i.e., for inducing a sufficient spatiotemporal gain modulation along the entire laser cavity. With increasing frequency, the microwave losses of the cables, wire bonds, as well as the propagation losses within the planarized waveguide sample itself, can increase significantly. These can occur due to conductor losses (skin effect), substrate losses (loss tangent), and radiative losses, which all increase with frequency. For sufficiently large losses, the microwaves injected at the back facet would be strongly attenuated before reaching the front facet, hindering the formation of a standing RF wave and the traveling gain window.

For studying the microwave losses of our system, we performed frequency-dependent eigenmode numerical simulations of the planarized waveguide 2D cross-section, using realistic material properties with losses. As shown in Fig. 9 (Fig. S8), at a frequency of 10 GHz, the microwave effective index is around 2, while the propagation losses are around 1.2 dB/mm, which increase exponentially with frequency (dB/mm units). These properties are also included in our extensive numerical simulations of the modulated laser cavity in the form of a 1D microwave transmission line model.

Experimentally, in our specific system and sample, the required RF modulation powers (applied at the source) are generally at +25 dBm or above. In terms of studying the effects of microwave loss, we did not perform an experimental study as these are fixed by the sample geometry, and the highest modulation frequency of 19 GHz was limited by our RF source and amplifier equipment. Therefore, we could not identify the highest modulation frequency experimentally, but we did show in simulations that modulation frequencies as high as $f_{\mathrm{mod}} = 35$ GHz $> 5 f_{\mathrm{rep},0}$ would still work, as can be seen in Fig. 10 (Fig. S9). Such high modulation frequencies have already been demonstrated for resonantly modulated THz QCLs (see Gellie, et al., "Injection-locking of terahertz quantum cascade lasers up to 35GHz using RF amplitude modulation", Optics Express 18, 20, 2010, and also Maineult, et al., "Microwave modulation of terahertz quantum cascade lasers: a transmission-line approach", Appl. Phys. Lett. 96, 021108, 2010). Additionally, we performed a numerical simulation study, where we show that in the case of significantly increased microwave propagation loss (4.3 dB/mm), the output pulse stream experiences

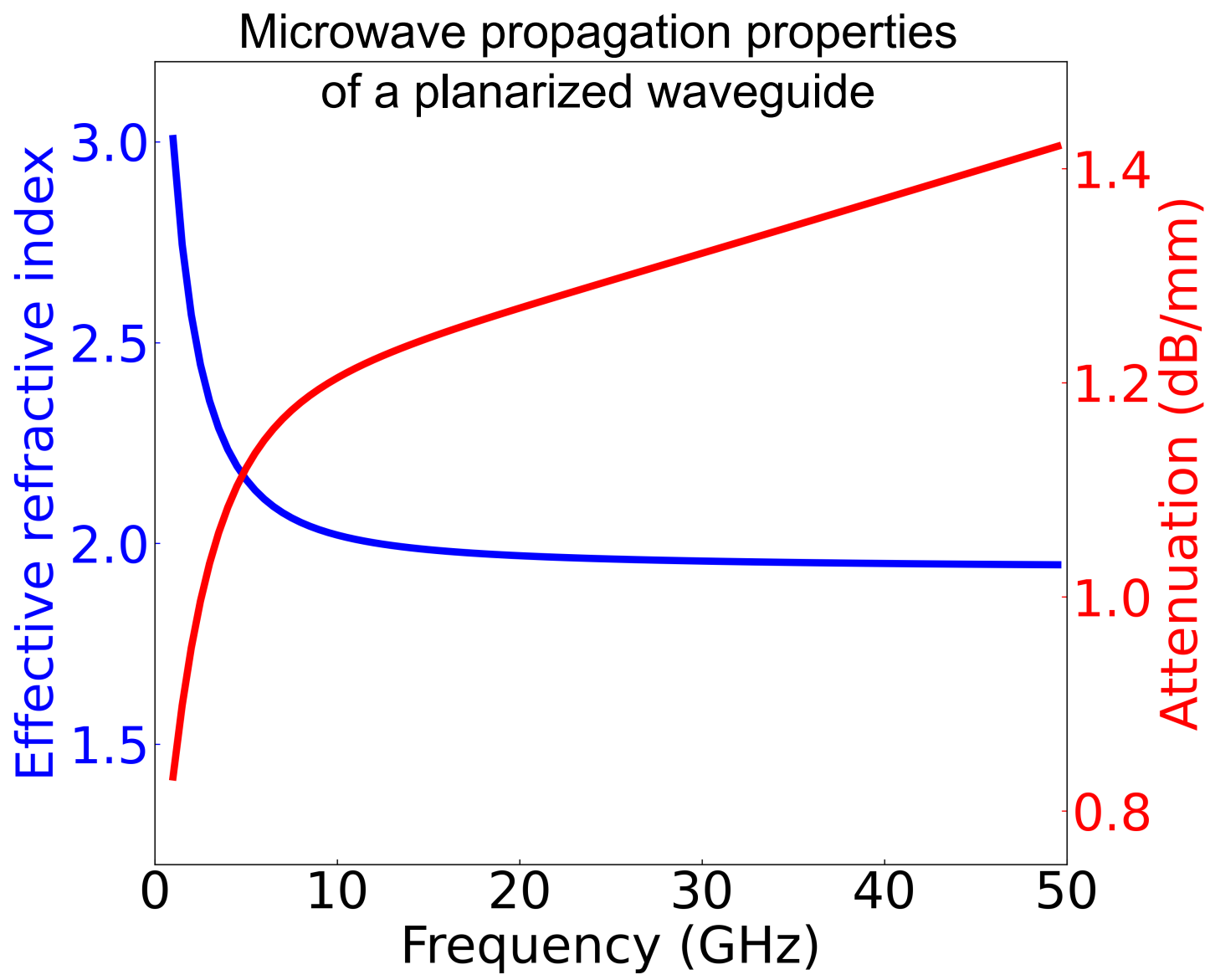

Figure 9: Simulated microwave propagation properties of a planarized waveguide with an active waveguide width of 40 µm, height of 10 µm, and top metal width of 300 µm and thickness of 500 nm.

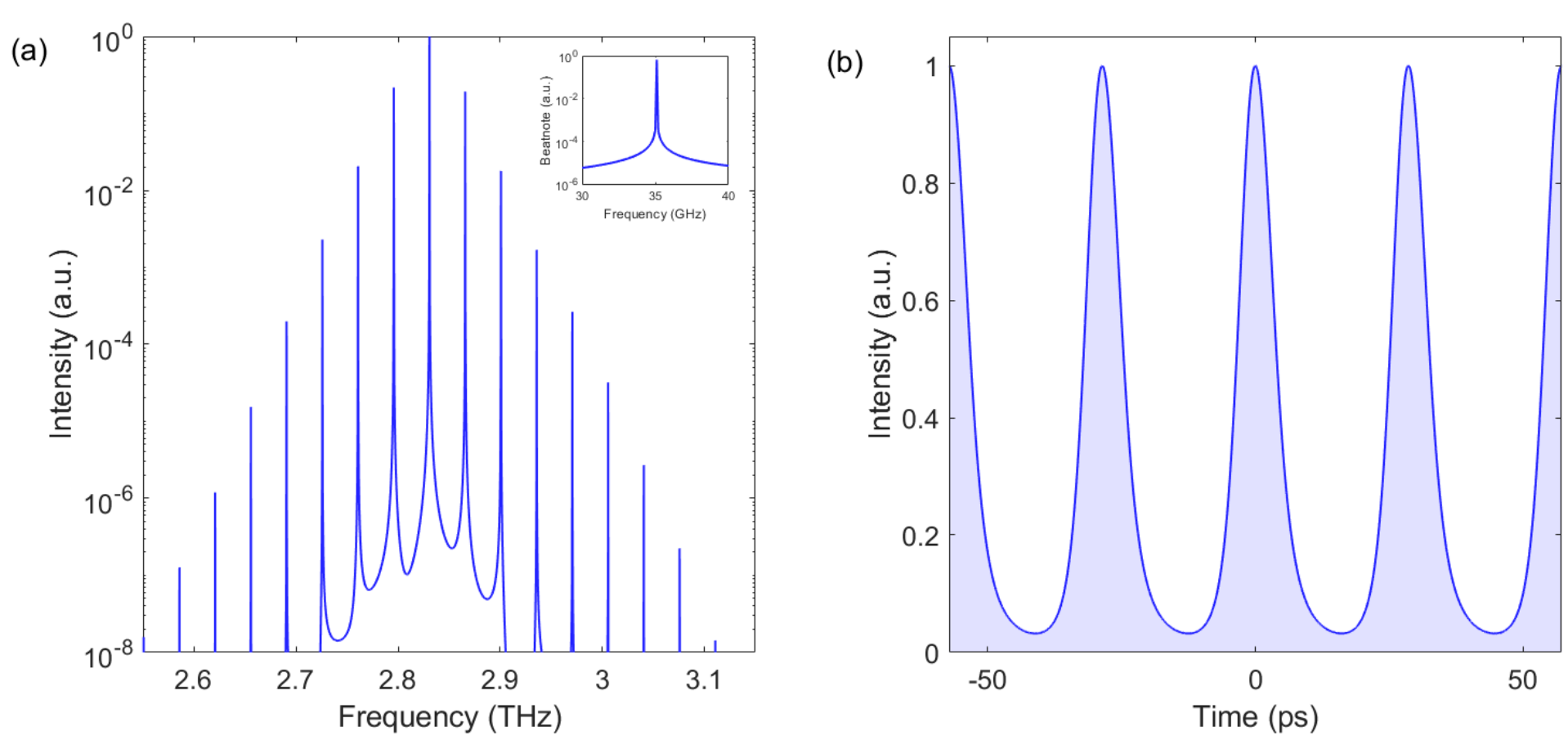

Figure 10: Simulation result with a modulation frequency of $f_{\text{mod}} = 35$ GHz $> 5 f_{\text{rep},0}$. This simulation shows that the tunability is, in principle, possible for much higher modulation frequencies than the current experimental limit. **(a)** displays the intensity spectrum and **(b)** the corresponding time domain data.

intensity fluctuations and does not support a coherent mode-locked state, resulting instead in a noisy emission spectrum, as shown in Fig. 11 (Fig. S23).

In terms of the theoretical high-frequency modulation limit, neglecting the limitations of RF equipment and microwave loss that we discussed in the previous paragraphs, there are two additional limiting factors: the gain bandwidth and the gain recovery time. High-frequency modulation is also closely

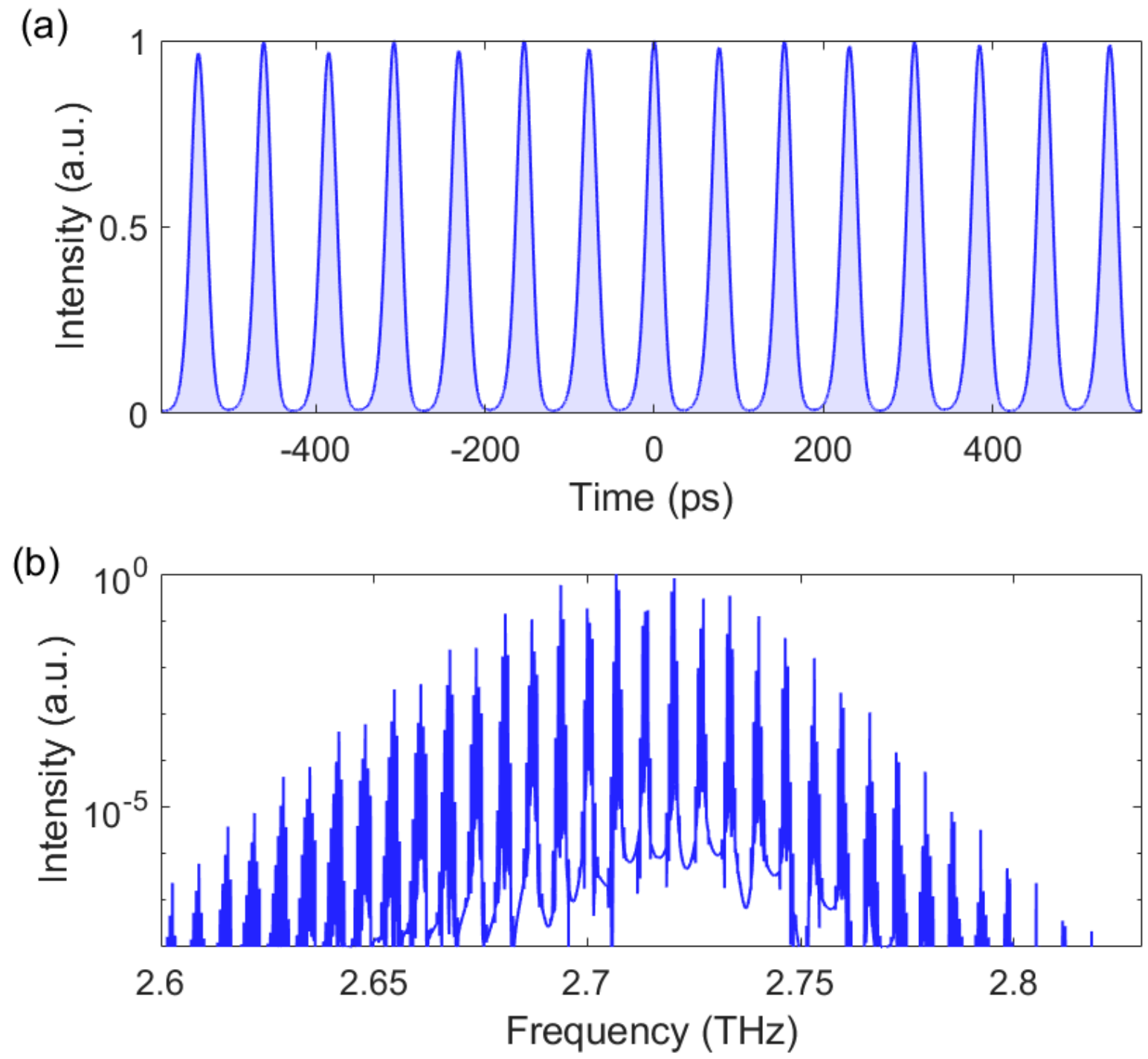

Figure 11: Simulation results for increased microwave losses of 4.3 dB/mm. **(a)** Out-coupled intensity as a function of time and **(b)** corresponding intensity spectrum.

related to harmonic mode locking, since several pulses will be propagating within the cavity simultaneously. With increased repetition rates, the pulses are shorter and closely spaced in time. In this context, it was found that short gain recovery times support the propagation of harmonic pulses (see also Silvestri, et al., "Harmonic active mode locking in terahertz quantum cascade lasers", Phys. Rev. A 108, 013501, 2023). Additionally, even with extremely fast gain dynamics, the final limitation is the gain bandwidth: shorter pulses feature larger mode spacings and broader optical bandwidths; however, only emission frequencies that lie within the laser gain bandwidth can be generated, since the active material needs to provide sufficient gain to overcome the roundtrip losses to sustain lasing action. Therefore, the gain bandwidth needs to be sufficiently wide, such that several modes can fit within this bandwidth for the given modulation frequency. In our case, the active region we used has a relatively broad gain bandwidth of around 1 THz, and a relatively short gain recovery time of around 10 ps, so theoretically, modulation frequencies in the order of around 100 GHz could be possible.

This discussion and figures were also added to the Supplementary Information, sections S6 and S10.5.

Reviewer Comment 3.4 — 4. The spatiotemporal gain modulation is implemented by injecting microwaves into waveguide to generate a standing wave, does this mean that the generated pulse train is still constrained by the FSR and the tuning is not strictly continuous?

Reply: We thank the Reviewer for the excellent question. The microwaves are injected into the waveguide from the back facet, propagate along the entire waveguide length, and are reflected at the front facet. The interference between the two counterpropagating waves generates a (quasi) standing

wave. The latter does depend on the frequency, however, we are not actually exciting a resonant microwave mode of the cavity. Instead, an oscillating microwave field distribution is formed along the waveguide for any RF frequency, due to the interference between the forward and backward propagating microwaves (see also the simulated field distributions as a function of modulation frequency in Fig. 1(c)). This, in turn, generates a traveling net gain window, which induces the pulse pulling effect that drives the tunable mode-locked pulse mechanism. The velocity of the traveling net gain window (or, equivalently, the phase velocity of the microwave field) is proportional to the modulation frequency, such that exactly one full oscillation (period) of the microwave field is completed within the time $1/f_{\mathrm{mod}}$, enforced by the external modulator driving frequency. The optical field (pulse) then also completes one full roundtrip within the same time period. Therefore, the tuning is truly fully continuous (as verified also in experiments and simulations using a very fine frequency step), and not constrained by the natural cavity FSR. We should add that it is true that when the system is excited at modulation frequencies very close to the natural cavity repetition rate $f_{\mathrm{mod}} \approx f_{\mathrm{rep},0}$, the generated pulses are shorter and the emission bandwidths are broader due to a resonant enhancement effect (see also Fig. S2 for the resonant cases at the fundamental and second harmonic frequency). We want to emphasize that repetition rate is continuously tunable over the entire experimental modulation frequency range between 4-16 GHz, without any gaps or discontinuities.

Reviewer Comment 3.5 — 5. Is it possible for the authors to apply similar concepts to the widely used rare earth doped fiber lasers? If so, what would be the conditions?

Reply: The Reviewer shared a very interesting idea of applying a similar concept to fiber lasers. Generally, in actively mode-locked fiber lasers, the loss modulation occurs via concentrated optical elements, such as intracavity modulators. Here, only small modulation frequency detunings are possible since otherwise, the pulse is completely out of tune with the modulator after the completion of a roundtrip, and the assumptions underlying Kärtner's model [18] are not valid anymore. Thus, as emphasized in the present manuscript, a crucial ingredient for large detuning is the spatiotemporal gain modulation along the entire laser cavity rather than in a concentrated optical element, realized by co-propagation of the optical pulse and the traveling net gain window. If at all possible, such an approach could be realized in a fiber laser by modulating the pump intensity (e.g., through modulation of the pump laser diode) in a co-propagating fiber laser setup. However, since rare earth doped fiber lasers have extremely long gain recovery times (∼ms), active mode-locking by synchronous pumping is possible only for ultralong fiber lasers featuring low pulse repetition rates. Only few experimental demonstrations of active mode locking in fiber lasers by synchronous pumping exist, and for practical reasons, the active fiber then only constitutes a small section of the ∼km long fiber cavity. Thus, the fiber amplifier again acts as a concentrated optical element (see, e.g., S.V. Smirnov et al., "Shaping of nanosecond pulses in ytterbium fiber lasers by synchronous sine-wave pump modulation", J. Opt. Soc. Am. B 37, 3068 (2020)).

We therefore conclude that such an approach is not practically feasible to be implemented with actively mode-locked fiber lasers.

Reviewer Comment 3.6 — 6. In the comb properties and generalizations section, all the figures are in the Supplementary Information. The authors might consider moving some figures to the Main Text.

Reply: We thank the Reviewer for the relevant comment. However, we were warned by the Editor that the Main Text must be shortened and that we had already reached the maximum number of Figures.

Therefore, the comb properties and generalizations section, along with the relevant figures, can now be found in the Supplementary Information sections S3, S6, S7, and S10.

Response to the reviewers

Reviewer 1

Senica et al have satisfactorily addressed all the issues raised in my previous comments and I have no more questions to raise. Nevertheless, I do have one suggestion for the authors to consider concerning the first two questions raised in my previous comments (one concerns the mode-locking stability and the other concerns the energy breathing ratio). Since the manuscript is mainly on a novel mode-locking mechanism, these two aspects of ultrafast laser dynamics would be worth mentioning in the main text with brief discussion of the phenomena and the underlying mechanism rather than being only in the supplementary material.

Reply: We thank the Reviewer again for all their helpful comments and recommendations, and for supporting the manuscript for publication. As advised by the Reviewer, we have included a brief summary of these two aspects in the Main Text, and added some additional relevant Extended Data Figures at the end of the manuscript. Due to text length limitations, the majority of the additional results and explanations remains in the Supplementary Information.

Reviewer 2

I believe the manuscript with its modifications really have improved and I support publication. The change in the title and the additions in the main text more accurately capture the essence of the result and clarify the scope compared also to other technoloogies. The extra supplementary material is very helpful for a wide audience. I understood better the implications thanks to the answers to the comments, and appreciate the great effort put in answering the questions. In particular the simple spectroscopy experiment strengthens the case of possible 'usefulness' in spit of the parameters still being not perfectly in the state of the art in absolute values. Although it is still a simple 'proof of concept' it is already meaningful given the sources are a new class of sources.

Reply: We thank the Reviewer again for all their insightful comments and suggestions, especially about the practical applicability of our system, which has encouraged us to develop the proof-of-principle spectroscopy experiment and improve the manuscript, and for their support for publication.

Reviewer 3

All my previous concerns have been adequately addressed by the authors. Therefore, I recommend the manuscript for publication.

Reply: We thank the Reviewer again for all their previous helpful comments and recommendations, and for supporting the manuscript for publication.
